# Risk of any hypoglycaemia with newer antihyperglycaemic agents in patients with type 2 diabetes: A systematic review and meta‐analysis

**DOI:** 10.1002/edm2.100

**Published:** 2019-11-13

**Authors:** Sanaz Kamalinia, Robert G. Josse, Patrick J. Donio, Lindsay Leduc, Baiju R. Shah, Sheldon W. Tobe

**Affiliations:** ^1^ Institute of Medical Sciences University of Toronto Toronto ON Canada; ^2^ St. Michael's Hospital Toronto ON Canada; ^3^ Department of Medicine University of Toronto Toronto ON Canada; ^4^ Northern Ontario School of Medicine Sudbury ON Canada; ^5^ Sunnybrook Research Institute Toronto ON Canada

**Keywords:** diabetes mellitus, type 2, dipeptidyl peptidase IV inhibitor, glucagon‐like peptide‐1 receptor agonist, hypoglycaemia, sodium glucose co‐transporter 2 inhibitor

## Abstract

**Objectives:**

For patients with type 2 diabetes, newer antihyperglycaemic agents (AHA), including the dipeptidyl peptidase IV inhibitors (DPP4i), glucagon‐like peptide‐1 receptor agonists (GLP1RA) and sodium glucose co‐transporter 2 inhibitors (SGLT2i) offer a lower risk of hypoglycaemia relative to sulfonylurea or insulin. However, it is not clear how AHA compare to placebo on risk of any hypoglycaemia. This study evaluates the risk of any and severe hypoglycaemia with AHA and metformin relative to placebo.

**Design:**

A systematic review and meta‐analysis was conducted of randomized, placebo‐controlled trials ≥12 weeks in duration. MEDLINE, Embase and the Cochrane Library were searched up to April 16, 2019. Studies allowing use of other diabetes medications were excluded. Mantel‐Haenszel risk ratio with 95% confidence intervals were used to pool estimates based on class of AHA and number of concomitant therapies used.

**Patients:**

Eligible studies enrolled patients with type 2 diabetes ≥18 years of age.

**Results:**

144 studies met our inclusion criteria. Any hypoglycaemia was not increased with AHA when used as monotherapy (DPP4i (RR 1.12; 95% CI 0.81‐1.56), GLP1RA (1.77; 0.91‐3.46), SGLT2i (1.34; 0.83‐2.15)), or as add‐on to metformin (DPP4i (0.95; 0.67‐1.35), GLP1RA (1.24; 0.80‐1.91), SGLT2i (1.29; 0.91‐1.83)) or as triple therapy (1.13; 0.67‐1.91). However, metformin monotherapy (1.73; 1.02‐2.94) and dual therapy initiation (3.56; 1.79‐7.10) was associated with an increased risk of any hypoglycaemia. Severe hypoglycaemia was rare not increased for any comparisons.

**Conclusions:**

Metformin and the simultaneous initiation of dual therapy, but not AHA used alone or as single add‐on combination therapy, was associated with an increased risk of any hypoglycaemia relative to placebo.

## Graphical abstract

Risk of any hypoglycaemia with newer antihyperglycaemic agents is not increased relative to placebo if used as monotherapy, dual therapy or triple therapy. Risk of any hypoglycaemia is increased relative to placebo with metformin monotherapy and when two agents are initiated simultaneously. Risk of severe hypoglycaemia is extremely rare and similar to placebo.

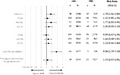

## 1 INTRODUCTION

For patients with type 2 diabetes and their physicians, fear of hypoglycaemia limits attainment of glycaemic targets,^1^, ^2^ increasing the risk of developing diabetes‐related complications.^3^ The last decade has witnessed a dramatic shift favouring the use of three newer classes of antihyperglycaemic agents (AHA) including the dipeptidyl peptidase IV inhibitors (DPP4i), glucagon‐like peptide‐1 receptor agonists (GLP1RA) and sodium glucose co‐transporter 2 inhibitors (SGLT2i).^4^ For patients with type 2 diabetes, these AHA lower blood glucose with the promise of lower hypoglycaemia risk.

Certainly, relative to sulfonylurea (SU) or insulin, the lower risk of hypoglycaemia with AHA is clear and widely accepted.^5^, ^6^, ^7^, ^8^, ^9^ However, relative to placebo, efficacy‐focused studies have been unable to delineate hypoglycaemia risk with these newer AHA, mainly due to the use of background SU and insulin. For instance, a number of systematic review and meta‐analyses have found a significantly higher risk of hypoglycaemia relative to placebo. To explain the increased risk with DPP4i,^10^, ^11^, ^12^, ^13^, ^14^, ^15^ GLP1RA^14^, ^15^, ^16^, ^17^, ^18^, ^19^ and SGLT2i,^9^, ^20^, ^21^, ^22^ authors have pointed to studies allowing background SU or insulin,^17^, ^22^ have conducted post hoc sensitivity analyses to exclude studies with SU or insulin^11^, ^12^, ^13^, ^14^, ^16^, ^18^, ^20^, ^21^, ^23^ or have left the findings unaddressed.^9^, ^15^, ^19^ Thus, a meta‐analysis with hypoglycaemia of newer AHA as the primary objective which a priori excludes studies allowing other background agents is necessary.

The unique mechanism of action of each class of AHA provides a low risk of hypoglycaemia.^24^, ^25^, ^26^ SGLT2i's augment glycosuria in a glucose‐dependent manner.^27^ Incretin‐based therapies, DPP4i and GLP1RA, increase glucagon‐like peptide 1 (GLP1) which in turn stimulates pancreatic insulin secretion in a glucose‐dependent manner.^28^, ^29^ Moreover, the enzyme DPP4 cleaves substrates beyond GLP1 including gastric inhibitory peptide (GIP).^30^, ^31^ Known to enhance glucagon counterregulation during hypoglycaemia, increased GIP with DPP4i may provide additional protection from hypoglycaemia risk.^32^ Unlike the newer AHA, metformin's mechanism of action is not believed to be glucose‐dependent. Hence, each class of AHA presents with a unique mechanism of action which may lead to differing risk of inflicting hypoglycaemia.

For severe hypoglycaemia, we anticipate the risk with AHA to be negligible given their glucose‐dependent mechanisms of action. Further, the strict inclusion criteria of randomized controlled trials make it unlikely that high‐risk patients, many of whom would also be at risk of experiencing a severe episode, would be enrolled. Nevertheless, given the clinical significance of a severe hypoglycaemia episode, its inclusion as an outcome is necessary. But despite its more frequent occurrence, little is known about less severe, mild to moderate or nonsevere hypoglycaemia.^33^ Nonsevere hypoglycaemia episodes increase the risk of subsequent^34^ and more severe events,^35^ direct and indirect costs, frequency of blood glucose monitoring and reduce work productivity and medication adherence.^36^, ^37^, ^38^, ^39^ Moreover, given the progressive nature of diabetes^40^ and involvement of multiple organs,^41^ patients eventually require multiple AHA to maintain glycemic control. Thus, refining our understanding of any hypoglycaemia risk with AHA, particularly when used as dual or triple therapy is of clinical importance.

To date, the heterogeneity of hypoglycaemia definitions, especially of nonsevere events, has deterred comparative research on this important adverse outcome. In 2005, an overarching definition of hypoglycaemia was suggested as; “all episodes of an abnormally low plasma glucose concentration that exposes the individual to potential harm”.^42^ It has been argued that a single definition to encompass all the varying severities, blood glucose values, symptom perception, monitoring, reporting and ascertainment of events is not appropriate.^43^ Following the Diabetes Complication and Control Trial (DCCT), severe hypoglycaemia has typically been defined as requiring external assistance^44^ with or without blood glucose documentation. The International Hypoglycaemia Study Group, in a joint position statement with the American Diabetes Association (ADA) and the European Association for the Study of Diabetes (EASD) now recommends a blood glucose concentration of <3.9 mmol/L to denote an alert value, <3.0 mmol/L to indicate a serious episode, and requiring external assistance, a severe episode.^45^

Systematic review and meta‐analyses allow for sufficient power to evaluate low‐frequency outcomes. However, pooling estimates of rare adverse events compared to efficacy end‐points presents with its own unique challenges.^46^ For instance, consensus is lacking on the optimal pooling methods and handling of studies with zero events in both arms.^47^ Some argue that studies with zero events lack information and its inclusion may negate an otherwise statistical finding.^48^ Others claim exclusion of studies with zero events does not consider all the available evidence and may overestimate risk.^49^ Recently, inclusion of studies with zero events has fostered support^50^ and providing results of both analyses has been recommended.^48^

In this systematic review and meta‐analysis, we evaluate the risk of any and severe hypoglycaemia in patients with type 2 diabetes relative to placebo in studies which only permit the use of metformin, DPP4i, GLP1RA or SGLT2i administered alone or in any combination with each other.

## 2 MATERIALS AND METHODS

The protocol for this meta‐analysis is registered with the International Prospective Register of Systematic Reviews (PROSPERO), number CRD42018095458 and follows the 2015 Preferred Reporting Items for Systematic Review and Meta‐Analysis Protocols (PRISMA‐P) guidelines^51^ as well as PRISMA harms.^47^

### 2.1 Data sources and searches

We undertook a systematic review and meta‐analysis of randomized controlled trials published in the English language. Electronic searches of MEDLINE (since 1946), Embase (since 1947) and the Cochrane Library were conducted from inception up to 16 April 2019. References of relevant studies were also manually searched. Validated search strings for “randomized controlled trials”,^52^, ^53^ combined with MeSH and text terms for “type 2 diabetes,” along with brand and generic names for AHA were used. As suggested by the PRISMA Harms group,^47^ the term for the harms (ie “hypoglycaemia” or “hypoglycaemia”) was not included in the search string to avoid exclusion of potentially eligible studies reporting on this outcome within a supplementary appendix. The MEDLINE search string can be found in the Appendix S1.^54^

### 2.2 Study selection

Two investigators (SK and LL) conducted independent title and abstract screening. If the study met eligibility criteria or if it was unclear, full text of the article was assessed for eligibility. A third reviewer (SWT) was approached for any unresolved disagreements. Only data from the initial study phase where double blinding was maintained was eligible. For studies with multiple or companion publications, only the primary reference or the reference reporting on hypoglycaemia was considered.

Studies were considered for inclusion if they were as follows: (a) randomized, placebo‐controlled trials, (b) conducted in patients with type 2 diabetes ≥18 years of age, (c) evaluated hypoglycaemia risk, (d) with metformin, DPP4i, GLP1RA or SGLT2i as monotherapy or any combination of these AHA and (e) were ≥12 weeks in duration. A minimum 12‐week duration was selected to reflect the efficacy (ie, HbA1c lowering) focus typical of most studies. Exclusion criteria included studies that were as follows: (a) cross‐over design, (b) conducted in healthy individuals or patients with type 1 diabetes, (c) compared to active‐control only, (d) were less than 12 weeks in duration and (e) allowed the use of any other AHA as background therapy such as acarbose, bile‐acid sequestrants, bromocriptine, insulin, meglitinides, SU or thiazolidinediones.

### 2.3 Data extraction and quality assessment

Two reviewers (SK and PJD) independently extracted study and patient characteristics of included studies in a prepiloted table. Differences were resolved through consensus. The primary outcome of any hypoglycaemia was captured irrespective of definition, severity, time of day, blood glucose value or documentation. The secondary outcome of severe hypoglycaemia was defined based on recent recommendations as a blood glucose value of <3.0 mmol/L (<54 mg/dL),^45^ or described as major, or as requiring medical or third party assistance.

The Cochrane Risk of Bias (RoB) tool was used to assess the quality of each study using the six domains of selection, performance, detection, attrition and reporting bias. For the seventh domain of “other bias,” we considered the risk of confounding due to use of rescue therapy. Publication bias was assessed using funnel plots of each trials effect size against standard error if ≥10 studies were available per outcome. The overall quality for each outcome was assessed according to the Grading of Recommendations Assessment Development and Evaluation (GRADE) approach^55^ using a Summary of Findings (SoF) table as described in the Cochrane handbook.^52^ By considering the overall RoB, including inconsistency, indirectness and imprecision across studies, a level of certainty was determined for our findings.

### 2.4 Data synthesis and analysis

For the primary and secondary outcomes, we compared the use of metformin and each class of AHA as monotherapy or as each class of AHA added‐on to metformin background relative to placebo. Studies in which a second AHA was added to a nonmetformin background, or initiated dual therapy (ie, two AHA simultaneously administered to treatment naïve or previously treated patients undergoing a washout) or triple therapy (ie, third AHA added to dual background therapy) were evaluated separately.

Using REVMAN 5.3, we pooled the dichotomous outcome of patients experiencing hypoglycaemia using the Mantel‐Haenszel method if ≥2 studies were available per comparison. In anticipation of the heterogeneity of hypoglycaemia, including the differing definitions, study durations, potential molecule‐specific differences and doses, we used the random effects model. Given the harms objective of our study, for the primary analysis, we evaluated the risk ratio and 95% confidence intervals (CI) by considering studies with hypoglycaemia in at least one treatment arm to obtain a more conservative estimate. To allow for the evaluation of all available data, including studies with zero hypoglycaemia in both arms, we conducted an a priori sensitivity analyses using risk difference. We also planned to evaluate the robustness of our findings using different effect measures and analyses methods.

A single pair‐wise comparison was used for dose‐ranging studies. For studies evaluating multiple interventions eligible for inclusion within the same pooled estimate, the shared placebo group was split to avoid a unit‐of‐analysis error.^52^ Tests of statistical heterogeneity were conducted using Chi^2^ and *I* ^2^ with *P* < .05 denoting statistical significance. As suggested by the Cochrane group, we considered heterogeneity to be unimportant if *I* ^2^ = 0%‐40%, moderate if *I* ^2^ = 30%‐60%, substantial if *I* ^2^ = 50%‐90% and considerable if *I* ^2^ = 75%‐100%.^52^

## 3 RESULTS

**Figure 1:**
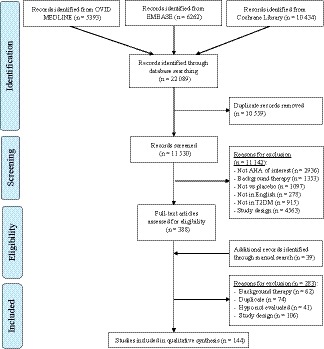
Prisma flow diagram

Of the 22 089 hits retrieved from our search, 144 studies^56^, ^57^, ^58^, ^59^, ^60^, ^61^, ^62^, ^63^, ^64^, ^65^, ^66^, ^67^, ^68^, ^69^, ^70^, ^71^, ^72^, ^73^, ^74^, ^75^, ^76^, ^77^, ^78^, ^79^, ^80^, ^81^, ^82^, ^83^, ^84^, ^85^, ^86^, ^87^, ^88^, ^89^, ^90^, ^91^, ^92^, ^93^, ^94^, ^95^, ^96^, ^97^, ^98^, ^99^, ^100^, ^101^, ^102^, ^103^, ^104^, ^105^, ^106^, ^107^, ^108^, ^109^, ^110^, ^111^, ^112^, ^113^, ^114^, ^115^, ^116^, ^117^, ^118^, ^119^, ^120^, ^121^, ^122^, ^123^, ^124^, ^125^, ^126^, ^127^, ^128^, ^129^, ^130^, ^131^, ^132^, ^133^, ^134^, ^135^, ^136^, ^137^, ^138^, ^139^, ^140^, ^141^, ^142^, ^143^, ^144^, ^145^, ^146^, ^147^, ^148^, ^149^, ^150^, ^151^, ^152^, ^153^, ^154^, ^155^, ^156^, ^157^, ^158^, ^159^, ^160^, ^161^, ^162^, ^163^, ^164^, ^165^, ^166^, ^167^, ^168^, ^169^, ^170^, ^171^, ^172^, ^173^, ^174^, ^175^, ^176^, ^177^, ^178^, ^179^, ^180^, ^181^, ^182^, ^183^, ^184^, ^185^, ^186^, ^187^, ^188^, ^189^, ^190^, ^191^, ^192^, ^193^, ^194^, ^195^, ^196^, ^197^, ^198^, ^199^ met our inclusion criteria (Figure 1). An agreement value (*κ*) of 80% was achieved for studies requiring detailed analysis and extraction. Fourteen of these studies contained multiple intervention arms suitable for inclusion in more than 1 comparison (five studies were included in three comparisons^89^, ^92^, ^106^, ^107^, ^151^ and nine studies in two comparisons^59^, ^86^, ^88^, ^102^, ^128^, ^129^, ^147^, ^159^, ^164^). Only 1 of the 14 studies included a separate placebo arm for each intervention being evaluated.^147^ In 3 of the 14 multi‐intervention studies, the 2 AHA being evaluated were within the same class and thus pooled estimate, necessitating the need for the “shared” placebo to be split.^88^, ^102^, ^128^ Thus, in total, 163 studies (n = 53 713) were pooled for 10 comparisons; 9 studies (n = 2630 participants) in metformin monotherapy, 47 studies (n = 14 926) in DPP4i monotherapy, 11 studies in GLP1RA monotherapy (n = 2705), 19 studies in SGLT2i monotherapy (6647), 29 studies in DPP4i added to metformin (n = 9679), 11 studies in GLP1RA added to metformin (n = 4096), 18 studies in SGLT2i added to metformin (n = 7201), 5 studies in second AHA added to nonmetformin background (n = 953), 6 studies in dual therapy initiation (n = 2215) and 8 studies in triple therapy (n = 2661).

A summary of study and patient characteristics is presented in Table 1. Characteristics of individual studies can be found in the Appendix S1.^54^ In short, most studies were efficacy‐focused with a duration of 12‐24 weeks. Mean age of patients was generally 50‐60 years with the widest range for DPP4i monotherapy and the oldest participants in the triple therapy studies. The majority of SGLT2i monotherapy and just under half of the DPP4i monotherapy studies were conducted in Asian patients. All five studies evaluating a second AHA added to a nonmetformin background were conducted in Japan where metformin is not preferentially recommended first line. Monotherapy studies comprised of participants with lower diabetes duration and baseline haemoglobin A1c (HbA1c). Both baseline HbA1c and change in HbA1c compared to placebo was highest with dual therapy initiation, whereas the smallest change in HbA1c was observed with triple therapy. Considering these differences, between group comparisons of hypoglycaemia risk were avoided.

**Table 1:** Summary of characteristics of included studies

Comparison group	Study duration in weeks (Range)	Mean age (Range)	% Male (Range)	Diabetes duration in years (Range)	Baseline HbA1c (Range)	Change in A1c vs placebo (Range)
Monotherapy						
Metformin	12‐36	52.2‐57.9	41.2‐73.5	1.0‐7.5	7.6‐9.0	–0.55 to –1.3
DPP4i	12‐52	48.9‐72.1	36.4‐86.0	0.5‐8.6	6.7‐9.0	–0.14 to –1.2
GLP1RA	12‐52	51.7‐60.0	31.4‐84.61	1.0‐8.87	7.1‐8.54	–0.38 to –1.85
SGLT2i	12‐52	49.9‐60.6	41.3‐81.8	0.25‐7.8	7.46‐8.45	–0.35 to –1.31
Added to background metformin						
DPP4i	12‐52	51.6‐61.8	41.5‐73.7	0.45‐9.4	7.64‐9.3	–0.30 to –1.1
GLP1RA	12‐52	50.4‐58.9	25.8‐77	0.63‐8.1	7.46‐8.6	–0.1 to –2.1
SGLT2i	12‐26	51.7‐60.8	28.3‐74.5	4.2‐8.1	7.16‐8.46	–0.17 to –1.30
Second AHA added to non‐metformin background	14‐24	54.1‐60.0	68.1‐83.1	6.5‐9.0	7.87‐8.4	–0.82 to –1.14
Dual therapy initiation	24‐26	52.2‐56.4	42.3‐69.7	1.1‐6.8	8.21‐9.0	–1.08 to –2.07
Third AHA added to dual therapy background	24‐26	54.3‐59.7	43.7‐65.4	5.64‐11.62	7.86‐8.5	–0.35 to –0.89

**Figure 2:**
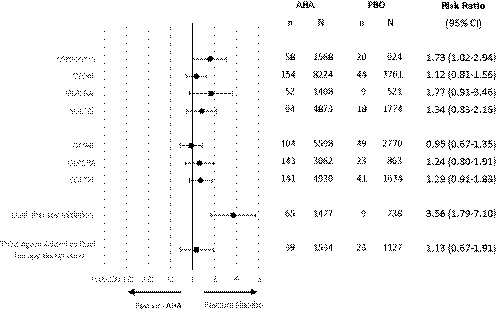
Summary forest plot for risk of any hypoglycaemia

**Figure 3:**
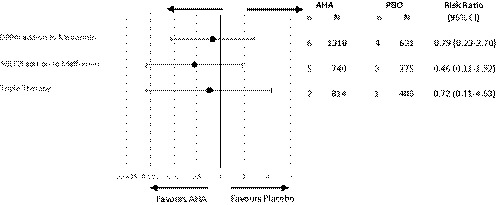
Summary forest plot for risk of severe hypoglycaemia

Forest plots for any and severe hypoglycaemia can be found in the Appendix S1.^54^ In summary, the risk ratio of any hypoglycaemia (Figure 2) was low for all comparisons, occurring in ≤4.7% and ≤2.7% of AHA and placebo, respectively. A significantly higher risk of any hypoglycaemia compared to placebo was found with metformin monotherapy (RR 1.73; 95% CI 1.02‐2.94) and dual therapy initiation (3.56; 1.79‐7.10). The nonmetformin background therapy comparison was not pooled, since only one of the five included studies reported patients with events. Severe hypoglycaemia was rare and reported in less than 10% (15/161) of included studies. In studies reporting severe events, the incidence was similar, occurring in ≤1.0% and ≤1.1% with AHA and placebo, respectively. Only three comparisons had ≥2 studies with severe hypoglycaemia (Figure 3); DPP4i with metformin (RR 0.79; 0.23‐2.70), SGLT2i with metformin (0.46, 0.11‐1.92) and triple therapy (0.72; 0.11‐4.53).

Inclusion of zero event studies decreased the incidence of any hypoglycaemia to ≤4.5% and ≤2.0% for AHA and placebo, respectively, and severe hypoglycaemia to ≤0.2% for either arm. The risk difference of severe hypoglycaemia was not increased with any AHA comparison relative to placebo. However, the risk difference of any hypoglycaemia when zero event studies were included resulted in a small, but statistically significant 1% increase with metformin monotherapy (RD 0.01; 0.0‐0.03), SGLT2i monotherapy (0.01; 0.0‐0.01), GLP1RA with metformin (0.01; 0.0‐0.02) and a 3% increase with dual therapy initiation (0.03; 0.01‐0.05). Results of including zero event studies using risk difference for any and severe hypoglycaemia are presented in the Appendix S1.^54^

Exploratory analyses using different effect measures and analyses methods did not significantly change our findings for severe hypoglycaemia. For any hypoglycaemia, metformin monotherapy and dual therapy initiation remained significantly increased irrespective of the effect measure, use of fixed effect model or inclusion of zero event studies. Use of risk difference without the inclusion of zero event studies resulted in a small but significant 1%‐2% increase in any hypoglycaemia with both fixed and random effects model with GLP1RA monotherapy ([RD, Random 0.02; 95% CI 0.00‐0.03], [RD, Fixed 0.02; 0.00‐0.04]), SGLT2i monotherapy ([RD, Random 0.01; 0.00‐0.01], [RD, Fixed 0.01; 0.00‐0.02]), GLP1RA with metformin ([RD, Random 0.02; 0.01‐0.03], [RD, Fixed 0.01; 0.00‐0.03]) and SGLT2i with metformin ([RD, Random 0.01; 0.00‐0.02], [RD, Fixed 0.01; 0.00‐0.02]). Inclusion of zero event studies using risk difference but with fixed effect model resulted in a small but significant increased risk of any hypoglycaemia for GLP1RA monotherapy (RD, Fixed 0.01; 0.00‐0.03) and SGLT2i with metformin (RD, Fixed 0.01; 0.00‐0.02) while maintaining the small but significantly increased risk observed with SGLT2i monotherapy (RD, Fixed 0.01; 0.00‐0.02) and GLP1RA with metformin (RD, Fixed 0.01; 0.00‐0.03). Use of odds ratio or risk ratio with either fixed or random effect model for any hypoglycaemia when zero event studies were included changed our statistically significant findings to nonsignificant or SGLT2i monotherapy and GLP1RA with metformin.

Risk of bias ratings for each included study is found in the Appendix S1.^54^ None of the included studies were deemed to have a high risk of selection bias resulting from random sequence generation. Most studies, however, did not describe how the sequence was generated. None of the studies were determined as having a high risk of bias for allocation concealment, despite most not providing details on method of concealment. Performance bias was considered low risk, given our inclusion criteria of double‐blind randomized controlled trials. All included studies were ranked high risk for detection bias for two reasons. First, although explicit mention of participant exclusion prerandomization due to history of hypoglycaemia was rare^87^, ^117^, ^166^ patients at high risk of hypoglycaemia may have still been excluded from study enrolment. Second, an adverse event in a placebo‐controlled trial may have been more likely to be attributed to treatment than placebo. For attrition bias, imbalances in the drop‐out rates were carefully considered, given our harms objective and placebo comparator. Safety (and efficacy) end‐points of included studies were reported on an intent‐to‐treat basis. Almost all (140/144) studies reported on drop‐out rates between treatment arms. Of these, one third of studies (42/140 or 30%) were considered high risk of attrition bias, either due to a higher drop‐out rates in the treatment compared to placebo arms^58^, ^63^, ^65^, ^66^, ^67^, ^71^, ^73^, ^75^, ^77^, ^83^, ^85^, ^87^, ^91^, ^93^, ^99^, ^102^, ^104^, ^105^, ^106^, ^131^, ^133^, ^134^, ^139^, ^140^, ^141^, ^151^, ^152^, ^153^, ^161^, ^165^, ^168^, ^170^, ^176^, ^184^, ^185^, ^187^, ^191^, ^195^, ^196^, ^200^ or specific mention of participant study withdrawal due to hypoglycaemia.^58^, ^92^, ^95^, ^131^, ^175^ Six studies were found to have a high risk of bias for selective reporting, either for providing only a range of hypoglycaemia outcomes^76^, ^140^ or reporting hypoglycaemia data only for the extension phase^104^ or insufficient details on the severity of episodes.^56^, ^98^, ^154^

For “other bias,” we considered inclusion of safety data after use of rescue therapy (ie, an additional agent for uncontrolled hyperglycaemia). Close to half (45%) of included studies did not report on rescue medication use making it difficult to determine whether it was permitted and included in the safety analyses. For most of the remaining studies, rescue therapy was permitted with varying criteria and those receiving rescue therapy were excluded from the safety and efficacy analyses. Studies allowing rescue therapy were therefore assigned a low risk of bias. However, in 12% (18/144) of studies, risk of confounding due to rescue therapy was considered high, given inclusion of safety data after initiation of rescue therapy.^58^, ^61^, ^62^, ^65^, ^68^, ^72^, ^83^, ^91^, ^105^, ^107^, ^118^, ^135^, ^141^, ^167^, ^175^, ^186^, ^189^, ^199^ Four studies specifically described hypoglycaemia occurrence after the initiation of rescue therapy and these patients were thus excluded from our pooled estimates. Of these four, a low^155^, ^184^ or unclear^60^, ^79^ risk of bias was assigned depending on whether all hypoglycaemia events were accounted for.

A definition of hypoglycaemia was provided in 63% of studies (95/144). As anticipated, the criteria, classification of severity and ascertainment of hypoglycaemia varied across studies, if reported. Severe hypoglycaemia was mostly defined as requiring assistance (medical or third party) without a need for blood glucose confirmation. Two studies did not describe the severity of hypoglycaemic episodes and were excluded from the pooled estimates of severe hypoglycaemia and assigned a high risk of bias for selective reporting.^56^, ^154^

Given the heterogeneity of hypoglycaemia definitions, we conducted a post hoc sensitivity analysis using a more conservative threshold of ≤3.1 mmol/L for severe events. Results are presented in the Appendix S1.^54^ Sixteen studies defined hypoglycaemia with a threshold of ≤3.1 mmol/L.^57^, ^67^, ^73^, ^78^, ^90^, ^125^, ^128^, ^140^, ^143^, ^144^, ^146^, ^149^, ^150^, ^170^, ^174^, ^184^ Hypoglycaemia did not occur in five of these studies. The significance of our findings did not change using this more conservative threshold for severe hypoglycaemia with DPP4i monotherapy (RR 1.03; 95% CI 0.17‐6.15), DPP4i with metformin background (1.10; 0.42‐2.89) and GLP1RA with metformin background (1.03; 0.46‐2.31).

Evidence of heterogeneity was rejected since for the primary and secondary outcomes of any and severe hypoglycaemia, the Chi^2^ *P*‐value was >.05 for all comparisons. Further, *I* ^2^ values were zero, suggesting unimportant heterogeneity between sample estimates. Between study variance Tau^2^ was also zero for all comparisons in the primary analysis. However, when studies with zero events were included for the outcome of any hypoglycaemia, *I* ^2^ changed to unimportant (37% for metformin monotherapy, 13% for GLP1RA monotherapy, 11% for SGLT2i monotherapy, 6% for GLP1RA with metformin background) and substantial (68% for dual therapy initiation) heterogeneity. For comparison with 10 or more studies included, evidence of funnel plot asymmetry was not observed. Heterogeneity statistics and funnel plots are presented in the Appendix S1.^54^

Summary of Findings (SoF) table can be found in the Appendix S1.^54^ Certainty for any hypoglycaemia was downgraded once to moderate for all comparisons based on potential detection bias of a harm outcome in placebo‐controlled trial. A second downgrade resulting in a low degree of certainty was applied to metformin monotherapy, GLP1RA monotherapy, GLP1RA with metformin background and triple therapy due to concerns of attrition bias. However, for severe hypoglycaemia, a high degree of certainty was found for all comparisons, given its rare occurrence and consistency of results with other effect measures.

## 4 DISCUSSION

In this systematic review and meta‐analysis, the risk of any hypoglycaemia with newer AHA was not increased relative to placebo when used alone, with metformin background or as triple therapy. However, use of metformin monotherapy as well as the simultaneous addition of 2 AHA was associated with a small increased risk of any hypoglycaemia. Further, this study reaffirms the extremely low risk of severe hypoglycaemia with metformin or any AHA.

All three classes of AHA including DPP4i, GLP1RA and SGLT2i have each described their own unique glucose‐dependent mechanism of action and hypoglycaemia counterregulation whereby the risk of hypoglycaemia is minimized. However, metformin's mechanism of action is not fully understood and its lower risk of hypoglycaemia has only been discussed in relation to SU or insulin.^201^ Our findings corroborate those seen in the United Kingdom Diabetes Study (UKPDS) where metformin was found to have a higher rate of hypoglycaemia relative to diet alone.^202^ Nevertheless, metformin remains the most trusted therapy for patients with type 2 diabetes around the world. It is not clear why dual therapy initiation of AHA was found to have an increased risk of any hypoglycaemia relative to placebo but studies evaluating intensive glycemic lowering have all shown an increased risk of hypoglycaemia, albeit with older medications known to increase hypoglycaemia risk.^203^ Of note, all six studies included in the dual therapy initiation group included metformin. Further, participants in the dual therapy initiation group presented with high baseline HbA1c, possibly reflecting a more “difficult‐to‐treat” population. Although it is often assumed that the risk of hypoglycaemia is increased with low baseline HbA1c levels, evidence to suggest high baseline HbA1c as a risk factor for hypoglycaemia is accumulating,^204^, ^205^ and a U‐shaped relationship is likely.^206^

Results of our sensitivity analyses using different risk measures and effect models were exploratory in nature and not adjusted for multiplicity. Nevertheless, more significant results were observed when risk difference was used and moreover when zero event studies were excluded. However, despite allowing for the inclusion of zero event studies, use of risk difference for rare outcomes has been criticized for lacking statistical power.^207^ Additional guidance on the inclusion or exclusion of zero event trials and the optimal statistical pooling methods for patient‐important adverse outcomes is required.

Strengths of our study include being the first systematic review and meta‐analysis specifically designed to evaluate the risk of any hypoglycaemia with three classes of AHA, and metformin relative to placebo in patients with type 2 diabetes. By excluding studies which allow the use of therapies known to increase risk (ie, SU, insulin), we have aimed to improve the estimation of hypoglycaemia risk with newer AHA when used alone or in combination with each other compared to placebo. Second, beyond severe events, we pooled estimates of the more frequent incidence of nonsevere hypoglycaemia. Given the harms focus of our analysis, we conducted post hoc sensitivity analyses using a more conservative blood glucose threshold of ≤3.1 (compared to <3.0 mmol/L). In addition, the inclusion of studies with zero events allowed for risk estimations considering all relevant data. Finally, our study provides hypoglycaemia risk estimates for metformin and AHA mono‐, dual and triple therapy, as well as dual therapy initiation, scenarios commonly seen in the current clinical management of patients with type 2 diabetes.

There were a number of limitations to this study. As anticipated, the definitions of hypoglycaemia were heterogenous and included varying classifications of severity, symptomology, documentation, ascertainment, collection, reporting and handling of patients with hypoglycaemia.^43^, ^208^ To overcome this, our primary outcome was any hypoglycaemia, irrespective of the definitions and classifications used by each study. Future analyses using equivalent, internationally agreed upon definitions and ascertainment of hypoglycaemia, especially of nonsevere events, are needed. We included only studies published in the English language and evaluation of hypoglycaemia risk with AHA from unpublished studies (ie, the grey literature) and studies published in other languages is warranted. In addition, generalizations of our findings from controlled clinical trials to the real‐world may be limited as patients at risk of hypoglycaemia may have been excluded from clinical trial enrolment or lost to follow‐up. Further, all but one study^194^ was sponsored by the pharmaceutical industry. However, a recent Cochrane review found that while pharmaceutical sponsored studies were more likely to report on favourable efficacy outcomes, they were not necessarily more likely to report on more favourable safety outcomes, compared to nonpharmaceutical sponsored studies.^209^ Finally, many older patients and those with a longer duration of diabetes often fail to perceive symptoms of hypoglycaemia.^210^ Since some studies relied on patient reports of hypoglycaemia without blood glucose documentation, asymptomatic events may have been missed. Future studies using new continuous glucose monitoring devices will play a key role in improving our evaluation of hypoglycaemia risk, particularly of asymptomatic events, both in the real‐world and clinical trial setting.

In conclusion, in patients with type 2 diabetes, the risk of any hypoglycaemia was increased relative to placebo with metformin monotherapy and dual therapy initiation, but not with newer AHA used as mono‐, dual or triple therapy. Risk of severe hypoglycaemia is extremely low and similar to placebo with metformin and newer AHA.

## CONFLICT OF INTEREST

Ms Kamalinia initiated her Master's degree while an employee of Merck Canada Inc. with educational support but has since left to focus on her studies. Merck Canada Inc did not play any role in the conception of the research question, protocol development, data synthesis or interpretation. Dr RGJ reports personal fees from Merck, AZ, Novo Nordisk, Lilly/BI and Janssen, outside the submitted work. Ms LL, Mr PJD and Dr BRS declare no competing interests. Dr SWT reports grants from Eli Lilly, Astra Zeneca, AbbVie and Bayer for participation in international contract research studies as well as honoraria for speaker's bureaus from Servier and Valeant during the conduct of the study and has had travel reimbursed by the Novartis Foundation.

## AUTHOR CONTRIBUTIONS

SWT conceived the research question. SK developed the protocol and search strategy. SK and LL selected studies. SK and PJD extracted study level data. SK wrote the first draft and all authors provided review, revision and approval of the final draft.

## RESEARCH INVOLVING HUMAN PARTICIPANTS AND/OR ANIMALS AND INFORMED CONSENT

Ethics approval and patient consent were not required for this analysis.

## Data Availability

The data that support the findings of this study are openly available in https://doi.org/10.5683/SP2/0QDTJX
