# Supplementary material for: Risk of any hypoglycaemia with newer antihyperglycaemic agents in patients with type 2 diabetes: A systematic review and meta‐analysis
Source: Endocrinol Diabetes Metab. 2019 Nov 13;3(1):e00100. doi: 10.1002/edm2.100 (PMC6947712; doi:10.1002/edm2.100)
Supplement: Supplementary file 1 [file EDM2-3-e00100-s001.docx]

**Supplementary Appendix**

Supplemental Table 1: Medline Search String

Supplemental Table 2: Characteristics of Included Studies

Supplemental Figure 1: Risk Ratio of Any Hypoglycemia

Supplemental Figure 2: Risk Ratio of Severe Hypoglycemia

Supplemental Figure 3: Funnel Plot

Supplemental Figure 4: Risk Difference of Any Hypoglycemia

Supplemental Figure 5: Risk Difference of Severe Hypoglycemia

Supplemental Table 3: Post Hoc Analysis if SH used ≤ 3.1 mmol/L

Supplemental Table 4: Summary of Findings Table (SoF)

APPENDIX

**Supplemental Table 1: Medline Search String**

| 1. randomized controlled trial.pt. | 2. controlled clinical trial.pt. | 3. randomized.ab. |
| --- | --- | --- |
| 4. placebo.ab. | 5. clinical trials as topic.sh. | 6. randomly.ab. |
| 7. trial.ti. | 8. 1 or 2 or 3 or 4 or 5 or 6 or 7 | 9. exp animals/ not humans.sh. |
| 10. 8 not 9 | 11. exp Diabetes Mellitus, Type 2/ | 12. (type adj2 ("2" or "II" or "two") adj2 (diabet* or DM)).mp. |
| 13. ((noninsulin or (non adj1 insulin)) adj2 depend* adj2 diabet*).mp. | 14. NIDDM*.mp. | 15. T2D*.mp. |
| 16. or/11-15 | 17. Biguanides/ | 18. exp metformin/ |
| 19. biguanide*.mp. | 20. metformin*.mp. | 21. metaformin*.mp. |
| 22. dimethylbiguanid*.mp. | 23. dimethylguanid*.mp. | 24. Glifor*.mp. |
| 25. metfogamma*.mp. | 26. Siofor*.mp. | 27. Diaformin*.mp. |
| 28. Diabex*.mp. | 29. Dianben*.mp. | 30. Gluformin*.mp. |
| 31. Obimet*.mp. | 32. Glumetza*.mp. | 33. Fortamet*.mp. |
| 34. Riomet*.mp. | 35. Carbophage*.mp. | 36. Glucophage*.mp. |
| 37. or/17-36 | 38. exp Dipeptidyl Peptidase 4/ | 39. exp Dipeptidyl-Peptidase IV Inhibitors/ |
| 40. ((dipeptidylpeptidase* or (dipeptidyl adj1 peptidase*) or dpp*) adj2 ("4" or "iv" or "four") adj2 inhibit*).mp. | 41. gliptin*.mp. | 42. exp sitagliptin phosphate/ |
| 43. sitagliptin*.mp. | 44. januvia*.mp. | 45. janumet*.mp. |
| 46. tesavel*.mp. | 47. xelevia*.mp. | 48. MK0431.mp. |
| 49. (MK adj2 "0431").mp. | 50. istavel*.mp. | 51. istamet*.mp. |
| 52. saxagliptin*.mp. | 53. onglyza*.mp. | 54. BMS477118.mp. |
| 55. (BMS adj2 "477118").mp. | 56. kombiglyze*.mp. | 57. exp linagliptin/ |
| 58. linagliptin*.mp. | 59. trajenta*.mp. | 60. tradjenta*.mp. |
| 61. jentadueto*.mp. | 62. jenttadueto*.mp. | 63. Ondero*.mp. |
| 64. BI1356.mp. | 65. (BI adj2 "1356").mp. | 66. alogliptin*.mp. |
| 67. allogliptin*.mp. | 68. SYR322.mp. | 69. (SYR adj2 "322").mp. |
| 70. kazano*.mp. | 71. nesina*.mp. | 72. vipidia*.mp. |
| 73. vildagliptin*.mp. | 74. galvus*.mp. | 75. jalra*.mp. |
| 76. xiliarx*.mp. | 77. LAF237.mp. | 78. (LAF adj2 "237").mp. |
| 79. eucreas*.mp. | 80. galvumet*.mp. | 81. teneligliptin*.mp. |
| 82. tenelia*.mp. | 83. MP513.mp. | 84. (MP adj2 "513").mp. |
| 85. dutogliptin*.mp. | 86. PHX1149T.mp. | 87. (PHX adj2 1149T).mp. |
| 88. omarigliptin*.mp. | 89. MK3102.mp. | 90. (MK adj2 "3102").mp. |
| 91. gemigliptin*.mp. | 92. LC150444.mp. | 93. (LC adj2 "150444").mp. |
| 94. (LC15 adj2 "0444").mp. | 95. anagliptin*.mp. | 96. suiny*.mp. |
| 97. K726J96838.mp. | 98. evogliptin*.mp. | 99. suganon*.mp. |
| 100. DA1229.mp. | 101. (DA adj2 "1229").mp. | 102. retagliptin*.mp. |
| 103. SP2086.mp. | 104. (SP adj2 "2086").mp. | 105. carmegliptin*.mp. |
| 106. camegliptin*.mp. | 107. R1579.mp. | 108. RG1579.mp. |
| 109. (RG adj2 "1579").mp. | 110. RO4876904.mp. | 111. (RO adj2 "4876904").mp. |
| 112. trelagliptin*.mp. | 113. zafatek*.mp. | 114. SYR472.mp. |
| 115. (SYR adj2 "472").mp. | 116. bisegliptin*.mp. | 117. denagliptin*.mp. |
| 118. GW823093.mp. | 119. (GW adj2 "823093").mp. | 120. (psn adj2 "9301").mp. |
| 121. psn9301.mp. | 122. gosogliptin*.mp. | 123. PF00734200.mp. |
| 124. (PF adj2 "00734200").mp. | 125. (PF adj2 "734200").mp. | 126. melogliptin*.mp. |
| 127. GRC8200.mp. | 128. (GRC adj2 "8200").mp. | 129. septagliptin*.mp. |
| 130. or/38-129 | 131. exp Glucagon-Like Peptide 1/ | 132. exp Glucagon like peptide 1 receptor/ |
| 133. (((glucagon adj1 like adj1 peptide) or GLP) adj2 ("1" or "one") adj4 (agonist* or analog* or RA*)).mp. | 134. exp liraglutide/ | 135. liraglutide*.mp. |
| 136. victoza*.mp. | 137. saxenda*.mp. | 138. NN2211.mp. |
| 139. (NN adj2 "2211").mp. | 140. NNC901170.mp. | 141. (NNC adj1 "90" adj1 "1170").mp. |
| 142. (NNC90 adj1 "1170").mp. | 143. exenatide*.mp. | 144. byetta*.mp. |
| 145. bydureon*.mp. | 146. AC2993.mp. | 147. (AC adj2 "2993").mp. |
| 148. AC3174.mp. | 149. (AC adj2 "3174").mp. | 150. ITCA650.mp. |
| 151. (ITCA adj2 "650").mp. | 152. lixisenatide*.mp. | 153. lyxumia*.mp. |
| 154. adlyxin*.mp. | 155. AVE0010.mp. | 156. (AVE adj2 "0010").mp. |
| 157. ZP10A.mp. | 158. (ZP adj2 "10A").mp. | 159. dulaglutide*.mp. |
| 160. trulicity*.mp. | 161. LY2189265.mp. | 162. (LY adj2 "2189265").mp. |
| 163. albiglutide*.mp. | 164. tanzeum*.mp. | 165. GSK716155.mp. |
| 166. (GSK adj2 "716155").mp. | 167. eperzan*.mp. | 168. taspoglutide*.mp. |
| 169. (BIM adj2 "51077").mp. | 170. BIM51077.mp. | 171. (ITM077 or (ITM adj2 "077") or R1583 or (R adj2 "1583") or RO5073031 or (RO adj2 "5073031")).mp. |
| 172. (RO adj2 "5073031").mp. | 173. RO5073031.mp. | 174. (R adj2 "1583").mp. |
| 175. R1583.mp. | 176. (ITM adj2 "077").mp. | 177. ITM077.mp. |
| 178. semaglutide*.mp. | 179. ozempic*.mp. | 180. NN9535.mp. |
| 181. (NN adj2 "9535").mp. | 182. elsiglutide*.mp. | 183. albenatide*.mp. |
| 184. cjc1131.mp. | 185. "cjc ad2 1131".mp. | 186. efpeglenatide*.mp. |
| 187. HM11260C.mp. | 188. (HM adj2 "11260C").mp. | 189. LY307161.mp. |
| 190. (LY adj2 "307161").mp. | 191. pegapamodutide*.mp. | 192. or/131-191 |
| 193. exp Sodium-Glucose Transport Proteins/ | 194. exp sodium-glucose transporter 2/ | 195. gliflozin*.mp. |
| 196. (sodium* adj4 glucose* adj4 (cotransport* or (co adj2 transport*) or transport*) adj4 inhibit*).mp. | 197. (sglt* adj4 inhibit*).mp. | 198. exp Canagliflozin/ |
| 199. canagliflozin*.mp. | 200. invokana*.mp. | 201. JNJ24831754.mp. |
| 202. (JNJ adj2 "24831754").mp. | 203. TA7284.mp. | 204. (TA adj2 "7284").mp. |
| 205. invokamet*.mp. | 206. dapagliflozin*.mp. | 207. farxiga*.mp. |
| 208. forxiga*.mp. | 209. xigduo*.mp. | 210. BMS512148.mp. |
| 211. (BMS adj2 "512148").mp. | 212. qtern*.mp. | 213. empagliflozin*.mp. |
| 214. jardiance*.mp. | 215. synjardy*.mp. | 216. (BI adj2 "10773").mp. |
| 217. BI10773.mp. | 218. BI4487.mp. | 219. (BI adj2 "4487").mp. |
| 220. glyxambi*.mp. | 221. ertugliflozin*.mp. | 222. PF049717129.mp. |
| 223. (PF adj2 "049717129").mp. | 224. MK8835.mp. | 225. (MK adj2 "8835").mp. |
| 226. steglujan*.mp. | 227. steglatro*.mp. | 228. segluromet*.mp. |
| 229. steglaro*.mp. | 230. ipragliflozin*.mp. | 231. suglat*.mp. |
| 232. ASP1941.mp. | 233. (ASP adj2 "1941").mp. | 234. remogliflozin*.mp. |
| 235. GSK189075.mp. | 236. (GSK adj2 "189075").mp. | 237. tofogliflozin*.mp. |
| 238. apleway*.mp. | 239. deberza*.mp. | 240. CSG452.mp. |
| 241. (CSG adj2 "452").mp. | 242. R7201.mp. | 243. (R adj2 "7201").mp. |
| 244. atigliflozin*.mp. | 245. bexagliflozin*.mp. | 246. EGT0001474.mp. |
| 247. (EGT adj2 "0001474").mp. | 248. EGT0001442.mp. | 249. (EGT adj2 "0001442").mp. |
| 250. EGT1442.mp. | 251. (EGT adj2 "1442").mp. | 252. luseogliflozin*.mp. |
| 253. lusefi*.mp. | 254. (TS adj2 "071").mp. | 255. TS071.mp. |
| 256. sergliflozin*.mp. | 257. GW869682X.mp. | 258. (GW adj2 869682X).mp. |
| 259. sotagliflozin*.mp. | 260. lx4211.mp. | 261. (LX adj2 "4211").mp. |
| 262. velagliflozin*.mp. | 263. (ISIS adj2 "388626").mp. | 264. ISIS388626.mp. |
| 265. (ISIS adj2 SGLT2Rx).mp. | 266. henagliflozin*.mp. | 267. SHR3824.mp. |
| 268. (SHR adj2 "3824").mp. | 269. mizagliflozin*.mp. | 270. GSK1614235.mp. |
| 271. (GSK adj2 "1614235").mp. | 272. BI44847.mp. | 273. (BI adj2 "44847").mp. |
| 274. or/193-273 | 275. 37 or 130 or 192 or 274 | 276. 10 and 16 and 275 |

**Supplemental Table 2: Characteristics of Included Studies**

**Characteristics of Included Studies – Metformin Monotherapy**

| **Study ID** | **Dose** | **Study Duration** | **n=** | **Mean Age (SD)** | **Gender (% male)** | **Countries Studied** | **Ethnicity (%)** | **Duration of Diabetes in Years (SD)** |
| --- | --- | --- | --- | --- | --- | --- | --- | --- |
| Chiasson JL 2001 | met 500mg TID | 36 weeks | met 500mg TID: 83; pbo: 83 | met 500mg TID: 57.9 (8.6); pbo: 57.7 (9.9) | met 500mg TID: 73.5; pbo: 67.5 | Canada | met 500mg TID: Caucasian 88, Black 1.2, Asian 7.2, Other 3.6; pbo: Caucasian 91.6, Black 1.2, Asian 4.8, Other 2.4 | met 500mg TID: 7.5 (7.4); pbo: 5.1 (4.9) |
| DeFronzo RA 1995 | met 850mg TID | 29 weeks | met 850mg TID: 143; pbo: 146 | met 850mg TID: 53 (1); pbo: 53 (1) | met 850mg TID: 43.4; pbo: 42.5 | USA | White, Black, Hispanic (*n* not given) | met 850mg TID: 6.0 (0.5); pbo: 6.0 (0.6) |
| Fonseca VA 2013 | met 1500mg QD | 12 weeks | met 1500mg: 69; pbo: 69 | met 1500mg: 53.1 (11.7); pbo: 53.4 (9.7) | met 1500mg 58; pbo: 46.4 | USA, Mexico, Columbia, India, Philippines | met 1500mg: White 46.4, non-White 53.6; pbo: White 47.8, non-White 52.2 | met 1500mg: 4.13 (4.71); pbo: 4.64 (5.93) |
| Goldstein BJ 2007 | met 500mg BID; met 1000mg BID | 24 weeks | met 500mg bid: 182; met 1000mg bid: 182; placebo: 176 | met 500mg bid: 53.4 (10.2); met 1000mg bid: 53.2 (9.6); placebo: 53.6 (10.0) | met 500mg bid: 48.9; met 1000mg bid: 45.1; placebo: 52.8 | multinational; not specified | met 500mg bid: White 47.8, Black 6.6, Hispanic 30.2, Asian 7.7, Other 7.7; met 1000mg bid: White 58.2, Black 4.9, Hispanic 21.4, Asian 5.5, Other 9.9; placebo: White 46.0, Black 9.7, Hispanic 26.7, Asian 6.8, Other 10.8 | met 500mg bid: 4.5 (3.9); met 1000mg bid: 4.4 (4.4); placebo: 4.6 (4.9) |
| Haak T 2012 | met 500mg BID; met 1000mg BID | 24 weeks | met 500mg bid: 144; met 1000mg bid: 147; pbo: 72 | met 500mg bid: 52.9 (10.4); met 1000mg bid: 55.2 (10.6); pbo: 55.7 (11.0) | met 500mg bid: 56.9; met 1000mg bid: 53.1; pbo: 50.0 | 14 countries; not specified | met 500mg bid: White 64.6, Asian 35.4, Black 0, Hawaiian/Pacific Islander 0.0; met 1000mg bid; White 64.6, Asian 34.0, Black 1.4, Hawaiian/Pacific Islander 0.0; pbo: white 63.9, Asian 36.1, Black 0, Hawaiian/Pacific Islander 0.0 | met 500mg BID: ≤1: 41.1, > 1 to 5: 35.5, > 5: 23.4; met 1000mg BID: ≤1: 34.8, > 1 to 5: 45.7, > 5: 19.6; pbo: ≤1: 30.8, > 1 to 5: 35.4, > 5: 33.8 |
| Ji L 2016 | met 500mg BID; met 850mg BID | 24 weeks | met 500mg BID: 126; met 850mg BID: 124; pbo: 126 | met 500mg BID: 52.6 (9.5); met 850mg BID: 53.0 (10.3); pbo: 53.6 (9.7) | met 500mg BID: 54.8; met 850mg BID: 60.5; pbo: 68.5 | China | met 500mg BID: Asian: 100; met 850mg BID Asian: 100; pbo Asian 100 | met 500mg BID: 1.0 (0.2); met 850mg BID: 1.1 (0.2); pbo 1.1 (0.2) |
| Ji L 2017 | met 500mg BID | 26 weeks | met 500mg BID: 161; pbo: 161 | met 500mg BID: 53.6 (9.91); pbo: 52.2 (10.17) | met 500mg BID: 50.6; pbo: 58.3 | China | met 500mg BID: Asian 99.4, American Indian or Alaskan Native 0, multiracial 0.6; pbo: Asian 98.8, American Indian or Alaskan Native 1.2, Multiracial 0 | not reported |
| List JF  2009 | met XR 1500mg QD | 12 weeks | met XR 1500mg: 56; pbo: 54 | met XR 1500mg: 54 (9); pbo: 53 (11) | met XR 1500mg: 48; pbo: 56 | Canada, Mexico, Puerto Rico, US | not reported | not reported |
| Pratley RE 2014 | met 500mg BID; met 1000mg BID | 26 weeks | met 500mg BID: 114; met 1000mg BID: 111; pbo: 109 | met 500mg BID: 54.6 (10.20); met 1000mg BID: 52.6 (11.30); pbo: 53.1 (9.60) | met 500mg BID: 41.2; met 1000mg BID: 45.9; pbo: 50.5 | worldwide; not specified | [RACE] met 500mg BID: Asian 16.7, Black or African American 5.3, White 74.6, Other 3.5; met 1000mg BID: Asian 18.0, Black or African American 5.4, White 71.2, Other 5.4; pbo: Asian 18.3, Black or African American 7.3, White 69.7, Other 4.6 | met 500mg BID: 3.8 (3.9); met 1000mg BID: 4.1 (4.59); pbo: 4.3 (4.78) |

**Characteristics of Included Studies – Metformin Monotherapy continued**

| **Study ID** | **Baseline A1C % (SD)** | **Mean Change in HbA1C % vs. baseline** | **Mean Difference in HbA1c % vs pbo** | **HYPO def** | **Ascertainment of Hypo** | **Rescue Medication** | **Excl of Pts w events? At screening or during study)** |
| --- | --- | --- | --- | --- | --- | --- | --- |
| Chiasson JL 2001 | met 500mg TID: 8.2 (0.9); pbo: 8.1 (0.7) | met 500mg TID: -0.85 (0.12); pbo: 0.38 (0.12) | met 500mg TID: -1.25 (n/a) | not reported | Patient-reported | not reported | no |
| DeFronzo RA 1995 | met 850mg TID: 8.4 (0.1); pbo: 8.2 (0.2) | met 850mg TID: -1.4 (0.1); pbo: 0.4 (0.1) (not specified as LSM) | not reported | not reported | Report of symptoms compatible with hypoglycemia; no biochemical documentation of hypoglycemia necessary. | not reported | no |
| Fonseca VA 2013 | met 1500mg: 8.03 (0.90); pbo: 7.84 (0.78) | [LSM] met 1500mg: pbo: 0.26 | [LSM] met 1500mg: -0.72 (-0.97 to -0.48) | Serious defined by development of hypoglycemic coma requiring hospitalization or requiring discontinuation of study drug. | Patient-reported (blood glucose unconfirmed) Patients were provided with a glucometer and asked to monitor  capillary blood glucose twice daily (fasted and 2 h after a meal) and also if symptoms of hypoglycemia occurred. | not reported | no |
| Goldstein BJ 2007 | met 500mg BID: 8.9 (1.0); met 1000mg BID: 8.7 (0.9); pbo: 8.7 (1.0) | [LSM] met 500mg BID: -0.82 (-0.98 to -0.66); met 1000mg BID: -1.13 (-1.29 to -0.97); pbo: 0.17 (0.0 to 0.33) | [LSM] metformin 500mg BID: -0.99 (-1.22 to -0.75); metformin 1000mg BID: -1.3 (-1.53 to -1.06) | not reported | not reported | safety and efficacy endpoints excluded data after rescue with glyburide | no |
| Haak T 2012 | met 500mg bid: 8.7 (0.9); met 1000mg bid: 8.5 (0.9); pbo: 8.7 (1.0) | adjusted mean (SE) met 500mg bid: -0.6 (0.1); met 1000mg bid -1.1 (0.1); pbo: 0.1 (0.1) | adjusted mean (SE) met 500mg bid: -0.8 (0.1) (-1.0 to -0.5); met 1000mg bid -1.2 (0.1) (-1.5 to -0.9) | Severe hypoglycemia defined as requiring the assistance of another person to actively administer carbohydrate, glucagon or other. | Reported by the investigator. Hypoglycemic episodes were recorded and analyzed  separately from other AEs. Hypoglycemic event intensity  was graded according to the investigator’s discretion. | The use of rescue therapy (with sulphonylureas, thiazolidinediones or insulin) was permitted. Values obtained after rescue medication was initiated were not used in the LOCF. | unlikely, but hypoglycemic events graded at investigators discretion; criteria not specified and could lead to patient exclusion |
| Ji L  2016 | met 500mg BID: 8.7 (1.0); met 850mg BID: 8.7 (1.1); pbo: 9.0 (1.1) | [LSM] met 500mg BID: -1.29 (-1.54 to -1.04); met 850mg BID: -1.56 (-1.8 to -1.32); pbo: -0.59 (-0.84 to -0.34) | [LSM] met 500mg BID: -0.70 (-1.01 to -0.39); met 850mg BID: -0.97 (-1.28 to -0.66) | Any episode with symptoms consistent with hypoglycemia (e.g., weakness, dizziness, shakiness, increased sweating, palpitations or confusion) was reported as an episode of symptomatic hypoglycemia without a requirement for confirmatory blood glucose values. Asymptomatic hypoglycemia was defined as an  episode without symptoms of hypoglycemia, but with fingerstick glucose level ≤3.9 mmol/L (≤70 mg/dL). Severe hypoglycemia  was defined as any episode requiring assistance, either medical or non-medical. Episodes with a markedly depressed level of consciousness, loss of consciousness or seizure were to be classified as having required medical assistance, whether or not medical assistance was obtained. | not reported | Rescue therapy with open-label glipizide permitted. The primary approach to analyzing safety data treated data obtained after the initiation of rescue therapy as missing; a secondary approach included all data, regardless of rescue therapy. | no |
| Ji L 2017 | met 500mg BID: 8.4 (0.78); pbo: 8.21 (0.77) | [LSM] (SE) met 500mg BID: -1.04 (0.11) (95% CI -0.70 to -0.278); pbo -0.19 (n/a) | [LSM] not reported | not reported | self-monitor blood glucose levels, keep a hypoglycemic diary | Rescue confounding. Hyperglycemic rescue permitted. | no |
| List JF  2009 | met XR 1500mg: 7.6 (0.8); pbo: 7.9 (0.9) | met XR 1500mg: -0.73 (0.1); pbo: -0.18 (0.1) | -0.55  manually calculated | Hypoglycemia was defined as events with a fingerstick glucose ≤2.8 mmol/L. | BG confirmation | not reported | no |
| Pratley RE 2014 | not reported; The majority of patients (60% overall) entered with a baseline  HbA1c of 8.5% or lower. | [LSM] (SE) met 500mg BID: -0.65 (0.094); met 1000mg BID: -1.11 (0.092); pbo: 0.15 (n/a) | [LSM] (SE) manually calculated: met 500mg BID: -0.80; met 1000mg BID: -1.26 | Mild to moderate hypoglycaemia (blood glucose <70 mg/dl (3.89 mmol/l), symptomatic or asymptomatic). All hypoglycemic episodes were associated with a blood glucose <70 mg/dl (3.89 mmol/l). Severe episodes required assistance. | Use of a home glucose  monitor and diary to record hypoglycemic episodes. | Hyperglycemic rescue (SU or other) was permitted. | no |

**Characteristics of Included Studies – DPP4i Monotherapy**

| **Study ID** | **Dose** | **Study Duration** | **n=** | **Mean Age (SD)** | **Gender (% male)** | **Countries Studied** | **Ethnicity (%)** | **Duration of Diabetes in Years (SD)** |
| --- | --- | --- | --- | --- | --- | --- | --- | --- |
| Agarwal P 2018 | teneli 20mg QD; 40mg QD | 16 weeks | teneli: 158; pbo: 79 | teneli: 49.6 (8.79); pbo: 48.9 (7.84) | teneli: 63.9; pbo: 54.4 | India | Indian | not reported |
| Aschner P 2006 | sita 100mg QD; 200mg QD | 24 weeks | sita 100mg: 238; 200mg: 250; pbo: 253 | sita 100mg: 53.4 (9.5); 200mg: 54.9 (10.1); pbo: 54.3 (10.1) | sita 100mg: 57.1; 200mg: 46.8; pbo: 51.4 | USA | sita 100mg: Asian 13.4, Black 4.2, Hispanic 24.4, Caucasian 51.3, other 6.7; 200mg: Asian 14.8, Black 4.8, Hispanic 21.2, Caucasian 52.8, other 6.4; pbo: Asian 13.4, Black 6.3, Hispanic 25.3, Caucasian 50.2, other 4.7 | sita 100mg: 4.3 (4.9); 200mg: 4.3 (4.7); pbo: 4.6 (4.7) |
| Barnett AH 2012 | lina 5mg QD | 18 weeks (extension data not captured) | lina 5mg: 151; pbo: 76 | lina 5mg: 56.4 (10.6); pbo: 56.7 (9.7) | lina 5mg: 36.4; pbo: 43.4 | Canada, Mexico, Philippines, Romania, Russia, Ukraine, and USA. (7 countries) | lina 5mg: White 70.2, Asian 27.8, Other 2.0; pbo: White 67.1, Asian 27.6, Other 5.3 | lina 5mg: ≤1 year 21.8, >1 to 5 years 51.0, >5years 27.2; pbo: ≤1 year 24.7, >1 to 5 years 54.8, >5years 20.5 |
| Barzilai N 2011 | sita 50mg QD; sita 100mg QD depending on renal function as study done in elderly population | 24 weeks | sita: 102; pbo: 104 | sita: 71.6 (6.1); pbo: 72.1 (6.0) | sita: 47; pbo: 47 | USA | sita: White 75, Black 10, Hispanic 9, Asian 3, Other 4; pbo: White 83, Black 9, Hispanic 6, Asian 3, Other 0 | sita: 7.8 (0.8); pbo: 7.8 (0.7) |
| Chen Y 2015 | lina 5mg QD | 24 weeks | lina 5mg: 200; pbo: 99 | lina 5mg: 54.6 (101); pbo: 54.1 (9.3) | lina 5mg: 58; pbo: 59.6 | China, Malaysia, Philippines | [COUNTRY] lina 5mg: Chinese 86.0; Malaysian 7.5; Philippine 6.5; pbo; Chinese 88.9; Malaysian 7.1; Philippine 4.0 | lina 5mg: ≤ 1 year 50.0, > 1-5 years 31.6, > 5 years 18.4; pbo: ≤ 1 year 52.1, > 1-5 years 31.9, > 5 years 16.0[Time since diagnosis, %] |
| DeFronzo 2008 | alo 12.5mg QD or alo 25mg QD | 26 weeks | alo 12.5: 133; alo 25: 131; pbo: 64 | 53.4 (11.1) (no breakdown) | 53.2 | Argentina, Brazil, Chile, Dominican Republic, Guatemala, Hungary, India, Mexico, Netherlands, New Zealand, Poland, South Africa, United Kingdom, United States | White: 66.9 | not reported |
| Dejager S 2007 | vilda 50mg QD; vilda 50mg BID; vilda 100mg QD | 24 weeks | vilda 50mg QD: 163; 50mg BID: 152; 100mg QD: 157; pbo: 160 | vilda 50mg QD: 55.3 (11.4); 50mg BID: 52.8 (9.6); 100mg QD: 53.6 (10.8); pbo: 52.2 (11.2) | vilda 50mg QD: 41.3; 50mg BID: 46.7; 100mg QD: 53.3; pbo: 47.9 | USA, Russia, Tunisia | vilda 50mg QD: Caucasian 73.1, Hispanic/Latino 13.5, Black 9.6, all other 3.8; 50mg BID: Caucasian 3.3, Hispanic/Latino 13.3, Black 10.0, all other 33.4; 100mg QD: Caucasian 76.1, Hispanic/Latino 15.2, Black 4.3, all other 4.4; pbo: Caucasian 69.1, Hispanic/Latino 11.7, Black 12.8, all other 6.4 | vilda 50mg QD: 2.1 (3.6); 50mg BID: 2.1 (3.3); 100mg QD: 2.4 (4.2); pbo: 1.6 (2.5) |
| Del Prato S  2011 | lina 5mg QD | 24 weeks | lina 5mg QD: 336; pbo: 167 | lina 5mg QD: 56.4 (10.1); pbo: 54.4 (10.3) | lina 5mg QD: 48.8; pbo: 47.3 | Croatia, India, Italy, Israel, Malaysia, Poland, Romania, Slovakia, Ukraine, Thailand, The Netherlands | lina 5mg QD: Native 0, Asian 46.4, White 53.6; pbo: Native 0.6, Asian 45.5, White 53.9 | not reported |
| Gantz I 2017 | omari 25mg OW; sita 50mg QD | 24 weeks | sita: 165; pbo: 83 | sita: 60 (9); pbo: 61 (9) | sita: 69.7; pbo: 68.7 | Japan | not reported | sita: 7.4 (5.3); pbo: 8.6 (5.1) |
| Gantz I 2017 | omari 25mg OW; sita 50mg QD | 24 weeks | omari: 166; pbo: 83 | omari: 60 (11); pbo: 61 (9) | omari: 62.7; pbo: 68.7 | Japan | not reported | omari: 7.4 (5.5); pbo: 8.6 (5.1) |
| Goldstein BJ 2007 | sita 100mg QD | 24 weeks | sita 100mg: 179; pbo: 176 | sita 100mg: 53.3 (10.2); pbo: 53.6 (10.0) | sita 100mg: 52.0; pbo: 52.8 | multinational; not specified | [RACE] sita 100mg: White 52.0, Black 6.1, Hispanic 29.1, Asian 3.4, Other 9.5; pbo: White 46.0, Black 9.7, Hispanic 26.7, Asian 6.8, Other 10.8 | sita 100mg: 4.4 (4.6); pbo: 4.6 (4.9) |
| Haak T 2012 | lina 5mg QD | 24 weeks | lina 5mg: 142; pbo: 72 | lina 5mg: 56.2 (10.8); pbo: 55.7 (11.0) | lina 5mg: 56.3; pbo: 50.0 | 14 countries; not specified | lina 5mg: White 68.3, Asian 31.7, Black 0.0, Hawaiian/Pacific Islander 0.0; pbo: White 63.9, Asian 36.1, Black 0.0, Hawaiian/Pacific Islander 0.0 | lina 5mg: ≤1: 40.0, > 1 to 5: 34.8, > 5: 25.2; pbo: ≤1: 30.8, > 1 to 5: 35.4, > 5: 33.8 |
| Hanefeld M 2007 | sita 25mg QD; sita 50mg QD; sita 100mg QD; sita 50mg BID | 12 weeks | sita 25mg QD: n = 111; 50mg QD: n = 112; 100mg QD: n = 110; 50mg BID: n = 111; pbo: 111 | sita 25mg QD: 55.1 (9.6); 50mg QD: 55.3 (10.3); 100mg QD: 56.0 (7.9); 50mg BID: 55.2 (9.5); pbo: 55.9 (9.3) | sita 25mg QD: 51.4; 50mg QD: 45.5; 100mg QD: 55.5; 50mg BID: 44.1; pbo: 63.1 | Germany, Hungary, Iceland, Lithuania, Poland, United Kingdom, United States | [RACE] sita 25mg QD: Asian 0.9, Black 3.6, White 88.3, other 7.2; sita 50mg QD: Asian 0, Black 8.0, White 85.7, other 6.3; sita 100mg QD: Asian 0, Black 5.5, White 88.2, other 6.4; sita 50mg BID: Asian 0.9, Black 6.3, White 81.1, other 11.7; pbo: Asian 0.9, Black 7.2, White 78.4, other 13.5 | sita 25mg QD: 3.6 (3.4); 50mg QD: 3.3 (3.9); 100mg QD: 3.6 (3.9); 50mg BID: 4.5 (5.9); pbo: 3.3 (3.4) |
| Home P 2018 | omari 25mg QW | 24 weeks | omari 25mg QW: 165; pbo: 164 | omari 25mg QW: 57.4 (9.2); pbo: 57.0 (9.7) | omari 25mg QW: 57.6; pbo: 59.1 | Bulgaria, Germany, Hungary, Italy, Netherlands, Philippines, Romania, South Korea, Taiwan, United States | omari 25mg QW: White 68.5, Asian 28.5, Black 2.4, multi-racial 0, American Indian/Alaska Native 0.6; pbo: White 67.7, Asian 26.2, Black 5.5, multi-racial 0.6, American Indian/Alaska Native 0 | omari 25mg QW: 5.4 (3.8); pbo: 5.7 (4.7) |
| Hong S 2016 | teneli 20mg QD | 24 weeks | teneli 20mg: 99; pbo: 43 | teneli 20mg: 56.64 (10.07); pbo: 57.93 (11.90) | teneli 20mg: 52.53; pbo: 65.12 | South Korea | Korean | alo: 4.59; pbo: 4.59 |
| Inagaki N 2014 | trela 12.5mg QW; trela 25mg QW; trela 50mg QW; trela 100mg QW; trela 200mg QW | 12 weeks | trela 12.5mg QW: 54; 25mg QW: 52; 50mg QW: 51; 100mg QW: 55; 200mg QW: 54; pbo: 55 | trela 12.5mg QW: 60.6 (10.24); 25mg QW: 58.5 (10.49); 50mg QW: 61.0 (10.18); 100mg QW: 57.8 (10.38); 200mg QW: 60.5 (11.26); pbo: 61.6 (9.79) | trela 12.5mg QW: 61; 25mg QW: 65; 50mg QW: 53; 100mg QW: 51; 200mg QW: 67; pbo: 66 | Japan | Japanese | trela 12.5mg QW: 7.82; 25mg QW: 5.96; 50mg QW: 5.78; 100mg QW: 6.09; 200mg QW: 6.99; pbo: 6 |
| Inagaki et al. 2015 | alo 25mg QD | 24 weeks | alo 25mg: 92; pbo: 50 | [MEDIAN] alo: 60 (53-65); pbo: 62 (54-67) | alo 25mg: 75; pbo: 86 | Japan | Korean | alo: 7.07; pbo: 7.54 |
| Inagaki et al. 2015 | trela 100mg QW | 24 weeks | trela 100mg QW: 101; pbo: 50 | [MEDIAN] trela 100mg QW: 58 (52-65); pbo: 62 (54-67) | trela 100mg QW: 72; pbo: 86 | Japan | Korean | trela: 6.25; pbo: 7.54 |
| Iwamoto Y 2010 | sita 25mg QD; 50mg QD; 100mg QD; 200mg QD | 12 weeks | sita 25mg QD: n=80; 50mg QD: n=72; 100mg QD: n=70; 200mg QD: n=68; pbo: 73 | sita 25mg QD: 59.9 (7.9); 50mg QD: 60.2 (9.4); 100mg QD: 58.3 (9.5); 200mg QD: 60.6 (7.7); pbo: 60.2 (8.0) | sita 25mg QD: 63.8; 50mg QD: 65.3; 100mg QD: 51.4; 200mg QD: 58.8; pbo: 68.5 | Japan | Japanese | sita 25mg QD: 4.7 (4.3); 50mg QD: 5.6 (6.4); 100mg QD: 5.4 (5.4); 200mg QD: 5.1 (4.9); pbo: 6.4 (5.5) |
| Ji L 2016 | sita 100mg QD | 24 weeks | sita 100mg: 120; pbo: 127 | sita 100mg: 51.7 (10.2); pbo: 53.6 (9.7) | sita 100mg: 61.7; pbo: 68.5 | China | Chinese | sita 100mg: 1.1 (0.2); pbo: 1.1 (0.2) |
| Ji L 2017 | alo 12.5mg BID | 26 weeks | alo 12.5mg BID: 163; pbo: 163 | alo 12.5mg BID: 55.4 (9.62); pbo: 52.2 (10.17) | alo 12.5mg BID: 60.1; pbo: 58.3 | China, Malaysia, South Korea and Taiwan | alo 12.5mg BID: Asian 99.4, American Indian or Alaskan Native 0.6, Multiracial 0; pbo: Asian 98.8, American Indian or Alaskan Native 1.2, Multiracial 0 | not reported |
| Jung CH 2015 | evo 2.5mg QD; 5mg QD; 10mg QD | 12 weeks | evo 2.5mg QD: 40; 5mg QD: 43; 10mg QD: 37; pbo: 38 | evo 2.5mg QD: 52.10 (11.48); 5mg QD: 54.21 (9.74); 10mg QD: 53.16 (9.60); pbo: 54.42 (9.85) | evo 2.5mg QD: 55.00; 5mg QD: 58.14; 10mg QD: 54.05; pbo: 68.42 | South Korea | Korean | evo 2.5mg QD: 4.00 (3.57); 5mg QD: 3.98 (3.88); 10mg QD: 3.95 (3.88); pbo: 3.55 (2.85) |
| Kadowaki T 2013 | teneligliptin 10mg QD; teneligliptin 20mg QD + teneligliptin 40mg QD | 12 weeks | teneli 10: 84; teneli 20: 79; teneli 40: 81; pbo: 80 | teneli 10: 57.7 (9.1); teneli 20: 59.2 (9.5); teneli 40: 57.5 (10.4); pbo: 58.5 (9.6) | teneli 10: 59.5; teneli 20: 74.7; teneli 40: 65.4; pbo: 63.8 | Japan | Korean | teneli 10: 6.2 (5.2); teneli 20: 6.3 (6.4); teneli 40: 6.5 (6.1); pbo: 5.8 (5.0) |
| Kawamori R 2012 | linagliptin 5mg QD; linagliptin 10mg QD | 26 weeks | lina 5mg: 159; lina 10mg: 160; pbo: 80 | lina 5mg: 60.3 (9.4); lina 10mg: 61.3 (10.0); pbo: 59.7 (8.9) | lina 5mg: 69.8; lina 10mg: 70.0; pbo: 71.3 | Japan | Japanese | lina 5mg: ≤1 year 11.9, > 1-5 years 38.4, > 5 years 49.7; lina 10mg: ≤1 year 11.9, > 1-5 years 36.9, > 5 years 51.3; pbo: ≤1 year 8.8, > 1-5 years 45.0, > 5 years 46.3 |
| Kikuchi M 2009 | vilda 10mg BID; 25mg BID; 50mg BID | 12 weeks | vilda 10mg BID: n=71; 25mg BID: n=72; 50mg BID: n=76; pbo: 72 | vilda 10mg BID: 58.9 (8.6); 25mg BID: 57.8 (8.5); 50mg BID: 58.8 (8.6); pbo: 60.4 (8.1) | vilda 10mg BID: 73.2; 25mg BID: 63.9; 50mg BID: 67.1; pbo: 63.9 | Japan | Japanese | vilda 10mg BID: 4.5 (4.2); 25mg BID: 4.7 (4.5); 50mg BID: 4.7 (4.3); pbo: 7.1 (5.5) |
| Kumar PKM 2014 | saxa 5mg QD | 24 weeks | saxa 5mg: 107; pbo: 106 | not reported | not reported | India | Indian | not reported |
| Mohan V 2009 | sita100mg QD | 18 weeks | sita 100mg: 352; pbo: 178 | sita 100mg: 50.9 (9.3); pbo: 50.9 (9.3) | sita 100mg: 57; pbo: 60 | China, India, South Korea | sita 100mg: Chinese 46, Indian 36, Korean 18; pbo: Chinese 46, Indian 35, Korean 19 | sita 100mg: 2.1 (1.7); pbo: 1.9 (1.6) |
| Nonaka K 2008 | sita 100mg QD | 12 weeks | sita 100mg: 75; pbo: 76 | sita 100mg: 55.6 (8.6); pbo: 55.0 (8.0) | sita 100mg: 60; pbo: 66 | Japan | Japanese | sita 100mg: 4 (4.1); pbo: 4.1 (4.6) |
| Pan CY 2012 | saxa 5mg QD | 24 weeks | saxa 5mg: 284; pbo: 284 | saxa 5mg: 51.2 (10.0); pbo: 51.6 (10.3) | saxa 5mg: 56.3; pbo: 54.6 | China, India, Philippines, South Korea | [REGION] saxa 5mg: China 59.5, India 21.8, South Korea 6.3, Philippines 12.3; pbo: China 58.5, India 21.1, South Korea 7.7, Philippines 12.7 | saxa 5mg: 0.8 (1.4); pbo: 1.2 (2.6) |
| Pan CY 2017 | alo 25mg QD | 16 weeks | alo 25mg: 92; pbo: 92 | alo 25mg: 51.6 (10.4); pbo: 53.1 (8.9) | alo 25mg: 59.8; pbo: 58.1 | China, Taiwan, Hong Kong | [COUNTRY OR REGION] alo 25mg: China 97.8, Hong Kong 2.2, Taiwan 0; pbo: China 97.8, Hong Kong 1.1, Taiwan 1.1 | alo 25mg: 1.9 (2.4); pbo: 2.1 (2.8) |
| Park J 2017 | evo 5mg QD | 24 weeks | evo 5mg: 80; pbo: 80 | evo 5mg: 57.6 (11.0); pbo: 56.8 (9.8) | evo 5mg: 48.8; pbo: 57.5 | South Korea | Korean | evo 5mg: 4.74 (3.81); pbo: 4.25 (4.10) |
| Pi-Sunyer FX 2007 | vilda 50mg QD; vilda 50mg BID; vilda 100mg QD | 24 weeks | vilda 50mg QD: 88; 50mg BID: 83; 100mg QD: 91; pbo: 92 | vilda 50mg QD: 50.6 (10.4); 50mg BID: 50.2 (12.7); 100mg QD: 52.0 (11.7); pbo: 52.0 (12.0) | vilda 50mg QD: 55.7; 50mg BID: 56.6; 100mg QD: 53.8; pbo: 54.3 | USA, India, Slovakia | [RACE] vilda 50mg QD: Caucasian 54.5, Hispanic or Latino 18.2, Asian (Indian subcontinent) 15.9, Asian (non-Indian subcontinent) 3.4, Black 8.0; 50mg BID: Caucasian 53.0, Hispanic or Latino 21.7, Asian (Indian subcontinent) 18.0, Asian (non-Indian subcontinent) 1.2, Black 6.0; 100mg QD: Caucasian 58.2, Hispanic or Latino 12.1, Asian (Indian subcontinent) 16.5, Asian (non-Indian subcontinent) 1.1, Black 12.1; pbo: Caucasian 51.1, Hispanic or Latino 18.5, Asian (Indian subcontinent) 16.3, Asian (non-Indian subcontinent) 1.1, Black 13.0 | vilda 50mg QD: 1.8 (2.7); 50mg BID: 2.4 (3.2); 100mg QD: 2.1 (2.9); pbo: 2.5 (3.7) |
| Pratley RE 2006 | vilda 25mg BID | 12 weeks | vilda 25mg BID: 70; pbo: 28 | vilda 25mg BID: 56.9 (9.4); pbo: 52.8 (10.0) | vilda 25mg BID: 40; pbo: 60 | South America, Mexico | vilda 25mg BID: Black 2.9, Caucasian 47.1, Oriental 1.4, Other 48.6; pbo: Black 0, Caucasian 46.4, Oriental 0, Other 53.6 | vilda 25mg BID: 4.6 (5.6); pbo: 3.5 (5.7) |
| Pratley RE 2014 | alo 25mg QD; alo 12.5mg BID | 26 weeks | alo 25mg QD: 112; alo 12.5mg BID: 113; pbo: 109 | alo 25mg QD: 52.6 (9.38); alo 12.5mg BID: 53.7(9.7); pbo: 53.1 (9.60) | alo 25mg QD: 42.9; alo 12.5mg BID: 55.8; pbo: 50.5 | worldwide; not specified | alo 25mg QD: Asian 15.2, Black or African American 2.7, White 75.0, Other 7.1; alo 12.5mg BID: Asian 18.6, Black or African American 2.7, White 73.5, Other 5.3; pbo: Asian 18.3, Black or African American 7.3, White 69.7, Other 4.6 | alo 25mg QD: 3.6 (4.12); alo 12.5mg BID: 4.0 (4.80); pbo: 4.3 (4.78) |
| Raz I 2006 | sita 100mg QD; sita 200mg QD | 18 weeks | sita 100mg QD: 205; 200mg QD: 206; pbo: 110 | sita 100mg QD: 54.5 (10.0); 200mg QD: 55.4 (9.2); pbo: 55.5 (10.1) | sita 100mg QD: 53.7; 200mg QD: 50.5; pbo: 62.7 | Multinational, nations not specified | sita 100mg QD: White 69.3, Black 7.8, Hispanic 18.0, Asian 3.9, Other 1.0; 200mg QD: White 70.9, Black 5.3, Hispanic 18.9, Asian 3.4, Other 1.5; pbo: White 61.8, Black 10.9, Hispanic 20.0, Asian 4.5, Other 2.7 | sita 100mg QD: 4.5 (4.3); 200mg QD: 4.5 (3.9); pbo: 4.7 (5.0) |
| Rhee EJ 2010 | gemi 50mg QD; gemi 100mg QD; gemi 200mg QD | 12 weeks | gemi 50mg: 35; 100mg: 37; 200mg: 35; pbo: 34 | gemi 50mg: 52.43 (9.63); 100mg: 53.22 (11.92); 200mg: 54.29 (10.33); pbo: 51.26 (8.59) | gemi 50mg: 71.43; 100mg: 62.16; 200mg: 51.43; pbo: 67.65 | Korea | Korea | gemi 50mg: 4.53 (3.83); 100mg: 4.51 (5.22); 200mg: 3.18 (3.25); pbo: 4.31 (4.90) |
| Ristic S 2005 | vilda 25mg BID; vilda 25mg QD; vilda 50mg QD; vilda 100mg QD | 12 weeks | vilda 25mg BID: 51; 25mg QD: 54; 50mg QD: 53; 100mg QD: 63; pbo: 58 | vilda 25mg BID: 55.6 (10.9); 25mg QD: 57.4 (10.2); 50mg QD: 57.0 (10.2); 100mg QD: 56.2 (10.1); pbo: 54.6 (10.6) | vilda 25mg BID: 47.1; 25mg QD: 63.0; 50mg QD: 49.1; 100mg QD: 55.6; pbo: 56.9 | USA, Russia | vilda 25mg BID: Caucasian 80.4; 25mg QD: Caucasian 79.6; 50mg QD: Caucasian 77.4; 100mg QD: Caucasian 74.6, pbo: Caucasian 87.9 | vilda 25mg BID: 3.28 (3.81); 25mg QD: 3.10 (5.16); 50mg QD: 2.71 (3.24); 100mg QD: 3.03 (4.22); pbo: 2.28 (2.99) |
| Roden 2013 | empa 10mg QD; empa 25mg QD | 12 weeks | empa 10mg: 224; 25mg: 224; pbo: 228 | empa 10mg: 56.2 (11.6); 25mg: 53.8 (11.6); pbo: 54.9 (10.9) | empa 10mg: 63; 25mg: 65; pbo: 54 | Belgium, Canada, China, Germany, India, Ireland, Japan, Switzerland and USA | empa 10mg: Asian 64, White 34, Black/African-American 1, American-Indian/Alaska Native 0, Hawaiian/Pacific Islander 1, 25mg: Asian 64, White 33, Black/African-American 3, American-Indian/Alaska Native 0, Hawaiian/Pacific Islander 0; pbo: Asian 64, White 33, Black/African-American 3, American-Indian/Alaska Native 0, Hawaiian/Pacific Islander 0 | empa 10mg: ≤1 year: 39, >1-5 years: 41, >5-10 years: 13, >10 years: 7; 25mg: ≤1 year: 41, >1-5 years: 37, >5-10 years: 17, >10 years 6; pbo: ≤1 year: 32, >1-5 years: 46, >5-10 years: 15, >10 years: 8 |
| Rosenstock J 2008 | saxa 2.5mg QD; saxa 5mg QD; saxa 10mg QD; saxa 20mg QD; saxa 40mg QD  (100mg QD for 6 weeks) | 12 weeks | saxa 2.5mg QD: 55; 5mg QD: 47; 10mg QD: 63; 20mg QD: 54; 40mg QD: 52; 100mg QD: 44; pbo: 67 | saxa 2.5mg QD: 52.5 (10.53); 5mg QD: 53.7 (10.14); 10mg QD: 54.5 (8.61); 20mg QD: 53.6 (8.59); 40mg QD: 54.1 (11.12); 100mg QD: 51.4 (9.91); pbo: 52.8 (10.18) | saxa 2.5mg QD: 40; 5mg QD: 53; 10mg QD: 63; 20mg QD: 70; 40mg QD: 58; 100mg QD: 61; pbo: 63 | USA | saxa 2.5mg QD: Caucasian 85, African-American 11, Other 4; 5mg QD: Caucasian 87, African-American 13, Other 0; 10mg QD: Caucasian 84, African-American 8, Other 8; 20mg QD: Caucasian 87, African-American 7, Other 6; 40mg QD: Caucasian 92, African-American 4, Other 4; 100mg QD: Caucasian 77, African-American 16, Other 7; pbo: Caucasian 87, African-American 10, Other 3 | saxa 2.5mg QD: 1.0 (0.0-14.0); 5mg QD: 0.8 (0.0 to 8.2); 10mg QD: 0.7 (0.0 to 12.9); 20mg QD: 1.7 (0.0 to 13.0); 40mg QD: 1.3 (0.0 to 19.0); 100mg QD: 0.5 (0.0 to 26.0); pbo: 1.8 (0.0 to 23.0) |
| Rosenstock J 2009 A | saxa 2.5mg QD; saxa 5mg QD; saxa 10mg QD | 24 weeks | saxa 2.5mg QD: 102; 5mg QD: 106; 10mg QD: 98; pbo: 95 (did not include open label cohort) | saxa 2.5mg QD: 53.27 (10.06); 5mg QD: 53.91 (11.57); 10mg QD: 52.72 (11.27); pbo: 53.91 (12.32) | saxa 2.5mg QD: 56.9; 5mg QD: 50.9; 10mg QD: 45.9; pbo: 49.5 | USA | saxa 2.5mg QD: White 87.3, Black/African American 4.9, Asian 4.9, Other 2.9; 5mg QD: White 87.7, Black/African American 4.7, Asian 3.8, Other 3.8; 10mg QD: White 81.6, Black/African American 6.1, Asian 6.1, Other 6.1; pbo: White 83.2, Black/African American 6.3, Asian 3.2, Other 7.4 | saxa 2.5mg QD: 3.1 (3.5); 5mg QD: 2.5 (3.3); 10mg QD: 2.3 (3.1); pbo: 2.3 (2.7) |
| Scherbaum WA 2008 | vilda 50mg QD | 52 weeks | vilda 50mg QD: 156; pbo: 150 | vilda 50mg QD: 63.3 (10.2); pbo: 62.8 (11.0) | vilda 50mg QD: 59.6; pbo: 59.3 | Finland, France, Germany, Romania, Spain, Sweden | vilda 50mg QD: Caucasian 99.4, Other 0.6; pbo: Caucasian 99.3, Other 0.7 | vilda 50 mg QD: 2.5 (2.9); pbo: 2.7 (3.2) |
| Scott R 2007 | sita 5mg BID; sita 12.5mg BID; sita 25mg BID; sita 50mg BID | 12 weeks | sita 5mg BID: 125; 12.5mg BID: 123; 25mg BID: 123; 50mg BID: 124; pbo: 125 | sita 5mg BID: 55.1 (9.5); 12.5mg BID: 56.2 (9.0); 25mg BID: 55.6 (9.0); 50mg BID: 55.1 (9.8); pbo: 55.3 (9.7) | sita 5mg BID: 49.6; 12.5mg BID: 48.0; 25mg BID: 57.7; 50mg BID: 52.4; pbo: 62.4 | Multinational; nations not specified | sita 5mg BID: Asian 5.6, Black 6.4, Multi-racial 6.4, White 68.8, Other 12.8; 12.5mg BID: Asian 4.9, Black 4.9, Multi-racial 5.7, White 63.4, Other 21.1; 25mg BID: Asian 4.9, Black 8.9, Multi-racial 6.5, White 61.0, Other 18.7; 50mg BID: Asian 2.4, Black 4.8, Multi-racial 7.3, White 69.4, Other 16.1; pbo: Asian 2.4, Black 8.0, Multi-racial 7.2, White 66.4, Other 16.0 | sita 5mg BID: 4.3 (4.1); 12.5mg BID: 4.9 (5.0); 25mg BID: 5.0 (5.2); 50mg BID: 4.2 (4.0); pbo: 4.8 (4.7) |
| Seino Y  2011 | alo 6.25mg QD; alo 12.5mg QD; alo 25mg QD; alo 50mg QD | 12 weeks | alo 6.25mg: 79, 12.5mg: 84, 25mg: 80, 50mg: 79; pbo: 75 | alo 6.25mg: 58.1 (10.58), 12.5mg: 58.7 (9.54), 25mg: 59.5 (11.16), 50mg: 58.8 (9.23); pbo: 59.1 (10.47) | alo 6.25mg: 70.9, 12.5mg: 70.2, 25mg: 78.8, 50mg: 69.6; pbo: 74.7 | Japan | Japanese | alo 6.25mg: 6.63 (6.41), 12.5mg: 5.94 (5.32), 25mg: 6.98 (6.99), 50mg: 6.78 (5.95); pbo: 6.83 (6.07) |
| Sheu WHH 2015 | omari 0.25mg QW; omari 1mg  QW; omari 3mg QW; omari 10mg QW; omari 25mg QW | 12 weeks | omari 0.25mg once weekly: 113; 1mg once weekly: 115; 3mg once weekly: 114; 10mg once weekly: 115; 25mg once weekly: 114; pbo: 114 | omari 0.25mg once weekly: 54.3 (8.9); 1mg once weekly: 55.7 (8.5); 3mg once weekly: 55.3 (8.5); 10mg once weekly: 54.4 (10.0); 25mg once weekly: 55.1 (8.8); pbo: 55.9 (8.4) | omari 0.25mg once weekly: 57.5; 1mg once weekly: 58.3; 3mg once weekly: 57.0; 10mg once weekly: 48.7; 25mg once weekly: 60.5; pbo: 57.0 | 21 countries; not reported | omari 0.25mg once weekly: White 53.1, Asian 29.2, Multiracial 5.3, American Indian or Alaska Native 7.1, Black or African American 4.4, Native Hawaiian or other Pacific Islander 0.9; 1mg once weekly: White 60.9, Asian 28.7, Multiracial 3.5, American Indian or Alaska Native 5.2, Black or African American 1.7, Native Hawaiian or other Pacific Islander 0; 3mg once weekly: White 61.4, Asian 23.7, Multiracial 4.4, American Indian or Alaska Native 1.8, Black or African American 7.0, Native Hawaiian or other Pacific Islander 1.8; 10mg once weekly: White 55.7, Asian 26.1, Multiracial 7.0, American Indian or Alaska Native 7.8, Black or African American 2.6, Native Hawaiian or other Pacific Islander 0.9; 25mg once weekly: White 54.4, Asian 26.3, Multiracial 10.5, American Indian or Alaska Native 2.6, Black or African American 6.1, Native Hawaiian or other Pacific Islander 0; pbo: White 56.1, Asian 28.1, Multiracial 8.8, American Indian or Alaska Native 3.5, Black or African American 2.6, Native Hawaiian or other Pacific Islander 0.9 | omari 0.25mg once weekly: 4.8 (4.2); 1mg once weekly: 5.3 (4.3); 3mg once weekly: 5.4 (3.9); 10mg once weekly: 5.1 (4.6); 25mg once weekly: 5.9 (5.2); pbo: 5.8 (4.6) |
| Wu W 2015 | lina 5mg QD | 24 weeks | lina 5mg: 33; pbo: 22 | lina 5mg: 52.5 (11.0); pbo: 51.2 (7.5) | lina 5mg: 65.7; pbo: 50 | China | Chinese | not reported |
| Yang SJ 2013 | gemi 50mg QD | 24 weeks | gemi 50 mg: 87; pbo: 87 | gemi 50 mg: 54 (49-60); pbo: 52 (45 - 60) | gemi 50 mg: 57; pbo: 44 | Korea and India | gemi 50 mg: Indian 56 Korean 44; pbo: Indian 60, Korean 40 | gemi 50 mg: 3.24 (3.84); pbo: 2.86 (4.36) |
| Yang HK 2015 | ana 100mg BID; ana 200mg BID | 24 weeks | ana 100mg BID: 37; 200mg BID: 30; pbo: 38 | ana 100mg BID: 54.43 (9.86); 200mg BID: 57.70 (9.71); pbo: 56.74 (9.72) | ana 100mg BID: 40.54; 200mg BID: 60.00; pbo: 63.16 | Republic of Korea (South Korea) | Korean | ana 100mg BID: 3.17 (5.53); 200mg BID: 3.43 (3.40); pbo: 4.14 (4.10) |

**Characteristics of Included Studies – DPP4i Monotherapy continued**

| **Study ID** | **Baseline A1C % (SD)** | **Mean Change in HbA1C % vs. baseline** | **Mean Difference in HbA1c % vs pbo** | **HYPO def** | **Ascertainment of Hypo** | **Rescue Medication** | **Excl of Pts w events? At screening or during study)** |
| --- | --- | --- | --- | --- | --- | --- | --- |
| Agarwal P 2018 | teneli: 7.75 (n/a); pbo: 7.74 | [LSM] (SE) teneli: -0.304 (0.118); pbo: 0.251 (0.1565) | [LSM] (95% CI) teneli: -0.555 (0.176 to 0.934) | not reported | not reported | Metformin used as rescue medication. Data collected after initiation of rescue therapy were treated as missing. | no |
| Aschner P 2006 | sita 100mg: 8.0 (0.9); 200mg: 8.1 (0.9); pbo: 8.0 (0.8) | [LSM] sita100mg: -0.61 (-0.74 to -0.49); 200mg: -0.76 (-0.88 to -0.64); pbo: 0.18 (0.06 to 0.30) | [LSM] sita100mg: -0.79 (-0.96 to -0.62); 200mg: -0.94 (-1.11 to -0.77) | not reported | not reported | Rescue therapy (metformin) permitted. Sita 100mg included one episode after initiation of rescue. Data still included since same patient could have had other episodes not attributed to rescue therapy. | no |
| Barnett AH 2012 | lina 5mg: 8.1 (1.0); pbo: 8.1 (0.9) | lina 5mg: -0.39 (0.14); pbo: 0.21 (0.16) [FAS, LOCF, Model includes continuous baseline HbA1c, number of prior diabetes drugs, reason of metformin intolerance and treatment. | lina 5mg: -0.60 (-0.88 to -0.32) | Hypoglycaemia was defined according to American Diabetes Association guidelines as either asymptomatic with plasma glucose ≤3.9 mmol/l (≤70 mg/dl), symptomatic with plasma glucose ≤3.9 mmol/l (≤70 mg/dl) or severe (requiring third-party assistance to administer resuscitative action). | not reported | Rescue therapy was initiated with pioglitazone initially and, if blood glucose remained higher than the levels described above, insulin was introduced with or without continuation of pioglitazone. | no |
| Barzilai N 2011 | sita: 7.8 (0.8); pbo: 7.8 (0.7) | [LSM] sita: -0.5 (-0.7 to -0.2); pbo: 0.2 (0.0 to 0.5) | [LSM] sita: -0.7 (-0.9 to -0.5) | not reported | All randomized patients were asked to measure and record self-monitored blood glucose (SMBG) data at four specific times on each of the following 3 days: ‘day 2’ ‘day 3’ and ‘day 7’. Times for SMBG were (1) immediately before breakfast (after fasting for at least 8 hours), (2) 2 hours after breakfast, (3) immediately before the evening meal, and (4) 2 hours after the evening meal. | not reported | no |
| Chen Y 2015 | lina 5mg: 7.95 (0.89); pbo: 8.09 (0.91) | lina 5mg: -0.68 (0.07); pbo: -0.18 (0.10) (adjusted mean (SE)) | lina 5mg: -0.50 (0.11) (adjusted mean difference (SE)) | Hypoglycemia was classified by investigators as asymptomatic  with glucose concentration ≤70 mg/dL, documented symptomatic  with glucose concentration of 54–70 mg/dL, documented  symptomatic with glucose concentration <54 mg/dL but no  need for external assistance, and severe hypoglycemia requiring the assistance of another person. | not reported; investigator-defined | The use of metformin as rescue medication was permitted. | no |
| DeFronzo 2008 | 7.9 (0.08) | [LSM] alo 12.5: -0.56 (p<0.001); alo 25: -0.59 (p<0.001); pbo: -0.02 | [LSM] manually calculated alo 12.5: -0.54; alo 25: -0.57 | not reported | not reported | Rescue medication permitted but type not known. | no |
| Dejager S 2007 | vilda 50mg QD: 8.2 (0.8); 50mg BID: 8.6 (0.8); 100mg QD: 8.4 (0.8); pbo: 8.4 (0.8) | vilda 50mg QD: -0.8 (0.1); 50mg BID: -0.8 (0.1); 100mg QD: -0.9 (0.1); pbo: -0.3 (0.1) | vilda 50mg QD: -0.5 (0.2); 50mg BID: -0.5 (0.2); 100mg QD: -0.6 (0.2) | Hypoglycemia was defined as symptoms suggestive of low blood glucose confirmed by self-monitored blood glucose (SMBG) measurement < 3.1 mmol/l plasma glucose equivalent. | Confirmed by SMBG measurement < 3.1 mmol / l plasma glucose equivalent | not reported | no; but a patient could also be discontinued due to UTE [unsatisfactory therapetuci effect] |
| Del Prato S  2011 | lina 5mg QD: 8.00; pbo: 8.00 | lina 5mg QD: -0.44; pbo: 0.25 | -0.69 (-0.85 to -0.53) | not reported | not reported | Rescue medication (metformin) permitted. | no |
| Gantz I 2017 | sita: 8.0 (0.8); pbo: 8.1 (0.7) | [LSM] sita: -0.65 (-0.74 to -0.55); pbo: 0.13 (-0.0 to 0.27) based on an LDA model with terms for treatment, prior AHA therapy status (yes/no), time, and interaction of time by treatment, time by prior AHA therapy status, and time by treatment by prior AHA therapy status, with the constraint that the mean baseline was the same for all treatment groups). s.e., standard error | [LSM] sita: -0.78 (-0.94 to -0.61) | Symptomatic hypoglycaemia: episode with clinical symptoms attributed to hypoglycaemia, without regard to glucose level | not reported | not reported | no |
| Gantz I 2017 | omarigliptin: 7.9 (0.7); pbo: 8.1 (0.7) | [LSM] omari: -0.66 (-0.76 to -0.57); pbo: 0.13 (-0.0 to 0.27) based on an LDA model with terms for treatment, prior AHA therapy status (yes/no), time, and interaction of time by treatment, time by prior AHA therapy status, and time by treatment by prior AHA therapy status, with the constraint that the mean baseline was the same for all treatment groups). s.e., standard error | [LSM] omari: -0.80 (-0.96 to -0.63) | Symptomatic hypoglycaemia: episode with clinical symptoms attributed to hypoglycaemia, without regard to glucose level | not reported | not reported | no |
| Goldstein BJ 2007 | sita 100mg: 8.9 (1.0); pbo: 8.7 (1.0) | [LSM] sita 100mg: -0.66 (-0.83 to -0.50); pbo: 0.17 (0.0 to -0.33) | [LSM] sita 100mg: -0.83 (-1.06 to -0.60) | not reported | not reported | Glycemic rescue therapy (glyburide [glibenclamide]) permitted. Safety and efficacy endpoints excluded data after rescue with glyburide. | no |
| Haak T 2012 | lina 5mg: 8.7 (1.0); pbo: 8.7 (1.0) | adjusted mean (SE) lina 5mg: -0.5 (0.1); pbo: 0.1 (0.1) | adjusted mean (SE) lina 5mg: -0.6 (0.1) | Severe hypoglycemia defined as requiring the assistance of another person to actively administer carbohydrate, glucagon or other. | Hypoglycemic episodes were recorded and analyzed  separately from other AEs. Hypoglycemic event intensity  was graded according to the investigator’s discretion | Rescue therapy (with sulphonylureas, thiazolidinediones or insulin) was permitted. Values obtained after rescue medication was initiated were not used in the LOCF. | unlikely, but hypoglycemic events graded at investigators discretion; criteria not specified and could lead to patient exclusion |
| Hanefeld M 2007 | sita 25mg QD: 7.7 (0.9); 50mg QD: 7.6 (1.0); 100mg QD: 7.8 (0.9); 50mg BID: 7.8 (0.9); pbo: 7.6 (0.9) | sita 25mg QD: -0.28 (-0.42 to -0.14); 50mg QD: –0.44 (–0.58 to –0.30); 100mg QD: –0.44 (–0.58 to –0.30); 50mg BID: –0.43 (–0.56 to –0.29); pbo: 0.12 (-0.02 to 0.26) | sita 25mg QD: -0.39 (-0.59 to -0.20); 50mg QD: -0.55 (-0.75 to -0.36); 100mg QD: -0.56 (-0.75 to -0.36); 50mg BID: -0.54 (-0.74 to -0.35) | The determination of hypoglycemia was made by the study site investigator based upon information provided by the patient in a logbook.  A fingerstick blood glucose determination concurrent with the episode was not required to assess an episode as hypoglycemia, although investigators could include the fingerstick glucose measurement, if it was available, in their assessment of the episode. Pt regularly measured blood glucose via. fingerstick 7x daily. | Confirmed by investigator via. patient logbook (subjective hypoglycemia + fingerstick glucose levels if measure + debrief with patient) | not reported | no |
| Home P 2018 | omari 25mg QW: 8.0 (0.9); pbo: 8.1 (1.0) | [LSM] omari 25mg QW: -0.49 (-0.73 to -0.24); pbo: -0.10 (-0.34 to 0.14) | [LSM] omari 25mg QW: -0.39 (-0.59 to -0.19) | Symptomatic hypoglycemia: episode with clinical symptoms attributed to hypoglycemia, without regard to glucose level.  Severe hypoglycemia: episode that required assistance, either medical or non-medical.  Asymptomatic hypoglycemia: self-measured glucose values ≤3.9 mmol/L without symptoms | A questionnaire  was provided to participants to collect data on  hypoglycemia. | Rescued by adding open-label metformin and after by adding open-label glimepiride. | no |
| Hong S 2016 | teneli 20mg: 7.74 (0.61); pbo: 7.74 (0.53) | [LSM] (SE) teneli 20mg: -0.90 (0.09); pbo: 0.03 (0.12) | [LSM] teneli 20mg: -0.94 (-1.22 to -0.65) | Review of patient data for signs and symptoms of hypoglycemia in addition to reviewing self-monitoring of blood glucose data | The incidence of hypoglycemia was assessed by reviewing patient data for signs and symptoms of hypoglycemia in addition to reviewing self-monitoring of blood glucose (SMBG) data. | Rescue medication not specified. | no |
| Inagaki N 2014 | trela 12.5mg QW: 8.18 (0.89); 25mg QW: 7.99 (0.77); 50mg QW: 8.07 (0.86); 100mg QW: 8.41 (0.97); 200mg QW: 7.84 (0.76); pbo: 8.15 (0.95) | [LSM] (SE) trela 12.5mg QW: -0.37 (0.068); 25mg QW: -0.32 (0.070); 50mg QW: -0.42 (0.070); 100mg QW: -0.54 (0.068) 200mg QW: -0.55 (0.069); pbo: 0.35 (0.068) | [LSM] manually calculated trela 12.5mg QW: -0.72; 25mg QW: -0.67; 50mg QW: -0.77; 100mg QW: -0.89; 200mg QW: -0.90 | not reported. Investigator-defined, criteria not specified | not reported | Rescue not permitted. Patients requiring rescue medication were withdrawn from study. | no |
| Inagaki et al. 2015 | alo 25mg: 7.87 (0.86); pbo: 7.72 (0.77) | [LSM] alo 25mg: -0.46 (0.63); pbo: 0.24 (0.52) | [LSM] alo 25mg: -0.70 (-0.905 to -0.493) | not reported. Investigator-defined, criteria not specified | A data  safety monitoring board was not set up in this study. | not reported. | no |
| Inagaki et al. 2015 | trela 100mg QW: 7.73 (0.85); pbo: 7.72 (0.77) | [LSM] trela 100mg QW: -0.32 (0.59); pbo: 0.24 (0.52) | [LSM] trela 100mg QW: -0.56 (-0.754 to -0.368) | not reported. Investigator-defined, criteria not specified | A data  safety monitoring board was not set up in this study. | not reported. | no |
| Iwamoto Y 2010 | sita 25mg QD: 7.49 (0.82); 50mg QD: 7.57 (0.84); 100mg QD: 7.56 (0.80); 200mg QD: 7.65 (0.82); pbo: 7.74 (0.93) | [LSM] sita 25mg QD: -0.41 (-0.52 to -0.29); 50mg QD: -0.71 (-0.83 to -0.59); 100mg QD: -0.69 (-0.81 to -0.56); 200mg QD: -0.76 (-0.89 to -0.64); pbo: 0.28 (0.16 to 0.40) | [LSM] sita 25mg QD: -0.69 (-0.85 to -0.52); 50mg QD: -0.99 (-1.16 to -0.82); 100mg QD: -0.96 (-1.14 to -0.79); 200mg QD: -1.04 (-1.21 to -0.86) | Patients were counseled to record results of self-monitored blood glucose levels and symptoms of hypoglycemia (e.g., sweating, anxiety, palpitations, headache, blurred vision, clouding of consciousness in diaries) for proper assessment of hypoglycemic events during the study. | Hypoglycemia was assessed by the study site investigators through reviewing patient self-reports of signs and symptoms of hypoglycemia. A fingerstick blood glucose determination concurrent with the episode was not required to assess an episode as hypoglycemia, although investigators could include the fingerstick glucose measurement, if it was available, in their assessment of the episode. | not reported | no |
| Ji L 2016 | sita 100mg: 8.7 (1.1); pbo: 9.0 (1.1) | [LSM] sita 100mg: -0.99 (-1.24 to -0.75); pbo: -0.59 (-0.84 to -0.34) | [LSM] sita 100mg: -0.40 (-0.71 to -0.09) (p=0.011) | Any episode with symptoms consistent with hypoglycemia (e.g., weakness, dizziness, shakiness, increased sweating, palpitations or confusion) was reported as an episode of symptomatic hypoglycemia without a requirement for confirmatory blood glucose values. Asymptomatic hypoglycemia was defined as an episode without symptoms of hypoglycemia, but with fingerstick glucose level ≤3.9 mmol/L (≤70 mg/dL). Severe hypoglycemia was defined as any episode requiring assistance, either medical or non-medical. Episodes with a markedly depressed level of consciousness, loss of consciousness or seizure were to be classified as having required medical assistance, whether or not medical assistance was obtained. | not reported | Rescue therapy with open-label glipizide. The primary approach to analyzing safety data treated data obtained after the initiation of rescue therapy as missing; a secondary approach included all data, regardless of rescue therapy. | no |
| Ji L 2017 | alo 12.5mg BID: 8.48 (0.71); pbo: 8.21 (0.77) | [LSM] (SE) alo 12.5mg BID: -0.86 (0.11); pbo: -0.19 | [LSM] alo 12.5mg BID: -0.68 (-0.889 to -0.467) | not reported | not reported | Rescue confounding. Hyperglycemic rescue permitted. | no |
| Jung CH 2015 | evo 2.5mg QD: 7.67 (0.83); 5mg QD: 7.58 (0.71); 10mg QD: 7.71 (0.88); pbo: 7.57 (0.87) | [LSM] evo 2.5mg QD: -0.59 (0.10); 5mg QD: -0.70 (0.10); 10mg QD: -0.64 (0.11) | [LSM] evo 2.5mg QD: -0.47 (-0.80 to -0.15); 5mg QD: -0.57 (-0.86 to -0.29); 10mg QD: -0.53 (-0.83 to -0.23) | not reported | not reported | If rescue therapy required, patients were dropped from the study. | no |
| Kadowaki T 2013 | teneli 10: 7.9 (0.7); teneli 20: 7.8 (0.7); teneli 40: 7.7 (0.7); pbo: 8.0 (0.7) | [LSM] teneli 10: -0.8 (0.1); teneli 20: -0.8 (0.1); teneli 40: -0.9 (0.1); pbo: 0.1 (0.1) | [LSM] teneli 10: -0.9 (-1.0 to -0.7); teneli 20: -0.9 (-1.1 to -0.7); teneli 40: -1.0 (-1.2 to -0.9) | not reported | Patients were provided a glucose  meter and were given diaries in which to record hypoglycemic  symptoms and self-monitored blood glucose (SMBG) concentrations.  Hypoglycaemia was assessed by the investigators who reviewed the patient’s self-reported signs/symptoms of  hypoglycaemia and SMBG data. | not reported | no |
| Kawamori R 2012 | lina 5mg: 8.07 (0.66); lina 10mg: 7.98 (0.68); pbo: 7.95 (0.67) | lina 5mg: -0.24 (0.06); lina 10mg: -0.25 (0.06); pbo: 0.63 (0.08) | lina 5mg: -0.87 (-1.04, -0.70); lina 10mg: -0.88 (-1.05, -0.71) | Hypoglycaemia was defined according to American Diabetes Association guidelines | not reported | not reported | no |
| Kikuchi M 2009 | vilda 10mg BID: 7.40 (0.8); 25mg BID: 7.40 (0.9); 50mg BID: 7.40 (0.8); pbo: 7.4 (0.8) | [LSM] vilda 10mg BID: -0.53; 25mg BID: -0.67; 50mg BID: -0.92; pbo: 0.28 (SE n/a) | [LSM] (SE) vilda 10mg BID: -0.8 (0.10); 25mg BID: -1.0 (0.10); 50mg BID: -1.2 (0.10) | Symptoms suggestive of hypoglycemia were classified as a hypoglycemic event if reversed by therapeutic actions (intake of sucrose, etc.) or as an AE, if not reversed. The severity of hypoglycemic events was graded on a scale of 2. Grade 1 was defined as signs or symptoms suggestive of hypoglycemia that could be managed by the patient either with sucrose or in any other appropriate way. Grade 2 was defined as any hypoglycemic episode wherein the patient required assistance of others or hospitalization. Plasma glucose was not measured in any patient during the occurrence of hypoglycemic events. | none | not reported | no |
| Kumar PKM 2014 | not reported, inclusion criteria were HbA1c between 7-10% | saxa 5mg: -0.51; pbo: -0.05 (SE n/a) | saxa 5mg: -0.46 (0.14) (-0.73 to -0.18) (p=0.0011) adjusted mean absolute reductions (SE) | not reported | not reported | not reported | no |
| Mohan V 2009 | sita 100mg: 8.7 (1.0); pbo: 8.8 (1.1) | [LSM] sita 100mg: -0.7 (-0.8 to -0.6); pbo: 0.3 (0.1 to 0.5) | [LSM] sita 100mg: -1.0 (-1.2 to -0.8) | not reported | not reported | not reported | no |
| Nonaka K 2008 | sita 100mg: 7.5 (0.9); pbo: 7.7 (0.9) | [LSM] sita 100mg: -0.65 (-0.80 to -0.50); pbo: 0.41 (0.26 to 0.56) | [LSM] sita 100mg: -1.05 (-1.27 to -0.84) | Symptoms of hypoglycemia include sweating, anxiety, palpitations, headache, blurred vision, clouding of consciousness. Severe hypoglycemia not specified. | Patients were given diaries on which to record study drug intake, results of twice a-  week home self-monitoring of blood glucose levels (SMBG),  symptoms of hypoglycemia, and other symptoms. In the event  of symptoms of hypoglycemia (e.g., sweating, anxiety,  palpitations, headache, blurred vision, clouding of consciousness), patients were to obtain a SMBG, take countermeasures (e.g., ingest glucose), and notify the investigator. Patients were  to notify the investigator if SMBG showed glucose levels <60 or >270 mg/dL. | not reported | no |
| Pan CY 2012 | saxa 5mg: 8.1 (0.8); pbo: 8.2 (0.8) | saxa 5mg: -0.84; pbo: -0.34 | saxa 5mg: -0.50 (-0.65 to -0.34) | Reported hypoglycaemia was defined as signs and symptoms consistent with hypoglycaemia with or without a documented glucose measurement. Confirmed hypoglycemic events were defined as those associated with symptoms of hypoglycaemia and a documented plasma glucose level of ≤2.8 mmol/L. | none; no episodes of confirmed hypoglycaemia (symptoms and plasma glucose level ≤2.8 mmol/  L) | Rescue therapy with open-label metformin was permitted. Data on or after rescue therapy excluded from study. | no |
| Pan CY 2017 | alo 25mg: 8.04 (0.92); pbo: 7.86 (0.78) | [LSM] alo 25mg: -0.99; pbo: -0.42 (SE n/a) | [LSM] alo 25mg: -0.58 (-0.78 to -0.37) | Mild-to-moderate hypoglycemia, whether symptomatic or asymptomatic, was defined as plasma glucose levels <3.9mmol/L. Severe hypoglycemia was defined as any hypoglycemic episode that required the assistance of another person to actively administer carbohydrate, glucagon, or other resuscitative actions, and was associated with a documented plasma glucose level <3.9 mmol/L. | not reported | Rescue therapy permitted. | no |
| Park J 2017 | evo 5mg: 7.21 (0.56); pbo: 7.20 (0.63) | evo 5mg: -0.23; pbo: 0.05 | evo 5mg: -0.28 (p<0.001) | Hypoglycaemia was defined as signs and/or symptoms consistent with hypoglycaemia with or without a documented glucose measurement, or as plasma glucose level ≤3.9 mmol/L without signs or symptoms. | not reported | not reported | no |
| Pi-Sunyer FX 2007 | vilda 50mg QD: 8.4 (0.9); 50mg BID: 8.4 (0.9); 100mg QD: 8.3 (0.8); pbo: 8.5 (0.8) | average mean change (SD) vilda 50mg QD: -0.5 (0.1); 50mg BID: -0.7 (0.1); 100mg QD: -0.8 (0.1); pbo: 0.0 (0.1) | vilda 50mg QD: 0.011; 50mg BID: <0.001; 100mg QD: <0.001 | Patients were provided with glucose monitoring devices and supplies and instructed on their use. Confirmed hypoglycemia was defined as symptoms suggestive of low blood glucose confirmed by self-monitored blood glucose (SMBG) measurement <3.1 mmol/L plasma glucose equivalent. Instances of SMBG measurement <3.1 mmol/L plasma glucose equivalent without accompanying symptoms were recorded as asymptomatic low blood glucose. Severe hypoglycemia was defined as any episode requiring the assistance of another party. | Patient reported; with or without confirmed blood glucose measurement | not reported | no |
| Pratley RE 2006 | vilda 25mg BID: 8.0 (0.9); pbo: 8.1 (1.2) | vilda 25mg BID: -0.6 (0.1); pbo: 0.0 (0.2) | vilda 25mg BID: -0.6 (0.2) (p=0.0012) | Plasma glucose less than or equal to 3.1 mmol/l | Subjects were provided with glucose monitoring devices and supplies and instructed on their use. An episode of hypoglycemia was defined as symptoms consistent with hypoglycemia accompanied by a glucose measurement less than or equal to 3.1 mmol/l. | not reported | no |
| Pratley RE 2014 | not reported; The majority of patients (60% overall) entered with a baseline  HbA1c of 8.5% or lower. | [LSM] (SE) alo 25mg QD: -0.56 (0.093); alo 12.5mg BID: -0.65 (0.094); pbo 0.15 (nss) | [LSM] (SE) manually calculated: alo 25mg QD: -0.72; alo 12.5mg BID: -0.80 | Mild to moderate hypoglycaemia (blood glucose <70 mg/dl (3.89 mmol/L), symptomatic or asymptomatic). All hypoglycemic episodes were associated with a blood glucose <70 mg/dl (3.89 mmol/l). Severe episodes required assistance. | Use of a home glucose  monitor and diary to record hypoglycemic episodes. | Hyperglycemic rescue (SU or other) was permitted. If a sulfonylurea was contraindicated or inappropriate, other rescue medications were prescribed at the investigator’s discretion; these patients discontinued study drug but continued with visits and procedures. | no |
| Raz I 2006 | sita 100mg QD: 8.0 (0.8); 200mg QD: 8.1 (0.9); pbo: 8.0 (0.9) | [LSM] sita 100mg QD: -0.48 (-0.61 to -0.35); 200mg QD: -0.36 (-0.48 to -0.23); pbo: 0.12 (-0.05 to 0.30) | [LSM] sita 100mg QD: -0.60 (-0.82 to -0.39); 200mg QD: -0.48 (-0.70 to -0.26) | not reported | not reported | Rescue therapy (metformin) permitted. Data collected prior to rescue were included in the efficacy analyses. | no |
| Rhee EJ 2010 | gemi 50mg: 8.24 (1); 100mg: 8.18 (1.09); 200mg: 8.16 (1.13); pbo; 8.2 (1.21) | gemi 50mg: -0.98 (0.76); 100mg: -0.74 (0.79); 200mg: -0.78 (0.78); pbo: -0.06 (0.76) | gemi 50mg: -0.92 (0.76); 100mg: -0.68 (0.78); 200mg; -0.72 (0.77) | not reported | no | not reported | no |
| Ristic S 2005 | vilda 25mg BID: 7.64 (0.69); 25mb QD: 7.73 (0.80); 50mg QD: 7.70 (0.82); 100mg QD: 7.64 (0.75); pbo: 7.76 (0.83) | [LSM] (SE): 25mg BID: -0.31 (0.11); 25mg QD: -0.27 (0.10); 50mg QD: -0.56 (0.10); 100mg QD: -0.53 (0.10); pbo: -0.13 (0.10) | [LSM] (SE): 25mg BID: -0.18 (0.15); 25mb QD: -0.14 (0.14); 50mg QD: -0.43 (0.14); 100mg QD: -0.40 (0.14) | All events (symptomatic or asymptomatic) determined by the investigator as hypoglycaemia were included in the adverse event summary. There were only two cases of symptomatic confirmed hypoglycaemia (plasma glucose 3.7 mmol/l). | Determined by investigator | not reported | no |
| Roden 2013 | empa 10mg: 7.87 (0.88); 25mg: 7.86 (0.85); pbo: 7.91 (0.78) | empa 10mg: -0.66 (-0.76 to -0.56); 25mg: -0.78 (-0.88 to -0.67); pbo: 0.08 (-0.03 to 0.18) | empa 10mg: -0.74 (-0.88 to -0.59); 25mg: -0.85 (-0.99 to -0.71) | Confirmed hypoglycemic adverse events (hypoglycemic episodes reported as adverse events when plasma glucose was <3·9 mmol/L, required assistance, or both) | Confirmed hypoglycemia either plasma glucose <3·9 mmol/L or requiring assistance, or both | Rescue medication permitted. The initiation, choice, and dose of rescue medication used were at the discretion of the investigator. | no |
| Rosenstock J 2008 | saxa 2.5mg QD: 7.7 (0.97); 5mg QD: 7.9 (1.09); 10mg QD: 8.0 (1.14); 20mg QD: 7.9 (0.99); 40mg QD: 7.8 (1.00); 100mg QD: 7.8 (1.01); pbo: 8.0 (0.98) | (SE) [95% CI]: saxa 2.5mg QD: -0.72 (0.12) (-0.97 to -0.48); 5mg QD: -0.90 (0.14) (-1.17 to -0.63); 10mg QD: -0.81 (0.11) (-1.03 to -0.58); 20mg QD: -0.74 (0.12) (-0.98 to -0.50); 40mg QD: -0.80 (0.12) (-1.04 to -0.56); 100mg QD: n/a; pbo: 0.27 (0.11) (-0.49 to -0.05) | (SE) [95% CI]: saxa 2.5mg QD: -0.45 (0.17) (-0.78 to -0.13); 5mg QD: -0.63 (0.18) [-0.97 to -0.29] ; 10mg QD: -0.54 (0.16) [-0.85 to -0.23]; 20mg QD: -0.47 (0.17) (-0.80 to -0.14); 40mg QD: -0.53 (0.17) (-0.86 to -0.20) | Confirmed hypoglycaemia was defined as a finger-stick blood glucose value of ≤50 mg/dl (2.78 mmol/L) associated with classical symptoms. Severe hypoglycemia defined as requiring the assistance of another person to actively administer carbohydrate, glucagon or other. | Confirmed hypoglycaemia, defined as a finger-stick blood glucose value 50 mg/dl. | Rescue medication permitted. | no |
| Rosenstock J 2009 A | saxa 2.5mg QD: 7.9 (0.9); 5mg QD: 8.0 (1.1); 10mg QD: 7.8 (0.9); pbo: 7.9 (0.9) | (SE) saxa 2.5mg QD: -0.43; 5mg QD: -0.46; 10mg QD: -0.54; pbo: 0.19 | manually calculated saxa 2.5mg QD: -0.24; 5mg QD: -0.27; 10mg QD: -0.35 | Symptoms of hypoglycemia and confirmed hypoglycemia, defined as symptoms of hypoglycemia with a fingerstick glucose ≤50 mg/dL (2.8 mmol/L), were also recorded. | Reported by pt; no cases confirmed with fingerstick | Open-label metformin as rescue therapy. Efficacy and safety measurements obtained after rescue were not included  in analyses. | no |
| Scherbaum WA 2008 | vilda 50mg QD: 6.7 (0.4); pbo: 6.8 (0.4) | (adjusted mean change) vilda 50mg QD: -0.2 (0.1) (p<0.001); pbo: 0.1 (0.1) | vilda 50mg QD: -0.3 (0.1) (p<0.001) adjusted mean change | Hypoglycaemia was defined as symptoms suggestive of low blood glucose confirmed by self-monitored blood glucose measurement <3.1 mmol/l plasma glucose equivalent. Severe hypoglycaemia was defined as any episode requiring the assistance of another party. | Self-confirmed with blood glucose measurement | not reported | No, not specifically. But patients with diabetic complications or lab abnormalities within the last 6 months were excluded. |
| Scott R 2007 | sita 5mg BID: 7.9 (1.0); 12.5mg BID: 7.9 (0.9); 25mg BID: 7.9 (0.9); 50mg BID: 7.8 (1.0); pbo: 7.9 (1.0) | [LSM] sita 5mg BID: -0.15 (-0.29 to -0.01); 12.5mg BID: -0.41 (-0.55 to -0.27); 25mg BID: -0.43 (-0.56 to -0.29); 50mg BID: -0.54 (-0.68 to -0.40); pbo: 0.23 (0.10 to 0.37) | [LSM] sita 5mg BID: -0.38 (-0.58 to -0.19); 12.5mg BID: -0.64 (-0.84 to -0.45); 25mg BID: -0.66 (-0.85 to -0.47); 50mg BID: -0.77 (-0.96 to -0.58) | not reported | Hypoglycaemia was assessed by study site investigators through reviewing  daily glucose logs and patient self-report of signs and symptoms of hypoglycaemia. | not reported | No, but they did exclude patients with hypoglycemia from glipizide arm of study (data not recorded here). |
| Seino Y  2011 | alo 6.25mg: 7.85 (0.94), 12.5mg: 7.99 (0.88), 25mg: 7.88 (0.99), 50mg: 8.01 (0.97); pbo: 7.85 (0.89) | alo 6.25mg: -0.51 (0.68), 12.5mg: -0.70 (0.57), 25mg: -0.76 (0.55), 50mg: -0.82 (0.47); pbo: 0.06 (0.46) | alo 6.25mg: -0.57, 12.5mg: -0.76, 25mg: -0.82, 50mg: -0.88 manually calculated | not reported | not reported | not reported | Yes, one patient in the extension experienced severe hypo and was withdrawn from the study. |
| Sheu WHH 2015 | omari 0.25mg once weekly: 8.1 (0.9); 1mg once weekly: 8.0 (0.9); 3mg once weekly: 7.9 (0.9); 10mg once weekly: 8.0 (0.9); 25mg once weekly: 8.1 (1.0); pbo: 8.1 (0.9) | [LSM] omari 0.25mg once weekly: -0.14 (-0.30 to 0.01); 1mg once weekly: -0.36 (-0.51 to -0.20); 3mg once weekly: -0.35 (-0.50 to -0.19); 10mg once weekly: -0.53 (-0.68 to -0.37); 25mg once weekly: -0.57 (-0.73 to -0.42); pbo: 0.14 (-0.01 to 0.30) | [LSM] omari 0.25mg once weekly: -0.28 (-0.50 to -0.06); 1mg once weekly: -0.50 (-0.71 to -0.28); 3mg once weekly: -0.49 (-0.70 to -0.27); 10mg once weekly: -0.67 (-0.88 to -0.45); 25mg once weekly: -0.72 (-0.93 to -0.50) | not reported | not reported | Rescued with open-label metformin. Data acquired after the initiation of rescue therapy were treated as missing. | no |
| Wu W 2015 | lina 5mg: 7.97 (0.68); pbo: 8.00 (0.69) | [sd not reported] lina 5mg; -1.2 (0.7); pbo: -0.4 (0.4) | manually calculated: -0.8 (n/a) | Hypoglycemia was defined according to American Diabetes Association guidelines (Seaquist et al. 2013) | Self-reported capillary blood glucose levels of 3.5 mmol/L, 3.7 mmol/L, and 3.7 mmol/L. | not reported | no |
| Yang SJ 2013 | gemi 50 mg: 8.2 (1.0); pbo: 8.3 (1.1) | [LSM] not reported | [LSM] gemi 50 mg: -0.71 (-1.04 to -0.37) | not reported | no | Rescue therapy with metformin permitted. | no |
| Yang HK 2015 | ana 100mg BID: 7.13 (0.72); 200mg BID: 7.19 (0.73); pbo: 7.11 (0.63) | ana 100mg BID: -0.50 (0.45); 200mg BID: -0.51 (0.55); pbo: 0.23 (0.62) | ana 100mg BID: -0.73; 200mg BID: -0.74 (CI (n/a) | not reported | not reported | not reported | no |

**Characteristics of Included Studies – GLP1RA Monotherapy**

| **Study ID** | **Dose** | **Study Duration** | **n=** | **Mean Age (SD)** | **Gender (% male)** | **Countries Studied** | **Ethnicity (%)** | **Duration of Diabetes in Years (SD)** |
| --- | --- | --- | --- | --- | --- | --- | --- | --- |
| Fonseca VA 2012 | lixi 20μg QD | 12 weeks | lixi 2-step dose increase: 120; 1-step dose increase: 119; pbo: 122 | lixi 2-step dose increase: 53.3 (9.7); 1-step dose increase: 53.8 (10.9); pbo: 54.1 (11.0) | lixi 2-step dose increase: 52.5; 1-step dose increase: 52.9; pbo: 49.2 | Belgium, India, Israel, Japan, South Korea, Mexico, Poland, Romania, Russian Federation, Tunisia, Ukraine, USA | lixi 2-step dose increase: Caucasian 73.3, Asian 22.5, Black 0, Other 4.2; 1-step dose increase: Caucasian 71.4, Asian 24.4, Black 2.5, Other 1.7; pbo: Caucasian 73.8, Asian 19.7, Black 2.5, Other 4.1 | [MEDIAN DURATION w range] 2-step dose increase: 1.4 (0.2-21.5); 1-step dose increase: 1.1 (0.2-23.9); pbo: 1.4 (0.2-12.5) |
| Grunberger G 2012 | dula 0.1mg QW; dula 0.5mg QW; dula 1.0mg QW; dula 1.5mg QW | 12 weeks | dula 0.1mg: 35; 0.5mg: 34; 1.0mg: 34; 1.5mg: 29; pbo: 32 | dula 0.1mg QW: 56.3 (9.2); 0.5mg: 56.9 (9.1); 1.0mg: 57.2 (8.8); 1.5mg: 57.5 (7.9); pbo: 55.0 (9.3) | dula 0.1mg QW: 31.4; 0.5mg: 47.1; 1.0mg: 47.1; 1.5mg: 44.8; pbo:56.3 | 7 countries; not specified | dula 0.1mg QW: Caucasian 83, Asian 11, Black or African-American 3, Others 3; 0.5mg: Caucasian 82, Asian 15, Black or African-American 3, Others 0; 1.0mg: Caucasian 77, Asian 15, Black or African-American 0, Others 9; 1.5mg: Caucasian 83, Asian 14, Black or African-American 3, Others 0; pbo: Caucasian 78, Asian 16, Black or African-American 3, Others 3 | dula 0.1mg QW: 3.9 (3.2); 0.5mg: 3.7 (3.8); 1.0mg: 3.3 (2.5); 1.5mg: 4.6 (4.1); pbo: 3.9 (4.7) |
| Madsbad S 2004 | lira 0.045mg QD; lira 0.225mg QD; lira 0.45mg QD; lira 0.60mg QD; lira 0.75mg QD | 12 weeks | lira 0.045mg QD: 26; 0.225mg QD: 24; 0.45mg QD: 27; 0.60mg QD: 30; 0.75mg QD: 28; pbo: 29 | lira 0.045mg QD: 53 (9.0); 0.225mg QD: 58 (7.5); 0.45mg QD: 57 (11.3); 0.60mg QD: 57 (7.7); 0.75mg QD: 58 (9.7); pbo: 57 (9.4) | lira 0.045mg QD: 84.61; 0.225mg QD: 62.5; 0.45mg QD: 66.67; 0.60mg QD: 66.67; 0.75mg QD: 57.14; pbo: 68.96 | Scandinavia, United Kingdom | not reported | lira 0.045mg QD: 4.1 (3.7); 0.225mg QD: 4.4 (4.0); 0.45mg QD: 4.5 (4.6); 0.60mg QD: 4.6 (4.6); 0.75mg QD: 6.1 (7.9); pbo: 3.4 (2.9) |
| Miyagawa J  2015 | dula 0.75 mg OW | 26 weeks | dula 0.75mg: 281; pbo: 70 | dula 0.75mg: 57.2 (9.6); pbo: 57.7 (8.3) | dula 0.75mg: 81; pbo: 79 | Japan | Japanese | dula 0.75mg: 6.8 (5.6); pbo: 6.3 (5.1) |
| Moretto TJ 2008 | exen 5μg SC BID; exen10μg SC BID | 24 weeks | exen 5μg: 77; exen 10μg: 78; pbo: 77 | exen 5μg: 54 (10); exen 10μg: 55 (10); pbo: 53 (9) | exen 5μg: 52; exen 10μg: 62; pbo 55 | USA, Puerto Rico, Romania, Russia, India | exen 5μg: White 65, Asian 29, Hispanic 6, Black 0; exen 10μg: White 72, Asian 23, Hispanic 1, Black 4; pbo: White 66, Asian 27, Hispanic 3, Black 4 | exen 5μg: 2 (3); exen 10μg: 2 (3); pbo: 1 (2) |
| Nauck MA 2016 | albi 30mg QW; albi 50mg QW | 52 weeks | albi 30mg: 101; 50mg: 99; pbo 101 | albi 30mg: 53.6 (10.9); 50mg: 52.0 (11.8); pbo: 53.1 (11.7) | albi 30mg: 57.4; 50mg: 50.5; pbo: 57.4 | USA, Mexico | albi 30mg: White 84.2, African American/African 9.9, Asian 1.0; 50mg: White 78.8, African American/African 14.1, Asian 1.0; pbo: White 78.2, African American/African 13.9, Asian 5.0. Ethnicity Hispanic/Latino: albi 30mg: 29.7; 50mg: 26.3; pbo: 28.7 | albi 30mg: 3.4 (3.7); 50mg: 4.2 (4.6); pbo 4.3 (4.0) |
| Seino Y 2008 | lira 0.1mg SC QD; lira 0.3mg SC QD; lira 0.6mg SC QD; lira 0.6mg SC QD | 14 weeks | lira 0.1mg SC QD: 45; 0.3mg SC QD: 46; 0.6mg SC QD: 45; 0.6mg SC QD: 44; pbo: 46 | lira 0.1mg SC QD: 56.5 (8.4); 0.3mg SC QD: 56.8 (8.8); 0.6mg SC QD: 60.0 (7.0); 0.6mg SC QD: 55.5 (7.6); pbo: 57.5 (8.7) | lira 0.1mg SC QD: 68.89; 0.3mg SC QD: 69.57; 0.6mg SC QD: 62.22; 0.6mg SC QD: 70.45; pbo: 63.04 | Japan | Japanese | lira 0.1mg SC QD: 7.15 (5.14); 0.3mg SC QD: 6.78 (4.69); 0.6mg SC QD: 8.87 (6.77); 0.6mg SC QD: 7.62 (4.92); pbo: 7.48 (5.65) |
| Seino Y 2014 | albi 15mg QW; albi 30mg QW; albi 30mg BIW | 16 weeks | albi 15mg QW: 52; 30mg QW: 54; 30mg BIW: 53; pbo: 53 | albi 15mg QW: 53.3 (10.3); 30mg QW: 58.0 (9.3); 30mg BIW: 59.1 (8.5); pbo: 57.5 (11.1) | albi 15mg QW: 61.5; 30mg QW: 70.4; 30mg BIW: 77.4; pbo 69.8 | Japan | Japanese | albi 15mg QW: 6.3 (4.6); 30mg QW: 7.8 (5.4); 30mg BIW: 7.2 (5.6); pbo: 6.7 (7.8) |
| Sorli C 2017 | sema 0.5mg SC QD; sema 1.0mg SC QD | 30 weeks | sema 0.5mg SC QD: 128; 1.0mg SC QD: 130; pbo: 129 | sema 0.5mg SC QD: 54.6 (11.1); 1.0mg SC QD: 52.7 (11.9); pbo: 53.7 (11.3) | sema 0.5mg SC QD: 47; 1.0mg SC QD: 62; pbo: 54 | Canada, Italy, Japan, Mexico, Russia, South Africa, UK, USA | ETHNIC Origin: sema 0.5mg SC QD: Hispanic/Latino 27; sema 1.0mg SC QD hispanic/latino: 35; pbo hispanic/latino: 28; RACE: sema 0.5mg SC QD White 65, Black or African American 9, Asian 20; sema 1.0 mg SC QD White 68, Black or African American 8, Asian 19; pbo: White 64, Black or African American 8, Asian 21 | sema 0.5mg SC QD: 4.81 (6.1); 1.0mg SC QD: 3.62 (4.88); pbo: 4.06 (5.48) |
| Terauchi Y  2014 | dula 0.25mg OW, dula 0.50mg OW, dula 0.75mg OW | 12 weeks | dula 0.25mg: 36, dula 0.50mg: 37, dula 0.75mg: 35; pbo: 37 | dula 0.25mg: 52.3 (8.8), dula 0.50mg: 52.5 (9.2), dula 0.75mg: 52.2 (7.8); pbo: 51.7 (9.7) | dula 0.25mg: 75.0, dula 0.50mg: 62.2, dula 0.75mg: 80.0; pbo: 78.4 | Japan | Japanese | dula 0.25mg: 4.3 (3.5), dula 0.50mg: 4.9 (4.0), dula 0.75mg: 4.6 (4.5); pbo: 4.7 (4.5) |
| Vilsbøll T 2007 | lira 0.65mg QD; lira 1.25mg QD; lira 1.90 mg QD | 14 weeks | lira 1.90mg: 41; 1.25mg: 42; 0.65mg: 40; pbo: 40 | lira 1.90mg: 55.4 (11.4); 1.25mg: 53.8 (10.7); 0.65mg: 56.5 (9.3); pbo: 57.7 (8.2) | lira 1.90mg: 73.17; 1.25mg: 54.76; 0.65mg: 67.50; pbo: 47.50 | Denmark, France, Netherlands, Slovakia | not reported | lira 1.90mg: 4.0 (1-29); 1.25mg: 7.0 (0-21); 0.65mg: 6.0 (1-25); pbo: 5.0 (1-23) |

**Characteristics of Included Studies – GLP1RA Monotherapy continued**

| **Study ID** | **Baseline A1C % (SD)** | **Mean Change in HbA1C % vs. baseline** | **Mean Difference in HbA1c % vs pbo** | **HYPO def** | **Ascertainment of Hypo** | **Rescue Medication** | **Excl of Pts w events? At screening or during study)** |
| --- | --- | --- | --- | --- | --- | --- | --- |
| Fonseca VA 2012 | 2-step dose increase: 7.98 (0.9); 1-step dose increase: 8.07 (0.9); pbo: 8.07 (0.9) | [LSM] 2-step dose increase: -0.77; 1-step dose increase: -0.94; pbo: -0.27 | [LSM] [no sd] 2-step dose increase: -0.54; 1-step dose increase: -0.66 | Symptomatic hypoglycemia was defined as symptoms consistent with hypoglycemia, with accompanying blood glucose <3.3 mmol/L (60 mg/dL) and/or prompt recovery with carbohydrate. Severe symptomatic hypoglycemia was defined as symptomatic hypoglycemia in which the patient required the assistance of another person and which was associated either with a plasma glucose level <36 mg/dL (2.0 mmol/L) or, if no plasma glucose measurement was available, with prompt recovery with carbohydrate. | Safety and tolerability were assessed  by physical examination, blood pressure,  heart rate, 12-lead electrocardiogram,  standard laboratory measurements, anti lixisenatide antibodies, and adverse  events reporting (including, in particular,  symptomatic and severe symptomatic  hypoglycemia, local intolerability at injection  site, allergic or allergic-like reactions,  suspected pancreatitis, and major  cardiovascular events). | Rescue (metformin) permitted. | no |
| Grunberger G 2012 | dula 0.1mg QW: 7.1 (0.6); 0.5mg: 7.2 (0.6); 1.0mg: 7.3 (0.7); 1.5mg: 7.3 (0.4); pbo: 7.4 (0.6) | [LSM] dula 0.1mg QW: -0.37 (-0.69 to -0.06); 0.5mg: -0.89 (-1.21 to -0.57); 1.0mg: -1.04 (-1.36 to -0.72); 1.5mg: -1.04 (-1.39 to -0.70); pbo: 0.01 (SE 0.13) | [LSM] manually calculated dula 0.1mg QW: -0.38; 0.5mg: -0.90; 1.0mg: -1.05; 1.5mg: -1.05 | Referencing the American Diabetes Association definition, hypoglycaemia was defined as plasma glucose ≤ 3.9 mmol (≤ 70 mg⁄dl) and ⁄ or symptoms and ⁄ or signs attributable to hypoglycaemia. Severe hypoglycaemia was defined as an episode requiring the assistance of another person to actively administer therapy | not reported | Use of additional oral antidiabetes drugs was permitted only when needed for rescue. | no |
| Madsbad S 2004 | lira 0.045mg QD: 7.4 (0.8); 0.225mg QD: 7.9 (0.8); 0.45mg QD: 7.7 (1.0); 0.60mg QD: 7.4 (1.2); 0.75mg QD: 7.4 (0.9); pbo: 7.4 (1.2) | lira 0.045mg QD: 0.25; 0.225mg QD: -0.34; 0.45mg QD: -0.30; 0.60mg QD: -0.70; 0.75mg QD: -0.75; pbo: n/a | not reported | Symptoms and Minor reported. Minor hypoglycemia defined in the study as a blood glucose < 2.8 mmol/l, thus included as severe. | Safety parameters (adverse events, hypoglycemic episodes, weight, standard hematology and biochemistry profile, vital signs, and electrocardiogram) were assessed at each visit; Each patient was seen on seven occasions: screening; baseline; after 1, 4, 8, and 12 weeks of treatment; and follow-up.  Patients were supplied with a blood glucose meter (One Touch Profile Glucometer) and instructed in its use. Fasting blood glucose was measured every morning. HbA1c,  fasting serum glucose, insulin, C-peptide, and glucagon were measured every 4 weeks. | not reported | no |
| Miyagawa J  2015 | dula 0.75mg: 8.15 (0.77); pbo: 8.20 (0.83) | dula 0.75mg: -1.43 (SE 0.05); pbo: 0.14 (0.10) | -1.57 (95% CI -1.79 to -1.35) | Hypoglycaemia was defined as a blood glucose concentration ≤3.9mmol/l. Severe hypoglycaemia was defined as an episode that required the assistance of another person to actively administer carbohydrate, glucagon or other resuscitative actions. | Rescue therapy permitted. | not reported | no |
| Moretto TJ 2008 | exen 5μg: 7.9 (1.0); exen 10μg: 7.8 (1.0); pbo: 7.8 (0.9) | [LSM] exen 5μg: -0.7 (0.1); exen 10μg: -0.9 (0.1); pbo: -0.2 (0.1) | [LSM] manually calculated exen 5μg: -0.5 exen 10μg: -0.7 | Hypoglycemia was defined as signs or symptoms associated with hypoglycemia, or an SMBG value <64 mg/dL (3.5 mmol/L), regardless of whether this concentration was  considered to be associated with signs, symptoms, or treatment. Severe hypoglycemia was defined as an episode  with signs or symptoms consistent with hypoglycemia during which the patient required the assistance of  another person and that was associated with an SMBG value <54 mg/dL (3.0 mmol/L) or prompt recovery after administration  of oral carbohydrate, glucagon injection, or IV glucose. | Patient-reported | not reported | no |
| Nauck MA 2016 | albi 30mg: 8.0 (0.8); 50mg: 8.2 (0.9); pbo 8.0 (0.9) | [LSM] not reported | [LSM] albi 30mg: -0.84 (-1.11, -0.58); 50mg: -1.04 (-1.31, -0.77) | American Diabetes Association criteria; Severe event requiring another person to administer a resuscitative action;  Documented symptomatic—plasma glucose concentration ≤3.9 mmol/l (70 mg/dl) and presence of hypoglycemic symptoms. | not reported | Rescue medication (metformin, sulfonylureas insulin and sitagliptin) permitted. HYPOGLYCEMIA COUNT IS PRE-RESCUE. | no |
| Seino Y 2008 | lira 0.1mg SC QD: 8.50 (0.84); 0.3mg SC QD: 8.24 (0.92); 0.6mg SC QD: 8.21 (0.83); 0.6mg SC QD: 8.12 (0.98); pbo: 8.43 (1.02) | not reported | lira 0.1mg SC QD: -0.79 (-1.08, -0.50); 0.3mg SC QD: -1.22 (-1.50, -0.93); 0.6mg SC QD: -1.64 (-1.93, -1.35); 0.6mg SC QD: -1.85 (-2.14, -1.56) ANOVA model with dose group and pre-treatment as fixed effects and baseline value as covariate | Minor was defined as symptomatic events confirmed by plasma glucose <3.1 mmol/L. Major not defined. | Seven-point plasma glucose profile which  was measured before and approximately 2 h after each meal, and at bedtime by self-monitoring at home using glucose  meters before start of treatment and end of  study, | not reported | no |
| Seino Y 2014 | albi 15mg QW: 8.54 (0.82); 30mg QW: 8.54 (0.86); 30mg BIW: 8.54 (0.81); pbo: 8.51 (0.73) | albi 15mg QW: -0.63 ( -0.61); 30mg QW: -1.29 (-1.27); 30mg BIW: -0.84 (-0.82); pbo: 0.27 (0.28) | albi 15mg QW: -0.89 (-1.22, -0.57); 30mg QW: -1.55 (-1.88, -1.23); 30mg BIW: -1.10 (-1.43, -0.78) [model-adjusted difference from pbo] | not reported. | not reported | not reported | no |
| Sorli C 2017 | sema 0.5mg SC QD: 8.09 (0.89); 1.0mg SC QD: 8.12 (0.81); pbo 7.95 (0.85) | sema 0.5mg SC QD: -1.45 (-1.65, -1.26); 1.0mg SC QD: -1.55 (-1.74, -1.36); pbo: -0.02 (-0.23 to 0.18) | sema 0.5mg SC QD: -1.43 (-1.71, -1.15); 1.0mg SC QD: -1.53 (-1.81, -1.25) | Severe hypoglycaemia or blood glucose-confirmed hypoglycaemia (<3·1 mmol/L | not reported | Rescue permitted. Data before initiation of any rescue medication used. | no |
| Terauchi Y  2014 | dula 0.25mg: 8.1 (0.7), dula 0.50mg: 8.0 (0.7), dula 0.75mg: 8.0 (0.6); pbo: 8.0 (0.6) | [LSM (95% CI)] dula 0.25mg: -0.90 (-1.07 to -0.73), dula 0.50mg: -1.15 (-1.32 to -0.98), dula 0.75mg: -1.35 (-1.53 to -1.18); pbo: -0.18 (-0.36 to -0.01) | [LSM (95% CI)] dula 0.25mg: -0.72 (-0.95 to -0.48), dula 0.50mg: -0.97 (-1.20 to -0.73), dula 0.75mg: -1.17 (-1.41 to -0.93) | Per the American Diabetes Association definition, hypoglycaemia was defined as PG ≤70 mg⁄dL and⁄or symptoms and⁄or signs attributable to hypoglycaemia.  Severe hypoglycaemia was defined as an episode requiring the assistance of another person to actively administer therapy. | not reported | Rescue permitted. Data before initiation of any rescue medication used. | no |
| Vilsbøll T 2007 | lira 1.90mg: 8.5 (0.9); 1.25mg: 8.3 (0.8); 0.65mg: 8.1 (0.6); pbo: 8.2 (0.7) | lira 1.90mg: -1.45; 1.25mg: -1.4; 0.65mg: -0.98; pbo: 0.29 | lira 1.90mg: -1.74 (-2.18 to -1.29); 1.25mg: -1.69 (-2.13 to -1.24); 0.65mg: -1.27 (-1.72 to -0.82) | Major or minor | not reported | not reported | no |

**Characteristics of Included Studies – SGLT2i Monotherapy**

| **Study ID** | **Dose** | **Study Duration** | **n=** | **Mean Age (SD)** | **Gender (% male)** | **Countries Studied** | **Ethnicity (%)** | **Duration of Diabetes in Years (SD)** |
| --- | --- | --- | --- | --- | --- | --- | --- | --- |
| Bailey CJ 2012 | dapa 1mg QD; dapa 2.5mg QD; dapa 5mg QD | 24 weeks | dapa 1mg QD: 72; 2.5mg QD: 74; 5mg QD: 68; pbo: 68 | dapa 1mg QD: 53.7 (9.04); 2.5mg QD: 53.5 (10.61); 5mg QD: 51.3 (11.51); pbo: 53.5 (11.08) | dapa 1mg QD: 52.8; 2.5mg QD: 45.9; 5mg QD: 47.1; pbo: 54.4 | USA, Canada, Mexico, Russia, India, South Africa, Puerto Rico | not reported | dapa 1mg QD: 1.6 (2.55); 2.5mg QD: 1.5 (2.19); 5mg QD: 1.4 (3.24); pbo: 1.1 (1.95) |
| Ferrannini E 2010 | dapa 2.5mg QD; dapa 5mg QD; dapa 10mg QD in the MORNING  (evening doses were exploratory) | 24 weeks | dapa 2.5mg QD: 65; 5mg QD: 64; 10mg QD: 70; pbo: 75 | dapa 2.5mg QD: 53.0 (11.7); 5mg QD: 52.6 (10.9); 10mg QD: 50.6 (9.97); pbo 52.7 (10.3) | dapa 2.5mg QD: 55.4; 5mg QD: 48.4; 10mg QD: 48.6; pbo: 41.3 | USA, Canada, Mexico, Russia | not reported | [MEDIAN] dapa 2.5mg QD: 0.50 (0.1 to 2.90); 5mg QD: 0.25 (0.10 to 1.40); 10mg QD: 0.45 (0.10 to 3.40); pbo: 0.50 (0.10 to 3.40) |
| Ferrannini E 2013 | empa 5mg QD; empa 10mg QD; empa 25mg QD | 12 weeks | empa 5mg QD: 81; 10mg QD: 81; 25mg QD: 82; pbo: 82 | empa 5mg QD: 59.0 (37-78); 10mg QD: 58.0 (30-76); 25mg QD: 57.0 (30-79); pbo: 58.0 (28-80) (median range) | empa 5mg QD: 56.8; 10mg QD: 49.4; 25mg QD: 50.0; pbo: 54.9 | Argentina, Croatia, Estonia, Germany, Italy, korea, Lithuania, Romania, Russia, Sweden, Slovakia, Taiwan, Ukraine | empa 5mg QD: White 63.0, Asian 35.8, Other 1.2; 10mg QD: White 64.2, Asian 34.6, Other 1.2; 25mg QD: White 65.9, Asian 32.9, Other 1.2; pbo: White 65.9, Asian 34.1, Other 0 | not reported |
| Fonseca VA 2013 | ipra 12.5mg QD; ipra 50mg QD; ipra 150mg QD; ipra 300mg QD | 12 weeks | ipra 12.5mg QD: 70; 50mg QD: 67; 150mg QD: 68; 300mg QD: 68; pbo: 69 | ipra 12.5mg QD: 53.9 (9.6); 50mg QD: 52.6 (10.7); 150mg QD: 54.2 (10.3); 300mg QD: 54.2 (10.7); pbo: 53.4 (9.7) | ipra 12.5mg QD: 55.7; 50mg QD: 50.7; 150mg QD: 42.6; 300mg QD: 54.4; pbo: 46.4 | not reported | ipra 12.5mg QD: White 58.6, Non-white 41.4; 50mg QD: White 52.2, Non-white 47.8; 150mg QD: White 54.4, Non-white 45.6; 300mg QD: White 52.9, Non-white 47.1; pbo: White 47.8, Non-white 52.2 | ipra 12.5mg QD: 4.08 (3.24); 50mg QD: 4.61 (4.65); 150mg QD: 5.11 (6.46); 300mg QD: 4.48 (4.91); pbo: 4.64 (5.93) |
| Inagaki N 2013 | cana 50mg QD; cana 100mg QD; cana 200mg QD; cana 300mg QD | 12 weeks | cana 50mg QD: 82; 100mg QD: 74; 200mg QD: 76; 300mg QD: 75; pbo: 75 | cana 50mg QD: 57.4 (10.8); 100mg QD: 57.7 (10.5); 200mg QD: 57.0 (1.07); 300mg QD: 57.1 (10.1); pbo: 57.7 (11.0) | cana 50mg QD: 61; 100mg QD: 70.3; 200mg QD: 64.5; 300mg QD: 73.3; pbo 72.0 | Japan | Japanese | not reported |
| Inagaki N 2014 | cana 100mg QD; cana 200mg QD | 24 weeks | cana 100mg QD: 90; 200mg QD: 88; pbo: 93 | cana 100mg QD: 58.4 (10.4); 200mg QD: 57.4 (11.1); pbo: 58.2 (11.0) | cana 100mg QD: 65.6; 200mg QD: 81.8; pbo: 64.5 | Japan | Japanese | cana 100mg QD: 4.72 (4.59); 200mg QD: 5.88 (5.93); pbo: 5.63 (5.76) |
| Ji L 2014 | dapa 5mg QD; dapa 10mg QD | 24 weeks | dapa 5mg: 128; 10mg: 133; pbo: 132 | dapa 5mg: 53.0 (11.07); 10mg: 51.2 (9.89); pbo: 49.9 (10.87) | dapa 5mg: 65.6; 10mg: 64.7; pbo: 65.9 | China, South Korea, Taiwan, India | dapa 5mg: Chinese 89.1, Asian Indian 6.3, Korean 4.7, Japanese 0, Other Asian 0; 10mg: Chinese 88.0, Asian Indian 6.8, Korean 3.8, Japanese 0.8, Other Asian 0.8; pbo: Chinese 88.6, Asian Indian 6.1, Korean 3.8, Japanese 0.8, Other Asian 0.8 | dapa 5mg: 1.15 (2.0); 10mg: 1.67 (2.8); pbo: 1.30 (2.0) |
| Kadowaki T 2014 | empa 5mg QD; empa 10mg QD; empa 25mg QD; empa 50mg QD | 12 weeks | empa 5mg QD: 110; 10mg QD: 109; 25mg QD: 109; 50mg QD: 110; pbo: 109 | empa 5mg QD: 57.3 (11.2); 10mg QD: 57.9 (9.4); 25mg QD: 57.2 (9.7); 50mg QD: 56.6 (10.3); pbo: 58.7 (8.7) | empa 5mg QD: 76.4; 10mg QD: 70.6; 25mg QD: 77.1; 50mg QD: 77.3; pbo: 73.4 | Japan | Japanese (438) | not reported |
| Kaku K 2013 | dapa 1mg QD; dapa 2.5mg QD; dapa 5mg QD; dapa 10mg QD | 12 weeks | dapa 1mg QD: 59; 2.5mg QD: 56; 5mg QD: 58; 10mg QD: 52; pbo: 54 | dapa 1mg QD: 55.9 (9.7); 2.5mg QD: 57.7 (9.3); 5mg QD: 58.0 (9.5); 10mg QD: 56.5 (11.5); pbo: 58.4 (10.0) | dapa 1mg QD: 79.7; 2.5mg QD: 69.6; 5mg QD: 81.0; 10mg QD: 75.0; pbo: 79.6 | Japan | Japanese | dapa 1mg QD: 4.89 (4.37); 2.5mg QD: 4.41 (3.97); 5mg QD: 5.34 (4.51); 10mg QD: 4.73 (4.73); pbo: 4.74 (3.82) |
| Kaku K 2014 A | dapa 5mg QD; dapa 10mg QD | 24 weeks | dapa 5mg: 86; 10mg: 88; pbo: 87 | dapa 5mg: 58.6 (10.4); 10mg: 57.5 (9.3); pbo: 60.4 (9.7) | dapa 5mg: 58.1; 10mg: 60.2; pbo: 59.8 | Japan | Japanese | dapa 5mg: 4.59 (5.56); 10mg: 4.93 (4.52); pbo 5.29 (6.17) |
| Kaku K 2014 B | tofo 10mg QD; tofo 20mg QD; tofo 40mg QD | 24 weeks | tofo 10mg QD: 57; 20mg QD: 58; 40mg QD: 58; pbo: 56 | tofo 10mg QD: 58.6 (9.8); 20mg QD: 56.6 (10.2); 40mg QD: 57.0 (9.1); pbo: 56.8 (9.9) | tofo 10mg QD: 66.7; 20mg QD: 67.2; 40mg QD: 67.2; pbo: 66.1 | Japan | Japanese | tofo 10mg QD: 6.3(7.1); 20mg QD: 6.4 (5.1); 40mg QD: 6.7 (5.5) |
| Kashiwagi A 2014 | ipra 12.5mg QD; ipra 25mg QD; ipra 50mg QD; ipra 100mg QD | 12 weeks | ipra 12.5mg QD: 73; 25mg QD: 74; 50mg QD: 72; 100mg QD: 72; pbo: 69 | ipra 12.5mg QD: 55.3 (10.2); 25mg QD: 57.0 (10.4); 50mg QD: 55.9 (11.4); 100mg QD: 56.0 (10.4); pbo: 55.2 (9.7) | ipra 12.5mg QD: 58.90; 25mg QD: 66.22; 50mg QD: 59.72; 100mg QD: 68.06; pbo: 71.01 | Japan | not reported | manually calculated: ipra 12.5mg QD: 6.31; 25mg QD: 6.40; 50mg QD: 6.64; 100mg QD: 7.8; pbo: 6.31 |
| Kashiwagi  A 2015 | ipra 50mg  QD | 16 weeks | ipra 50mg QD: 62; pbo: 67 | ipra 50mg: 60.6 (9.4); pbo: 58.3 (10.5) | ipra 50mg: 67.7; pbo: 71.6 | Japan | Japanese | ipra 50mg: 7.53 (6.88); pbo: 5.9 (5.09) |
| List JF  2009 | dapa 2.5mg QD; dapa 5mg QD; dapa 10mg QD; dapa 20mg QD; dapa 50mg QD | 12 weeks | dapa 2.5mg: 59; dapa 5mg: 58, dapa 10mg: 47; dapa 20mg: 59; dapa 50mg: 56; pbo: 54 | dapa 2.5mg: 55 (11); dapa 5mg: 55 (12), dapa 10mg: 54 (9); dapa 20mg: 55 (10); dapa 50mg: 53 (10); pbo: 53 (11) | dapa 2.5mg: 49; dapa 5mg: 48, dapa 10mg: 53; dapa 20mg: 54; dapa 50mg: 45; pbo: 56 | Canada, Mexico, Puerto Rico, US | not reported | not reported |
| Roden 2013 | empa 10mg QD; empa 25mg QD | 12 weeks | empa 10mg: 224; 25mg: 224; pbo: 228 | empa 10mg: 56.2 (11.6); 25mg: 53.8 (11.6); pbo: 54.9 (10.9) | empa 10mg: 63; 25mg: 65; pbo: 54 | Belgium, Canada, China, Germany, India, Ireland, Japan, Switzerland and USA | empa 10mg: Asian 64, White 34, Black/African-American 1, American-Indian/Alaska Native 0, Hawaiian/Pacific Islander 1, 25mg: Asian 64, White 33, Black/African-American 3, American-Indian/Alaska Native 0, Hawaiian/Pacific Islander 0; pbo: Asian 64, White 33, Black/African-American 3, American-Indian/Alaska Native 0, Hawaiian/Pacific Islander 0 | empa 10mg: ≤1 year: 39, >1-5 years: 41, >5-10 years: 13, >10 years: 7; 25mg: ≤1 year: 41, >1-5 years: 37, >5-10 years: 17, >10 years 6; pbo: ≤1 year: 32, >1-5 years: 46, >5-10 years: 15, >10 years: 8 |
| Seino Y 2014 A | luseo 1mg QD; luseo 2.5mg QD; luseo 5mg QD; luseo 10mg QD | 12 weeks | luseo 1mg QD: 55; 2.5mg QD: 56; 5mg QD: 54; 10mg QD: 58; pbo: 57 | luseo 1mg QD: 58.5 (9.1); 2.5mg QD: 57.4 (9.3); 5mg QD: 57.3 (11.4); 10mg QD: 59.6 (7.8); pbo: 57.1 (10.0) | luseo 1mg QD: 72.7; 2.5mg QD: 67.9; 5mg QD: 75.9; 10mg QD: 63.8; pbo: 71.9 | Japan | not reported | luseo 1mg QD: 4.7 (4.1); 2.5mg QD: 4.6 (4.4); 5mg QD: 4.5 (4.2); 10mg QD: 6.2 (5.4); pbo 5.1 (4.6) |
| Seino Y 2014 B | luseo 0.5mg QD; luseo 2.5mg QD; luseo 5mg QD | 12 weeks | luseo 0.5mg QD: 60; 2.5mg QD: 61; 5mg QD: 61; pbo: 54 | luseo 0.5mg QD: 55.2 (10.1); 2.5mg QD: 58.3 (9.4); 5mg QD: 56.8 (9.3); pbo: 57.6 (11.0) | luseo 0.5mg QD: 68.3; 2.5mg QD: 57.4; 5mg QD: 72.1; pbo: 74.1 | Japan | not reported | luseo 0.5mg QD: 4.90 (4.49); 2.5mg QD: 6.15 (6.50); 5mg QD: 5.77 (5.55); pbo: 7.30 (6.43) |
| Stenlöf K 2013 | cana 100mg QD; cana 300mg QD | 26 weeks | cana 100mg QD: 195; 300mg QD: 197; pbo: 192 | cana 100mg QD: 55.1 (10.8); 300mg QD: 55.3 (10.2); pbo: 55.7 (10.9) | cana 100mg QD: 41.5; 300mg QD: 45.2; pbo: 45.8 | 17 countries; not specified | cana 100mg QD: White 63.6, Black or African American 9.2, Asian 13.8, Other 13.3; 300mg QD: White 69.5, Black or African American 7.1, Asian 14.7, Other 8.6; pbo: White 69.8, Black or African American 4.7, Asian 15.1, Other 10.4 | cana 100mg QD: 4.5 (4.4); 300mg QD: 4.3 (4.7); pbo: 4.2 (4.1) |
| Terra SG 2017 | ertu 5mg QD; ertu 15mg QD | 52 weeks | ertu 5mg QD: 156; 15mg QD: 152; pbo: 153 | ertu 5mg QD: 56.8 (11.4); 15mg QD: 56.2 (10.8); pbo: 56.1 (10.9) | ertu 5mg QD: 57.1; 15mg QD: 59.2; pbo: 53.6 | USA, Canada, Israel, Italy, Mexico, South Africa, UK | ertu 5mg QD: American Indian or Alaska Native 0, Asian 6.4, Black or African-American 6.4, Multiple 1.3, White 85.9; 15mg QD: American Indian or Alaska Native 0, Asian 9.2, Black or African-American 6.6, Multiple 1.3, White 82.9; pbo: American Indian or Alaska Native 0.7, Asian 9.8, Black or African-American 5.9, Multiple 1.3, White 82.4 | ertu 5mg QD: 5.11 (5.09); 15mg QD: 5.22 (5.55); pbo: 4.63 (4.52) |

**Characteristics of Included Studies – SGLT2i Monotherapy continued**

| **Study ID** | **Baseline A1C % (SD)** | **Mean Change in HbA1C % vs. baseline** | **Mean Difference in HbA1c % vs pbo** | **HYPO def** | **Ascertainment of Hypo** | **Rescue Medication** | **Excl of Pts w events? At screening or during study)** |
| --- | --- | --- | --- | --- | --- | --- | --- |
| Bailey CJ 2012 | dapa 1mg QD: 7.8 (0.98); 2.5mg QD: 8.1 (1.07); 5mg QD: 7.9 (1.03); pbo: 7.8 (1.12) | dapa 1mg QD: -0.68 (-0.91 to -0.45); 2.5mg QD: -0.72 (-0.95 to -0.49); 5mg QD: -0.82 (-1.06 to -0.58); pbo: 0.02 (-0.22 to 0.25) | dapa 1mg QD: -0.69 (-1.02, -0.37); 2.5mg QD: -0.74 (-1.07, -0.41); 5mg QD: -0.84 (-1.17, -0.50) | Major hypoglycaemia was defined as a symptomatic episode that required third-party assistance and was associated with plasma glucose <3.00 mmol/l (<54 mg/dl) and prompt recovery after glucose or glucagon administration. Minor hypoglycaemia was either a symptomatic or asymptomatic episode associated with plasma glucose <3.50 mmol/l (<63 mg/dl), that does not qualify as a major episode. Additional accounts of hypoglycemic symptoms that did not meet these criteria were categorized as ‘other’. | not reported | Rescue confounding. Measurements obtained after rescue were not included in the efficacy analyses but were included in the safety analysis. | no |
| Ferrannini E 2010 | dapa 2.5mg QD: 7.92 (0.90); 5mg QD: 7.86 (0.94); 10mg QD: 8.01 (0.96); pbo: 7.84 (0.87) | dapa 2.5mg QD: -0.58 (0.11); 5mg QD: -0.77 (0.11) (p=0.0005); 10mg QD: -0.89 (0.11) (p<0.0001); pbo: -0.23 (0.10) | manually calculated dapa 2.5mg QD: -0.35; 5mg QD: -0.54; 10mg QD: -0.66  Assessed in patients without missing baseline and week 24 values with last observation carried forward; Mean value after adjustment for baseline  value. | Severe: major episode, defined as a symptomatic episode requiring third-party assistance due to severe impairment in consciousness or behavior, with a capillary or plasma glucose value 54 mg/dl (3.0 mmol/L), and prompt recovery after glucose or glucagon administration. | Patient-reported. Patients were instructed to self-monitor their blood glucose daily and to report any unusually high or low blood glucose event or any symptoms suggestive of hypoglycemia. | Rescue confounding. Rescue (metformin) permitted. Data obtained after rescue were excluded from efficacy analyses but not safety analysis. | no |
| Ferrannini E 2013 | empa 5mg QD: 7.9 (0.8); 10mg QD: 8.0 (0.8); 25mg QD: 7.8 (0.8); pbo: 7.8 (0.8) | empa 5mg QD: -0.4 (-0.61 to -0.25); 10mg QD: -0.5 (-0.66 to -0.30); 25mg QD: -0.6 (-0.81 to -0.45); pbo: 0.1 (-0.09 to 0.27) | manually calculated empa 5mg QD: -0.5; 10mg QD: -0.6; 25mg QD: -0.7 | Symptomatic [not specified] or laboratory-documented [values not given] | not reported | not reported | no |
| Fonseca VA 2013 | ipra 12.5mg QD: 7.95 (0.78); 50mg QD: 8.05 (0.81); 150mg QD: 7.83 (0.65); 300mg QD: 7.90 (0.67); pbo: 7.84 (0.78) | [LSM] ipra 12.5mg QD: -0.22; 50mg QD: -0.39; 150mg QD: -0.47; 300mg QD: -0.55; pbo: 0.26 (LOCF) | [LSM] ipra 12.5mg QD: -0.49 (-0.73 to -0.24); 50mg QD: -0.65 (-0.90 to -0.40); 150mg QD: -0.73 (-0.98 to -0.48); 300mg QD: -0.81 (-1.06 to -0.56) | Serious, as defined by development of hypoglycemic coma requiring  hospitalization, or requiring discontinuation of study drug. | Patient-reported (blood glucose unconfirmed). Patients were provided with a glucometer (Roche Accu-Chek®;  Hoffmann-La Roche Ltd, Basel, Switzerland) and asked to monitor  capillary blood glucose twice daily (fasted and 2 h after a meal) and  also, if symptoms of hypoglycemia occurred. | not reported | no |
| Inagaki N 2013 | cana 50mg QD: 8.13 (0.78); 100mg QD: 8.05 (0.86); 200mg QD: 8.11 (0.88); 300mg QD: 8.17 (0.81); pbo: 7.99 (0.77) | [LSM] cana 50mg QD: -0.61 (p<0.01); 100mg QD: -0.80 (p<0.01); 200mg QD: -0.79 (p<0.01); 300mg QD: -0.88 (p<0.01); pbo: 0.11 | [LSM] manually calculated cana 50mg QD: -0.72; 100mg QD: -0.91; 200mg QD: -0.90; 300mg QD: -0.99  data represents last observation carried forward | not reported | Hypoglycemic symptoms, were recorded throughout the study. | not reported | no |
| Inagaki N 2014 | cana 100mg QD: 7.98 (0.73); 200mg QD: 8.04 (0.77); pbo: 8.04 (0.70) | [LSM] cana 100mg QD: -0.74 (0.07); 200mg QD: -0.76 (0.07); pbo: 0.29 (0.07) | [LSM] cana 100mg QD: -1.03 (0.10); 200mg QD: -1.05 (0.10) | Asymptomatic hypoglycemia -typical hypoglycemic symptoms were absent but the blood glucose level was low (≤ 70 mg/dl or 3.89 mmol/L).  Symptomatic hypoglycemia -typical hypoglycemic symptoms were present irrespective of the blood glucose level. (Severe not defined) | Patients were instructed to measure blood glucose levels  on ‡ 3 days/week using a glucose meter as early in the morning  as possible in the fasting state throughout the study. Data  were to be recorded in a patient diary. The patients were also  instructed to measure glucose levels, if possible, in the event of  symptoms suggestive of hypoglycemia. The investigator  classified hypoglycemia as either asymptomatic hypoglycemia  (typical hypoglycemic symptoms were absent but the blood  glucose level was £ 70 mg/dl) or symptomatic hypoglycemia  (typical hypoglycemic symptoms were present irrespective of  the blood glucose level). | not reported | no |
| Ji L 2014 | dapa 5mg: 8.14 (0.74); 10mg: 8.28 (0.95); pbo: 8.35 (0.95) | dapa 5mg: -1.04 (-1.18 to -0.90); 10mg: -1.11 (-1.24 to -0.98); pbo: -0.29 (-0.43 to -0.16) | dapa 5mg: -0.75; 10mg: -0.82 (SE or p value (n/a)) | Major hypoglycemia was defined as symptomatic episodes requiring external assistance due to severely impaired consciousness or behavior, with capillary or plasma glucose values <54 mg/dL (<3.0mmol/L) and prompt recovery after glucose or glucagon administration. Minor hypoglycemia was defined as any  episode (symptomatic or asymptomatic) with a capillary or plasma glucose measure <63 mg/dL (<3.5mmol/L) that did not qualify as a major episode. Other episodes of hypoglycemia were defined as episodes reported by the investigator that were suggestive of hypoglycemia but did not meet the aforementioned criteria. | not reported | Rescue confounding. Rescue (metformin) permitted. For the efficacy analysis, observations after the  initiation of rescue therapy was excluded. Safety data after rescue therapy included. | no |
| Kadowaki T 2014 | empa 5mg QD: 7.92 (0.70); 10mg QD: 7.93 (0.71); 25mg QD: 7.93 (0.78); 50mg QD: 8.02 (0.65); pbo: 7.94 (0.74) | empa 5mg QD: -0.42 (SE ± 0.089); 10mg QD: -0.40 (SE ± 0.09); 25mg QD: -0.65 (SE ± 0.09); 50mg QD: -0.61 (SE ± 0.09); pbo: 0.30 (SE ± 0.09) | empa 5mg QD: -0.72 (SE ± 0.08) (-0.87 to -0.57); 10mg QD: -0.70 (SE ± 0.08) (-0.85 to -0.55); 25mg QD: -0.95 (SE ± 0.08) (-1.10 to -0.80); 50mg QD: -0.91 (SE ± 0.08) (-1.06 to -0.76) | Confirmed events, plasma glucose <70 mg/dL (<3.9 mmol/L) and/or requiring assistance. Severe not explicitly defined but results suggest no assistance required. | patient-reported | Rescue therapy permitted and data obtained after treated as missing. | no |
| Kaku K 2013 | dapa 1mg QD: 8.10 (0.79); 2.5mg QD: 7.92 (0.74); 5mg QD: 8.05 (0.66); 10mg QD: 8.18 (0.69); pbo: 8.12 (0.71) | dapa 1mg QD: -0.12 (0.07); 2.5mg QD: -0.11 (0.07); 5mg QD: -0.37 (0.07); 10mg QD: -0.44 (0.07); pbo: 0.37 (0.07) | dapa 1mg QD: -0.49 (0.10) (-0.68 to -0.29); 2.5mg QD: -0.48 (0.10) (-0.67 to -0.28); 5mg QD: -0.74 (0.10) (-0.93 to -0.54); 10mg QD: -0.80 (0.10) (-1.00 to -0.61) | not reported | not reported | not reported | no |
| Kaku K 2014 A | dapa 5mg: 7.50 (0.72); 10mg: 7.46 (0.61); pbo: 7.50 (0.63) | [LSM] dapa 5mg: -0.41 (-0.53 to -0.29); 10mg: -0.45 (-0.57 to -0.33); pbo: -0.06 (-0.18 to 0.06) | [LSM] dapa 5mg: -0.35 (-0.52 to -0.18); 10mg: -0.39 (-0.56 to -0.23) | not reported | not reported | Rescue permitted. Efficacy data after rescue not included but did include body weight. | no |
| Kaku K 2014 B | tofo 10mg QD: 8.45 (0.75); 20mg QD: 8.34 (0.81); 40mg QD: 8.37 (0.77); pbo: 8.41 (0.78) | [LSM] tofo 10mg QD: -0.797 (-0.960 to -0.634); 20mg QD: -1.017 (-1.178 to -0.856); 40mg QD: -0.870 (-1.031 to -0.709) | [LSM] tofo 10mg QD: -0.769; 20mg QD: -0.990; 40mg QD: -0.842 (CI n/a) | Hypoglycemia was defined as either (1) signs/symptoms consistent with  hypoglycemia that resolved after ingesting food or administration of glucagon or glucose, or (2) blood glucose ≤ 50 mg/dL (2.78 mmol/L) with or without signs/symptoms of hypoglycemia. (Severe or major not defined, however events that occurred were described as mild or moderate). | Self-monitored blood glucose | not reported | Yes. severe or frequent  episodes of hypoglycemia within 4 weeks before the  screening for provisional registration. Patients with a history of infection or  hypoglycemia was excluded because of the difficulty in  evaluating the safety of tofogliflozin. |
| Kashiwagi A 2014 | ipra 12.5mg QD: 8.39 (0.90); 25mg QD: 8.32 (0.83); 50mg QD: 8.33 (0.80); 100mg QD: 8.25 (0.76); pbo: 8.36 (0.79) | ipra 12.5mg QD: -0.11 (0.09); 25mg QD: -0.47 (0.09); 50mg QD: -0.79 (0.09); 100mg QD: -0.81(0.09); pbo: 0.50 (0.09) | ipra 12.5mg QD: -0.61 (-0.85 to -0.36); 25mg QD: -0.97 (-1.21 to -0.72); 50mg QD: -1.29 (-1.54 to -1.04); 100mg QD: -1.31 (-1.55 to -1.06) ipra adjusted mean, difference vs placebo (95% CI) | not reported | not reported | not reported | no |
| Kashiwagi  A 2015 | ipra 50mg: 8.40 (0.86); pbo: 8.25 (0.68) | ipra 50mg: -0.76 (0.70); pbo: 0.54 (1.00) | -1.24 (SE 0.15) (95% CI -1.54 to -0.94) pbo-adjusted change | not reported | not reported | not reported | no |
| List JF  2009 | dapa 2.5mg: 7.6 (0.7); dapa 5mg: 8.0 (0.9); dapa 10mg: 8.0 (0.8); dapa 20mg: 7.7 (0.9); dapa 50mg: 7.8 (1.0); pbo: 7.9 (0.9) | dapa 2.5mg: -0.71 (0.09); dapa 5mg: -0.72 (0.09); dapa 10mg: -0.85 (0.11); dapa 20mg: -0.55 (0.09); dapa 50mg: -0.90 (0.10); pbo: -0.18 (0.10) | dapa 2.5mg: -0.53; dapa 5mg: -0.54; dapa 10mg: -0.67; dapa 20mg: -0.37; dapa 50mg: -0.72  manually calculated | Hypoglycemia was defined as events with a fingerstick glucose ≤2.8 mmol/L. | BG confirmation | not reported | no |
| Roden 2013 | empa 10mg: 7.87 (0.88); 25mg: 7.86 (0.85); pbo: 7.91 (0.78) | empa 10mg: -0.66 (-0.76 to -0.56); 25mg: -0.78 (-0.88 to -0.67); pbo: 0.08 (-0.03 to 0.18) | empa 10mg: -0.74 (-0.88 to -0.59); 25mg: -0.85 (-0.99 to -0.71) | Confirmed hypoglycemic adverse events (hypoglycemic episodes reported as adverse events when plasma glucose was <3·9 mmol/L, required assistance, or both). | Confirmed hypoglycemia either plasma glucose  <3·9 mmol/L or requiring assistance, or both. | Rescue medication permitted. | no |
| Seino Y 2014 A | luseo 1mg QD: 7.77 (0.79); 2.5mg QD: 8.05 (0.75); 5mg QD: 7.86 (0.69); 10mg QD: 7.95 (0.67); pbo: 7.92 (0.84) | [LSM] luseo 1mg QD: -0.29 (-0.41 to -0.17); 2.5mg QD: -0.39 (-0.51 to -0.27); 5mg QD: -0.46 (-0.58 to -0.34); 10mg QD: -0.43 (-0.54 to -0.31); pbo: 0.22 (0.10 to 0.34) | [LSM] luseo 1mg QD: -0.51 (-0.68 to -0.34); 2.5mg QD: -0.61 (-0.78 to -0.44); 5mg QD: -0.68 (-0.85 to -0.51); 10mg QD: -0.64 (-0.81 to -0.48) luseo difference vs. placebo; not adjusted | not reported | not reported | not reported | no |
| Seino Y 2014 B | luseo 0.5mg QD: 8.16 (0.93); 2.5mg QD: 8.07 (0.90); 5mg QD: 8.16 (0.96); pbo: 7.88 (0.72) | [LSM] luseo 0.5mg QD: -0.36 (-0.5 to -0.2); 2.5mg QD: -0.62 (-0.8, -0.5); 5mg QD: -0.75 (-0.9, -0.6); pbo: 0.06 (-0.1 to 0.2) | [LSM] luseo 0.5mg QD: -0.42 (-0.6 to -0.2); 2.5mg QD: -0.68 (-0.9 to -0.5); 5mg QD: -0.82 (-1.0 to -0.6) | not reported | patient-reported | not reported | no |
| Stenlöf K 2013 | cana 100mg QD: 8.1 (1.0); 300mg QD: 8.0 (1.0); pbo: 8.0 (1.0) | [LSM] cana 100mg QD: -0.77; 300mg QD: -1.03; pbo: 0.14 | [LSM] cana 100mg QD: -0.91 (-1.1 to -0.7); 300mg QD: -1.16 (-1.3 to -1.0) | Documented hypoglycaemia episodes included biochemically confirmed episodes (concurrent fingerstick or plasma glucose ≤3.9 mmol/l, irrespective of symptoms) and severe hypoglycaemia  episodes (i.e., requiring the assistance of another individual or resulting in seizure or loss of consciousness). | patient-documented fingerstick glucose test | Rescue therapy with metformin permitted.  Efficacy data post rescue not included. | no |
| Terra SG 2017 | ertu 5mg QD: 8.16 (0.88); 15mg QD: 8.35 (1.12); pbo: 8.11 (0.92) | [LSM] ertu 5mg QD: -0.79 (-0.95 to -0.63); 15mg QD: -0.96 (-1.12 to -0.80) | ertu 5mg QD: -0.99 (-1.22 to -0.76); 15mg QD: -1.16 (-1.39 to -0.93) | The endpoint of symptomatic hypoglycaemia consisted of episodes with clinical symptoms of hypoglycaemia reported by the investigator (i.e., biochemical documentation [FPG or finger-stick glucose] was not required]. Documented hypoglycaemia, defined as episodes with a glucose level ≤3.9 mmol/L, with or without symptoms, was also analyzed. Severe hypoglycaemia was defined as an episode that required assistance, either medical or non-medical. | investigator-confirmed | Rescue confounding only if severe. Safety analyses included all randomized participants who received at least one dose of study medication and excluded data after initiation of rescue medication with the exception of summaries of serious AEs, deaths, and discontinuations because of AEs, postural blood pressure and pulse rate which included data after rescue. | no |

**Characteristics of Included Studies – DPP4i Added to Metformin Background**

| **Study ID** | **Dose** | **Study Duration** | **n=** | **Mean Age (SD)** | **Gender (% male)** | **Countries Studied** | **Ethnicity (%)** | **Duration of Diabetes in Years (SD)** |
| --- | --- | --- | --- | --- | --- | --- | --- | --- |
| Ahrén B  2004 | vilda  50mg  QD | 12 weeks | vilda 50mg: 56; pbo: 51 | vilda 50mg: 57.9  pbo: 55.7 | vilda 50mg: 69.6  pbo: 66.7 | not reported | not reported | vilda 50mg: 5.6 (4.2); pbo: 5.5 (3.7) |
| Amin NB 2015 | sita 100mg QD | 12 weeks | sita 100mg: 55; pbo: 54 | sita 100mg: 53.3 (10.7); pbo 54 (8.1) | sita 100mg: 72.7; pbo: 55.6 | Canada, India, South Korea, Mexico, USA | not reported | sita 100mg: 6.3 (0.3 to 20.0); pbo: 6.4 (0.3 to 20.5) |
| Bosi E 2007 | vilda 50mg QD; vilda 100mg QD | 24 weeks | vilda 50mg QD: 177; 100mg QD: 185; pbo 182 | vilda 50mg QD: 54.3 (9.7); 100mg QD: 53.9 (9.5); pbo 54.5 (10.3) | vilda 50mg QD: 57.3; 100mg QD: 61.5; pbo: 53.1 | USA, France, Italy, Sweden | vilda 50mg QD: Caucasian 74.1, Hispanic or Latino 16.8, Black 6.3, All other 2.8; 100mg QD: Caucasian 74.1, Hispanic or Latino 13.3, Black 9.1, All other 3.5; pbo: Caucasian 73.1, Hispanic or Latino 18.5, Black 6.9, All other 1.5 | vilda 50mg QD: 6.8 (5.5); 100mg QD: 5.8 (4.7); pbo: 6.2 (5.3) |
| Bryson A  2016 | teneli  5mg QD; teneli 10mg QD; teneli 20mg QD; teneli 40mg QD | 24 weeks | teneli  5mg: 87; 10mg: 93; 20mg: 91; 40mg: 88; pbo: 88 | teneli  5mg: 58.8 (Range 45-70);10mg: 58.5 (38-73); 20mg: 58.3 (28-78); 40mg: 58.2 (35-78); pbo: 58.9 (39-76) | teneli  5mg: 52.9; 10mg: 54.8; 20mg: 61.5; 40mg: 59.1; pbo: 53.4 | 55 European centers | teneli 5mg: Caucasian 100;10mg: Caucasian 100; 20mg: Caucasian 97.8; 40mg: Caucasian 98.9; pbo: Caucasian 100 | teneli  5mg: 4.75 ;10mg: 4.81; 20mg: 5.30; 40mg: 5.15; pbo: 4.84 |
| Charbonnel B 2006 | sita 100mg QD | 24 weeks | sita 100mg: 464; pbo: 237 | sita 100mg: 54.4 (10.4); pbo: 54.7 (9.7) | sita 100mg: 55.8; pbo: 59.5 | not reported | sita 100mg: Asian 10.6, Black 6.7, Hispanic 15.5, White 63.1, Other 4.1; pbo: Asian 11.0, Black 5.9, Hispanic 11.8, White 67.1, Other 4.2 | sita 100mg: 6.0 (5.0); pbo: 6.6 (5.5) |
| DeFronzo RA 2009 | saxa 2.5mg QD; saxa 5mg QD; saxa 10mg QD | 24 weeks | saxa 2.5mg QD: 192; 5mg QD: 191; 10mg QD: 181; pbo: 179 | saxa 2.5mg QD: 54.7 (10.1); 5mg QD: 54.7 (9.6); 10mg QD: 54.2 (10.1); pbo: 54.8 (10.2) | saxa 2.5mg QD: 43.2; 5mg QD: 53.9; 10mg QD: 52.5; pbo: 53.6 | not reported | saxa 2.5mg QD: Caucasian 79.7, African American 4.2, Asian 4.2, Other 12.0; 5mg QD: Caucasian 83.2, African American 5.8, Asian 1.6, Other 9.4; 10mg QD: Caucasian 79.6, African American 7.7, Asian 2.8, Other 9.9; pbo: Caucasian 83.8, African American 3.9, Asian 2.2, Other 10.1 | saxa 2.5mg QD: 6.7 (5.6); 5mg QD: 6.4 (4.7); 10mg QD: 6.3 (4.4); pbo: 6.7 (5.6) |
| Derosa G  2012 | sita 100mg QD | 52 weeks | sita 100mg: 91; pbo: 87 | sita 100mg: 55.9 (8.8); pbo: 54.8 (7.9) | sita 100mg: 42; pbo: 44 | Italy | not reported | sita 100mg: 0.48; pbo: 0.45 |
| Derosa G  2012 | vilda 50mg BID | 52 weeks | vilda 50mg: 84; pbo: 83 | vilda 50mg: 54.2 (8.3); pbo: 52.4 (7.1) | vilda 50mg: 42; pbo: 43 | Italy | not reported | vilda 50mg: 0.51; pbo: 0.53 |
| Forst T 2010 | lina 1mg QD; lina 5mg QD; lina 10mg QD | 12 weeks | lina 1mg QD: 65; 5mg QD: 66; 10mg QD: 66; pbo: 71 | lina 1mg QD: 59.2 (8.4); 5mg QD: 59.6 (9.8);10mg QD: 61.8 (8.8); pbo: 60.1 (8.1) | lina 1mg QD: 55.4; 5mg QD: 56.1; 10mg QD: 53.0; pbo: 62.0 | UK, Germany, France, Slovakia, Ukraine and Sweden | lina 1mg QD: White 98, Black 0, Asian 2; 5mg QD: White 100, Black 0, Asian 0; 10mg QD: White 98, Black 2, Asian 0; White 98, Black 1, Asian 1 (manually calculated to add to 100) | lina 1mg QD: 6.9 (5.9); 5mg QD: 7.3 (7.5); 10mg QD: 8.2 (6.8); pbo: 6.2 (5.1) |
| Goodman M 2009 | vilda 100mg QD AM; 100mg QD PM | 24 weeks | vilda 100mg QD AM: 125; 100mg QD PM: 123; pbo: 122 | vilda 100mg QD AM: 54.7 (10.3); 100mg QD PM: 55.2 (11.4); pbo: 54.5 (9.7) | vilda 100mg QD AM: 52.8; 100mg QD PM: 52.8; pbo: 52.8 | USA, Europe (not a country) | vilda 100mg QD AM: Caucasian 63.2, Black 8.8, Asian 0.8, Hispanic or Latino 24.8, Pacific Islander 0.8, Other 1.6; 100mg QD PM: Caucasian 68.3, Black 7.3, Asian 1.6, Hispanic or Latino 22.8, Pacific Islander 0, Other 0; pbo: Caucasian 68.0, Black 5.7, Asian 0, Hispanic or Latino 26.2, Pacific Islander 0, Other 0 | not reported |
| Kadowaki T 2013 | sita 50mg QD | 52 weeks | sita 50mg: 76; pbo: 71 | not reported | not reported | Japan | Japanese | overall 7.5 |
| Kim MK  2015 | teneli 20mg QD | 16 weeks | teneli: 136; pbo: 68 | teneli: 55.7 (8.7); pbo: 56.4 (9.2) | teneli: 55.1; pbo: 50.0 | Korea | Korean | teneli: 6.7 (4.8); pbo: 8.0 (5.9) |
| Nauck MA 2009 | alo 12.5mg QD; alo 25mg QD | 26 weeks | alo 12.5mg QD: 213; 25mg QD: 210; pbo: 104 | alo 12.5mg QD: 55 (11); 25mg QD: 54 (11); pbo 56 (11) | alo 12.5mg QD: 47.4; 25mg QD: 54.3; pbo: 48 | 15 countries; not reported | [RACE]: alo 12.5mg QD: White 80, African American 2, Asian 8, Other race 10; 25mg QD: White 76, African American 6, Asian 9, Other race 9; pbo: White 76, African American 7, Asian 6, Other race 11; [ETHNICITY]: alo 12.5: Hispanic or Latino 31, Not Hispanic or Latino 69; alo 25: Hispanic or Latino 32, Not Hispanic or Latino 68; pbo: Hispanic or Latino 24, Not Hispanic or Latino 76 | alo 12.5mg QD: 6 (5); 25mg QD: 6 (4); pbo: 6 (5) |
| Odawara M 2014 | vilda 50mg BID | 12 weeks | vilda 50mg BID: 69; pbo: 70 | vilda 50mg BID: 58.7 (9.81); pbo: 57.5 (9.15) | vilda 50mg BID: 63.8; pbo: 68.6 | Japan | Japanese | vilda 50mg BID: 7.2 (6.18); pbo: 7.0 (5.92) |
| Pan C 2012 | vilda 50mg BID; vilda 50mg QD | 24 weeks | vilda 50mg BID: 146; 50mg QD: 148; pbo: 144 | vilda 50mg BID: 54.2 (9.62); 50mg QD: 53.7 (10.0); pbo: 54.5 (9.68) | vilda 50mg BID: 50; 50mg QD: 44.6; pbo: 45.8 | China | Chinese | vilda 50mg BID: 4.92 (4.8); 50mg QD: 5.02 (4.42); pbo: 5.15 (4.58) |
| Pan CY 2017 | alo 25mg QD | 16 weeks | alo 25mg QD: 92; pbo: 92 | alo 25mg QD: 51.6 (10.4); pbo: 53.1 (8.9) | alo 25mg QD: 59.8; pbo: 58.1 | China, Taiwan, Hong Kong | [country or region] alo 25mg: China 97.8, Hong Kong 2.2, Taiwan 0; pbo: China 97.8, Hong Kong 1.1, Taiwan 1.1 | alo 25mg QD: 1.9 (2.4); pbo: 2.1 (2.8) |
| Raz I 2008 | sita 100mg QD | 18 weeks | sita 100mg: 96; pbo: 94 | sita 100mg: 53.6 (9.5); pbo 56.1 (9.5) | sita 100mg: 51; pbo: 41.5 | multinational; not reported | [RACE] sita 100mg: White 42, Hispanic 32, Black 3, Multiracial 22, Other 1; pbo: White 47, Hispanic 25, Black 1, Multiracial 25, Other 2 | sita 100mg: 8.4 (6.5); pbo: 7.3 (5.3) |
| Rosenstock J 2011 | goso 20mg QD; goso 30mg QD | 12 weeks | goso 20mg QD: 116; 30mg QD: 116; pbo: 57 | overall: 56.5 | not reported | not reported | not reported | Mean (Range) goso 20mg QD: 7.1 (0.1 to 25.2); 30mg QD: 8.3 (0.1 to 44.2); pbo: 9.2 (0.2 to 30.2) |
| Rosenstock J 2012 | sita 100mg QD | 12 weeks | sita 100mg: 65; pbo: 65 | sita 100mg: 51.7 (8.1); pbo: 53.3 (7.8) | sita 100mg: 58; pbo: 48 | not reported | not reported | sita 100mg: 5.6 (4.7); pbo: 6.4 (5.0) |
| Ross SA 2012 | lina 2.5mg BID; lina 5mg QD | 12 weeks | lina 2.5mg BID: 223; 5mg QD: 224; pbo 44 | lina 2.5mg BID: 58.7 (9.9); 5mg QD: 58.4 (10.6); pbo: 59.9 (10.7) | lina 2.5mg BID: 61.9; 5mg QD: 54.0; pbo: 47.7 | India, Republic of Korea, Malaysia, Belgium, France, Italy, the Netherlands, Spain, Canada | lina 2.5mg BID: White 67.3, Asian 32.3, Other 0.4; 5mg QD: White 62.1, Asian 36.6, Other 1.3; pbo: White 72.7, Asian 27.3, Other 0 | lina 2.5mg BID: ≤1 year 7.9, > 1-5 years 39.7, > 5 years 52.3; 5mg QD: ≤1 year 9.0, > 1-5 years 38.9, > 5 years 52.0; pbo: ≤1 year 2.3, > 1-5 years 41.9, > 5 years 55.8 |
| Scott R 2008 | sita 100mg QD | 18 weeks | sita 100mg: 94; pbo: 92 | sita 100mg: 55.2 (9.8); pbo: 55.3 (9.3) | sita 100mg: 55; pbo: 59 | multinational; not reported | sita 100mg: Caucasian 61, Asian 38, Others 1; pbo: Caucasian 61, Asian 39, Others 0 | sita 100mg: 4.9 (3.5); pbo: 5.4 (3.7) |
| Seino Y  2012 | alo 12.5mg QD; alo 25mg QD | 12 weeks | alo 12.5mg: 92; alo 25mg 96; pbo: 100 | alo 12.5mg: 53.4 (8.8); alo 25mg: 52.3 (8.02); pbo: 52.1 (8.05) | alo 12.5mg: 65.2; alo 25mg: 68.8; pbo: 72.0 | Japan | Japanese | alo 12.5mg: 6.34 (5.39); alo 25mg: 6.62 (4.80); pbo: 6.04 (4.36) |
| Shankar RR 2017 | ertu 25mg QW | 24 weeks | omari 25mg QW: 201; pbo: 201 | omari 25mg QW: 57.5 (8.1); pbo: 56.8 (9.1) | omari 25mg QW: 50.2; pbo: 50.7 | not reported | omari 25mg QW: White 86.1, Asian 2.5, Black 9.5, Multi-racial 1.0, Native American 1.0; pbo: White 86.1, Asian 2.5, Black 8.0, Multi-racial 0, Native American 3.5 | omari 25mg QW: 8.2 (5.2); pbo: 7.4 (5.6) |
| Taskinen MR 2011 | lina 5mg QD | 24 weeks | lina 5mg QD: 523; pbo: 177 | lina 5mg QD: 56.5 (10.1); pbo: 56.6 (10.9) | lina 5mg QD: 53; pbo: 57 | Czech Republic, Finland, Greece, India, Israel, Mexico, New Zealand, Russia, Sweden, USA | lina 5mg QD: White 75, Asian 22, Other 3; pbo: White 79, Asian 18, Other 3 | lina 5mg QD: ≤ 1 year: 11, > 1-5 years: 34, > 5 years: 56; pbo: ≤ 1 year: 13, > 1-5 years: 34, > 5 years: 53 |
| Terra SG 2011 | goso 2mg QD; goso 5mg QD; goso 10mg QD; goso 20mg QD | 12 weeks | goso 2mg QD: 37; 5mg QD: 38; 10mg QD: 77; 20mg QD: 73; pbo: 76 | goso 2mg QD: 55.9 (9.0); 5mg QD: 56.4 (6.5); 10mg QD: 55.7 (8.4); 20mg QD: 55.9 (8.1); pbo: 57.2 (7.8) | goso 2mg QD: 67.6; 5mg QD: 73.7; 10mg QD: 59.7; 20mg QD: 65.8; pbo: 69.7 | Colombia, Germany, Italy, Spain, Sweden, USA | goso 2mg QD: White 64.9, Black 8.1, Asian 0, Other 27.0, Unspecified 0; 5mg QD: White 57.9, Black 13.2, Asian 0, Other 28.9, Unspecified 0; 10mg QD: White 59.7, Black 6.5, Asian 3.9, Other 29.9, Unspecified 0; 20mg QD: White 65.8, Black 11.0, Asian 0, Other 23.3, Unspecified 0; pbo: White 59.2, Black 11.8, Asian 0, Other 27.6, Unspecified 1.3 | goso 2mg QD: 9.4; 5mg QD: 6.8; 10mg QD: 6.7; 20mg QD: 6.5; pbo: 7.2 (SD n/a) |
| Wang W 2016 | lina 5mg QD | 24 weeks | lina 5mg: 205; pbo: 100 | lina 5mg: 55.1 (10.7); pbo: 56.5 (8.7) | lina 5mg: 49.8; pbo: 50.0 | China, Philippines, Malaysia | [Country] lina 5mg: China 89.8, Malaysia 6.3, Philippines 3.9; pbo: China 80.0, Malaysia 11.0, Philippines 9.0 | lina 5mg: ≤1 year 16.3, > 1-5 40.9, > 5 42.9; pbo: ≤1 year 19.6, > 1-5 33.0, > 5 47.4 |
| White JL 2014 | saxa 2.5mg BID | 12 weeks | saxa 2.5mg BID: 74; pbo: 86 | saxa 2.5mg BID: 53.9 (10.35); pbo: 56.6 (9.97) | saxa 2.5mg BID: 54.1; pbo: 52.3 | USA, Germany, Hungary, Puerto Rico | [RACE] saxa 2.5mg BID: White 86.5, Black/African American 10.8, American Indian or Alaska native 0, Asian 2.7. [ETHNICITY] hispanic or latino 39.2, non-hispanic or latino 45.9, not reported 14.9; pbo: White 93.0, Black/African American 3.5, American Indian or Alaska native 1.2, Asian 2.3. [ETHNICITY] hispanic or latino 40.7, non-hispanic or latino 41.9, not reported 17.4 | saxa 2.5mg BID: 5.8 (6.37); pbo: 6.2 (4.21) |
| Yang W 2011 | saxa 5mg QD | 24 weeks | saxa 5mg: 283; pbo: 287 | saxa 5mg: 53.8 (10.4); pbo: 54.4 (10.1) | saxa 5mg: 48.1; pbo: 48.4 | China, India, South Korea | [REGION] saxa 5mg: China 58.3, India 25.8, South Korea 15.9; pbo: China 565.1, India 25.8, South Korea 18.1 | saxa 5mg: 5.1 (5.0); pbo: 5.1 (4.0) |
| Yang W 2012 | sita 100mg QD | 24 weeks | sita 100mg: 197; pbo: 198 | sita 100mg: 54.1 (9.0); pbo: 55.1 (9.8) | sita 100mg: 47; pbo: 55 | China | Chinese | sita 100mg: 6.4 (4.4); pbo: 7.3 (4.6) |

**Characteristics of Included Studies – DPP4i Added to Metformin Background continued**

| **Study ID** | **Baseline A1C % (SD)** | **Mean Change in HbA1C % vs. baseline** | **Mean Difference in HbA1c % vs pbo** | **HYPO def** | **Ascertainment of Hypo** | **Rescue Medication** | **Excl of Pts w events? At screening or during study)** |
| --- | --- | --- | --- | --- | --- | --- | --- |
| Ahrén B  2004 | vilda 50mg QD: 7.7 (0.6); pbo: 7.8 (0.7) | vilda 50mg QD: -0.6 (0.1); pbo: 0.1 (0.1)  Adjusted mean change | -0.7 (0.1) | Hypoglycemia was defined as symptoms  suggestive of hypoglycemia accompanied  by a self-monitoring of blood glucose  (SMBG) measurement of < 3.1 mmol/l  plasma glucose equivalents. | Patients were provided with glucose monitoring devices and supplies and instructed  on their use. | not reported | No. However, during screening, patients were excluded if they had severe diabetes complications. |
| Amin NB 2015 | sita 100mg: 8.24 (0.15); pbo: 8.08 (0.14) | [LSM pbo corrected, 80% CI] sita 100mg; -0.87 (-1.01 to -0.73); pbo: -0.11 (-0.25 to 0.04) | [LSM] manually calculated sita 100mg; -0.76 | Hypoglycaemia was captured as an AE based on signs/symptoms, home blood glucose monitoring [defined as blood glucose ≤3.9mmol/l (70mg/dl) using an ACCU-CHEK home glucometer, or ≤4.1mmol/l (74mg/dl). Severe not defined. | Home blood glucose monitoring using an ACCU-CHEK  home glucometer, or ≤4.1mmol/l (74mg/dl) using International  Federation of Clinical Chemistry-referenced ACCU-CHEK glucometers; The recommended home glucose monitoring frequency was determined for each subject by the investigator; however, a frequency of at least once daily (and in event of hypoglycaemia)  was emphasized. | Rescue therapy not permitted. Patients requiring rescue were withdrawn from study. | no |
| Bosi E 2007 | vilda 50mg QD: 8.4 (0.9); 100mg QD: 8.4 (1.0); pbo 8.3 (0.9) | vilda 50mg QD: -0.5 (0.1); 100mg QD: -0.9 (0.1); pbo: 0.2 (0.1) | vilda adjusted mean change; between-treatment difference  (vildagliptin - placebo) vilda 50mg QD: -0.7 (0.1) (p<0.001); 100mg QD: -1.1 (0.1) (p<0.001) | Hypoglycemia was defined as symptoms suggestive of low blood glucose confirmed by self-monitored blood glucose measurement <3.1 mmol/l plasma glucose equivalent. Severe hypoglycemia was defined as any episode requiring the assistance of another party. | Patients were provided with glucose monitoring devices and supplies and instructed on their use. | not reported | no |
| Bryson A  2016 | teneli  5mg: 7.96 ;10mg: 7.78; 20mg: 7.96; 40mg: 7.87; pbo: 7.88 | teneli  5mg: -0.58 (SE 0.07); 10mg: -0.68 (0.07); 20mg: -0.76 (0.07); 40mg: -0.91 (0.07); pbo: -0.28 (0.07) | teneli  5mg: -0.30 (95% CI -0.49 to -0.11); 10mg: -0.40 (-0.59 to -0.21); 20mg: -0.48 (-0.67 to -0.29); 40mg: -0.63 (-0.82 to -0.44) | Major: symptomatic requiring assistance with BG <3.0mmol/L. Minor: either symptomatic with BG <3.0mmol/L and no need for assistance or asymptomatic with BG <3.0mmol/L and episodes suggestive of hypoglycemia without BG measurements. | BG confirmations | Patients with hyperglycemia were withdrawn. | Patients with repeated minor or major hypoglycemia did not continue into the open-label extension. |
| Charbonnel B 2006 | sita 100mg: 8.0 (0.8); pbo: 8.0 (0.8) | [LSM pbo subtracted] (95% CI): sita -0.67 (-0.77 to -0.57); pbo: -0.02 (-0.15 to 0.10) | [LSM pbo subtracted] (manually calculated): sita -0.65 | not reported | Investigators evaluated each clinical adverse experience for intensity  (mild, moderate, or severe), duration, outcome, and relationship to study drug. | Rescue therapy permitted. Data exclude after rescue. | No. A  history of hypoglycemia was not an exclusion  criterion. |
| DeFronzo RA 2009 | saxa 2.5mg QD: 8.1 (1.0); 5mg QD: 8.1 (0.8); 10mg QD: 8.0 (1.0); pbo: 8.1 (0.9) | [LSM] adjusted (SE) saxa 2.5mg QD: -0.59 (0.07); 5mg QD: -0.69 (0.07); 10mg QD: -0.58 (0.07); pbo: 0.13 (0.07) | [LSM] adjusted (SE) saxa 2.5mg QD: -0.73 (0.10) (-0.92 to -0.53); 5mg QD: -0.83 (0.10) (-1.02 to -0.63); 10mg QD: -0.72 (0.10) (-0.91 to -0.52) | Reported provided in Table 2 but not defined. Confirmed hypoglycemia defined by symptoms of hypoglycemia in the setting of a fingerstick blood glucose value ≤50 mg/dl (2.8 mmol/l). | Patient reported hypoglycemia  adverse events, AND confirmed  hypoglycemia (fingerstick glucose  value of 50 mg/dl associated with symptoms),  were recorded. | Rescue therapy permitted. Data exclude after rescue. | no |
| Derosa G  2012 | sita 100mg: 8.1 (0.8); pbo: 8.0 (0.7) | sita 100mg: -1.4; pbo: -0.7  (manually calculated) | sita 100mg: -0.7 (manually calculated)  (p <0.05) | Hypoglycemia defined as FPG <3.3mmol/L. | BG confirmed. | not reported | not reported |
| Derosa G  2012 | vilda 50mg: 8.1 (0.6); pbo: 8.2 (0.7) | vilda 50mg: -1.2; pbo: -0.8 (manually calculated) | vilda 50mg: -0.4  (manually calculated)  (p <0.05) | Hypoglycemia defined as FPG <3.3mmol/L. | BG confirmed | not reported | not reported |
| Forst T 2010 | lina 1mg QD: 8.2 (0.7); 5mg QD: 8.5 (0.8); 10mg QD: 8.4 (0.7); pbo: 8.4 (0.7) | mean change from baseline (SD) (FAS LOCF) lina 1mg QD: -0.14 (0.92); 5mg QD: -0.50 (0.81); 10mg QD: -0.42 (0.87); pbo: 0.24 (0.74) | manually calculated lina 1mg QD: -0.38; 5mg QD: -0.74; 10mg QD: -0.66 | not reported | During the study, patients made visits to the study center at weeks 2, 4, 8 and 12, with a follow-up visit at week 14. Patients underwent safety and efficacy assessments and were withdrawn if they had a fasting plasma glucose > 13.3 mmol ⁄ l (measured on two separate days) at any visit; if they showed clinical signs of severe hypoglycaemia or a blood glucose level < 2.5 mmol⁄ l; if their dose of metformin changed. | not reported | Yes, if severe. |
| Goodman M 2009 | vilda 100mg QD AM: 8.5 (1); 100mg QD PM: 8.5 (0.9); pbo: 8.7 (1.1) | (SE) vilda 100mg QD AM: -0.66 (0.11); 100mg QD PM: -0.53 (0.11); pbo: 0.17 (0.11) | vilda 100mg QD AM: -0.83 (p<0.001); 100mg QD PM: -0.70 (p<0.001) (no sd) | Hypoglycaemia defined as a plasma glucose <3.1 mmol/l. Severe hypoglycaemia is any event where the patient required assistance from a third party or hospitalization. | Patients were educated about hypoglycemic symptoms and treatment of hypoglycemic events, and were instructed to take a blood glucose measurement if a hypoglycemic event was suspected | not reported | no |
| Kadowaki T 2013 | overall 8.3 | [LSM] sita 50 mg: -0.4 (-0.6 to -0.2); pbo: 0.3 (0.1 to 0.5) | [LSM] sita 50mg: -0.70 (-0.9 to -0.5) | not reported | no | not reported | no |
| Kim MK  2015 | teneli: 7.79 (0.8); pbo: 7.72 (0.65) | teneli: -0.87 (0.65); pbo: -0.06 (0.55) | -0.78 (SE 0.09) (95% CI -0.95 to -0.61)  adjusted mean for difference | not reported | not reported | Rescue therapy was not permitted. | no |
| Nauck MA 2009 | alo 12.5mg QD: 7.9 (0.7); 25mg QD: 7.9 (0.8); pbo: 8.0 (0.9) | [LSM] [SE] alo 12.5mg QD: -0.6 (0.1); 25mg QD: -0.6 (0.1); pbo: -0.1 (0.1) | [LSM] manually calculated: alo 12.5mg: -0.5; alo 25mg: -0.5 | Hypoglycaemia [blood glucose <60 mg ⁄dl (<3.3 mmol⁄l) in presence of symptoms; blood glucose <50 mg⁄dl (<2.8 mmol⁄l) regardless of symptoms] and severe hypoglycaemia [defined as any episode requiring the assistance of another person to administer actively, carbohydrate, glucagon or other resuscitative actions, associated with blood glucose <60 mg⁄dl (< 3.3 mmol⁄l)]. | Patient recorded; Visits included assessment of vital signs, physical examination, concomitant medication review and adverse event (AE) monitoring, review of diaries and glucometer readings, laboratory assessments (hematology, serum chemistry  and urinalysis) and documentation of drug dosing compliance, as determined by via pill count. | Patients requiring rescue therapy were terminated from study. | no |
| Odawara M 2014 | vilda 50mg BID: 8 (0.83); pbo: 8.0 (0.96) | adjusted mean change (SE) vilda 50mg BID: =-1.1 (0.06); pbo: -0.1 (0.06) | difference in adjusted mean change vilda 50 mg BID: -1.0 (0.09) (p<0.001) | Hypoglycemia was defined as symptoms suggestive of hypoglycemia that was further confirmed by a self-monitored blood glucose measurement of <3.1 mmol/L.  The event was considered grade 1 if the patient was able to initiate self-treatment, and grade 2 if the patient required assistance of another person or hospitalization. | Patient-reported; confirmed by blood glucose measurement | Rescue medication (additional OADs or  insulin) not permitted. | no |
| Pan C 2012 | vilda 50mg BID: 8.09 (0.85); 50mg QD: 8.05 (0.84); pbo: 8.01 (0.82) | adjusted mean change (SE) vilda 50mg BID: -1.05 (0.08); 50mg QD: -0.92 (0.08); pbo: -0.54 (0.08) | vilda 50mg BID: -0.51 (0.11) (p<0.001); not reported for vilda 50 qd because not significant between-treatment difference (SE). | Hypoglycaemia was defined as the presence of symptoms suggestive of hypoglycaemia, confirmed by self-monitored glucose <56 mg/dl (3.1 mmol/l).  Severe hypoglycaemia was defined as an episode requiring assistance of another party. | All  patients were provided with one glucose meter each for self-monitored blood glucose and were instructed about the use of  the device at the entry of the study; Hypoglycaemia was defined  as the presence of symptoms suggestive of hypoglycaemia,  confirmed by self-monitored glucose <56 mg/dl (3.1 mmol/l).  Severe hypoglycaemia was defined as an episode requiring  assistance of another party. Patients were required to record the event and associated information such as glucose value and time of occurrence in the study diary. | not reported | no |
| Pan CY 2017 | alo 25mg QD: 8.04 (0.92); pbo: 7.86 (0.78) | [LSM] alo 25mg QD: =-0.99; pbo: -0.42 | [LSM] alo 25mg QD: -0.58 (-0.78 to -0.37) (p<0.001) | Mild-to-moderate hypoglycemia,  whether symptomatic or asymptomatic, was  defined as plasma glucose levels <3.9mmol/L. Severe hypoglycemia was defined as any hypoglycemic episode that required the assistance of another person to actively  administer carbohydrate, glucagon, or other resuscitative actions, and was associated with a documented plasma glucose level <3.9 mmol/L. | not reported | Rescue permitted. | no |
| Raz I 2008 | sita 100mg: 9.3 (0.9); pbo: 9.1 (0.8) | [LSM] sita 100mg: -1.0 (-1.2 to -0.8); pbo: 0.0 (-0.2 to 0.3) | [LSM] sita 100mg: -1.0 (-1.4 to -0.7) | not reported | not reported | Rescue permitted but not included. | no |
| Rosenstock J 2011 | goso 20mg QD: 8.24 (1.25); 30mg QD: 8.17 (1.39); pbo: 8.05 (1.02) | [LSM] goso 20mg QD: -0.92 (-1.09 to -0.75); 30mg QD: -1.04 (-1.22 to -0.87); pbo: -0.13 (-0.38 to 0.13) | [LSM] goso 20mg QD: -0.79 (-1.10 to -0.49); 30mg QD: -0.92 (-1.23 to -0.61) | not reported | Laboratory testing for safety parameters was carried out at  baseline and weeks 2, 4, 8 and 12, and adverse events were  recorded at all visits. | not reported | no |
| Rosenstock J 2012 | sita 100mg: 7.64 (0.95); pbo: 7.75 (0.83) | sita 100mg: -0.74; pbo: -0.22 | sita 100mg: -0.52 | not reported | no, patient-reported | not reported | no |
| Ross SA 2012 | lina 2.5mg BID: 7.96 (0.78); 5mg QD: 7.98 (0.72); pbo: 7.92 (0.74) | lina 2.5mg BID: -0.46 (0.05); 5mg QD: -0.52 (0.05); pbo: 0.28 (0.11) (SE) | lina 2.5mg BID: -0.74 (-0.97 to -0.52); 5mg QD: -0.80 (-1.02 to -0.58) | Hypoglycaemia episodes were classified by investigators  according to guidelines from the American Diabetes  Association. | not reported | Rescue permitted but not included. | no |
| Scott R 2008 | sita 100mg: 7.8 (1.0); pbo: 7.7 (0.9) | [LSM] sita 100mg: -0.73 (-0.87 to -0.60); pbo: -0.22 (-0.36 to -0.08) | [LSM] sita 100mg: -0.51 (-0.70 to -0.32) (p ≤ 0.001) | not reported | not reported | not reported | no |
| Seino Y  2012 | alo 12.5mg: 7.89 (0.82); alo 25mg: 8.02 (0.73); pbo: 8.00 (0.86) | alo 12.5mg: -0.55 (SE 0.058); alo 25mg: -0.64 (0.056); pbo: 0.22 (0.055) | alo 12.5mg: -0.77; alo 25mg: -0.86  manually calculated | not reported | not reported | not reported | no |
| Shankar RR 2017 | omari 25mg QW: 8.1 (0.9); pbo: 8.0 (0.9) | [LSM] omari 25mg QW: -0.54 (-0.69 to -0.40); pbo: 0.0 (-0.14 to 0.15) | [LSM] omari 25mg QW: -0.55 (-0.75 to -0.34) | Symptomatic hypoglycemia: episode with clinical symptoms attributed to hypoglycemia, without regard to glucose level.  Severe hypoglycemia: episode that required assistance, either medical or non-medical. Episodes with a markedly depressed level of consciousness, a loss of consciousness, or seizure were classified as having required medical assistance, whether or not medical assistance was obtained. Asymptomatic hypoglycemia: glucose values ≤3.9 mmol/L without symptoms. | A standard questionnaire was provided to subjects to  collect hypoglycemia information. | Rescue permitted but data not included. | no |
| Taskinen MR 2011 | lina 5mg QD: 8.09 (0.86); pbo: 8.02 (0.88) | lina 5mg QD: =-0.49 (0.04); pbo: 0.15 (0.06) | lina 5mg QD: -0.64 (0.07) (-0.78 to -0.50) (p<0.0001) adjusted mean change from baseline | Plasma glucose concentration ≤3.9 mmol/l | HbA1c, fasting plasma glucose (FPG), adverse events (AEs),  serious adverse events (SAEs) and vital signs were evaluated at  every visit; Safety assessments were made at screening,  placebo run-in and weeks 0, 12, 24 and 25. | Rescue confounding | no |
| Terra SG 2011 | goso 2mg QD: 8.00 (0.88); 5mg QD: 8.52 (1.30); 10mg QD: 8.37 (1.08); 20mg QD: 8.33 (0.97); pbo: 8.38 (1.19) | [LSM] goso 2mg QD: -0.26 (-0.58 to 0.06); 5mg QD: -0.69 (-1.00 to -0.39); 10mg QD: -0.65 (-0.87 to -0.42); 20mg QD: -0.70 (-0.93 to -0.48); pbo: 0.05 (-0.17 to 0.27) (PPS) | [LSM] goso 2mg QD: -0.31 (-0.70 to 0.08); 5mg QD: -0.74 (-1.12 to -0.36); 10mg QD: -0.70 (-1.02 to -0.38); 20mg QD: -0.75 (-1.07 to -0.43) (PPS) | not reported | not reported | not reported | no |
| Wang W 2016 | lina 5mg: 7.99 (0.83); pbo: 8.00 (0.80) | lina 5mg: -0.66 (0.05); pbo: -0.14 (0.07) Adjusted mean (SE) | lina 5mg: -0.52 (0.09) (-0.70 to -0.34) Adjusted mean (SE) | Investigator-defined hypoglycemia was categorized as asymptomatic with plasma glucose ≤70 mg/dL (3.89 mmol/L),  documented symptomatic with glucose 54–70 mg/dL (3.0-3.89 mmol/L), documented symptomatic with glucose <54 mg/dL (3.0 mmol/L) without need for external assistance, and severe (requiring another person’s assistance for active administration of resuscitative actions). | not reported | Rescue medication (glimepiride) permitted. | no |
| White JL 2014 | saxa 2.5mg BID: 7.92 (0.961); pbo: 7.97 (0.819) | [LSM] (SE) saxa 2.5mg BID: -0.56 (0.09); pbo: -0.22 (0.08) | saxa 2.5mg BID: -0.32 (SE 0.12) p=0.006 | Confirmed hypoglycemia, defined by hypoglycemic symptoms plus a fingerstick glucose value ≤50 mg/dL (2.8 mmol/L). | No; confirmed hypoglycemia, defined by hypoglycemic symptoms  plus, a fingerstick glucose value ≤50 mg/dL. | not reported | no |
| Yang W 2011 | saxa 5mg: 7.9 (0.8); pbo: 7.9 (0.8) | saxa 5mg: -0.78; pbo: -0.37 | saxa 5mg: adjusted mean change from baseline -0.42 (-0.55 to -0.29) | Reported presented but not defined. Confirmed hypoglycemic events were those associated with symptoms of hypoglycemia and a documented plasma glucose level ≤2.8 mmol/L, and reported hypoglycemic events were defined as signs and symptoms consistent with hypoglycemia with or without documented glucose measurement | No, signs and symptoms are patient-reported | Rescue therapy was not permitted during the study. | no |
| Yang W 2012 | sita 100mg: 8.5 (0.9); pbo: 8.5 (0.9) | [LSM] sita 100mg: -1.0 (1.2 to -0.9); pbo: -0.1 (-0.3 to 0) | [LSM] sita 100mg: -0.9 (-1.1 to -0.7) | All hypoglycemic events reported in the present study were symptomatic (i.e. episode with clinical symptoms attributed to hypoglycemia, without regard to glucose level - symptoms of hypoglycemia, such as sweating, anxiety, and palpitations); marked severity (e.g. markedly depressed level of consciousness loss of consciousness, or seizures). Patients were instructed to also report episodes of asymptomatic low blood glucose levels (i.e. ≤3.9 mmol⁄L). | Patients were counseled to self-monitor their blood  glucose levels and immediately notify investigators if  they experienced symptoms of hypoglycemia, such as  sweating, anxiety, and palpitations, for assessment of hypoglycemic events during the study. Hypoglycemia was assessed by the study site investigators by reviewing patient self-reports of signs and symptoms of hypoglycemia. A fingerstick blood glucose determination was not required to support the documentation of a  symptomatic episode of hypoglycemia, but was  required to report an episode of asymptomatic hypoglycemia.  Patients were instructed to also report episodes of asymptomatic low blood glucose levels (i.e.  ≤3.9 mmol⁄ L). | Rescue therapy with glipizide permitted. Data obtained after the initiation of rescue  therapy was treated as missing in all analyses. | no |

**Characteristics of Included Studies – GLP1RA Added to Metformin Background**

| **Study ID** | **Dose** | **Study Duration** | **n=** | **Mean Age (SD)** | **Gender (% male)** | **Countries Studied** | **Ethnicity (%)** | **Duration of Diabetes in Years (SD)** |
| --- | --- | --- | --- | --- | --- | --- | --- | --- |
| Ahrén B 2013 | lixi 20μg QD SC AM; lixi 20μg QD SC PM | 24 weeks | lixi 20μg AM: 255; 20μg PM: 255; pbo: 170 | lixi 20μg AM: 54.5; 20μg PM: 54.8; pbo: 55.0 (9.4) | lixi 20μg AM: 38.4; 20μg PM: 44.7; pbo: 47.6 | Australia, Canada (LMC), Chile, Czech Republic, Germany, Croatia, Mexico, Morocoo, the Philippines, Romania, Russian Federation, South Africa, Spain, Ukraine, USA, Venezuela | lixi 20μg AM: Caucasian 86.7, Black 2.7, Asian 8.6, Other 2.0; 20μg PM: Caucasian 89.4, Black 2.4, Asian 7.8, Other 0.4; pbo: Caucasian 91.2, Black 2.4, Asian 6.5, Other 0 | lixi 20μg AM: 6.2 (5.3); 20μg PM: 6.2 (5.4); pbo: 5.9 (4.7) |
| Bolli GB 2014 | lixi one-step dose  increase (10 μg once daily for 2 weeks then 20 μg QD; lixi two-step dose increase (10 μg once  daily for 1 week, 15 μg once daily for 1 week then 20 μg QD | 24 weeks (main efficacy)  76 weeks (safety) | lixi one-step: 161; two-step: 161; pbo: 160 | lixi one-step: 55.4 (8.9); two-step: 54.6 (8.9); pbo: 58.2 (9.8) | lixi one-step: 44; two-step: 45; pbo:45 | 15 countries; not specified | lixi one-step: Caucasian 88, Black 1, Asian 8, Other 4; two-step: Caucasian 91, Black 1, Asian 7, Other 1; pbo: Caucasian 93, Black 1, Asian 6, Other 1 | lixi one-step: 5.8 (3.9); two-step: 6.0 (4.6); pbo: 6.2 (4.7) |
| Davies M 2017 | sema 2.5mg PO QD; sema 5mg PO QD; sema 10mg PO QD; sema 20mg PO QD; sema 40mg PO QD; sema 1mg SC QW | 26 weeks | sema 2.5mg PO QD: 70  5mg PO QD: 70  10mg PO QD: 69  20mg PO QD: 70  40mg PO QD: 71  40mg PO QD slow dose (8w) escalation: 70  40mg PO QD fast dose (2w) escalation: 70  pbo: 71 | sema 2.5mg PO QD: 56.7 (9.9) 5mg PO QD: 55.7 (11.0) 10mg PO QD: 56.5 (10.1) 20mg PO QD: 58.3 (10.4)  40mg PO QD: 56.5 (10.2) 40mg PO QD slow dose (8w) escalation: 57.1 (10.5) 40mg PO QD fast dose (2w) escalation: 57.7 (10.8) pbo: 58.9 (10.3) | sema 2.5mg PO QD: 64.3  5mg PO QD: 67.1  10mg PO QD: 62.3  20mg PO QD: 62.9  40mg PO QD: 60.6  40mg PO QD slow dose (8w) escalation: 58.6  40mg PO QD fast dose (2w) escalation: 62.9  pbo: 56.3 | Austria, Bulgaria, Canada, Denmark, Germany, Israel, Italy, Malaysia, Serbia, South Africa, Spain, Sweden, United Kingdom, USA | sema 2.5mg PO QD: White 81.4, Black or African American 8.6, Asian 10.0, American Indian or Alaska Native 0, Other 0 5mg PO QD: White 90.0, Black or African American 2.9, Asian 5.7, American Indian or Alaska Native 0, Other 1.4 10mg PO QD: White 82.6, Black or African American 10.1, Asian 5.8, American Indian or Alaska Native 0, Other 1.4 20mg PO QD: White 84.3, Black or African American 5.7, Asian 5.7, American Indian or Alaska Native 2.9, Other 1.4 40mg PO QD: White 88.7, Black or African American 5.6, Asian 4.2, American Indian or Alaska Native 0, Other 1.4 40mg PO QD slow dose (8w) escalation: White 77.1, Black or African American 10.0, Asian 10.0, American Indian or Alaska Native 0, Other 2.9 40mg PO QD fast dose (2w) escalation: White 84.3, Black or African American 10.0, Asian 5.7, American Indian or Alaska Native 0, Other 0 pbo: White 80.3, Black or African American 8.5, Asian 9.9, American Indian or Alaska Native 1.4, Other 0 | sema 2.5mg PO QD: 6.1 (6.0) 5mg PO QD: 5.3 (4.7) 10mg PO QD: 5.8 (4.8) 20mg PO QD: 7.0 (5.3) 40mg PO QD: 7.7 (5.9) 40mg PO QD slow dose (8w) escalation: 6.6 (4.9) 40mg PO QD fast dose (2w) escalation: 5.6 (4.7) pbo: 6.7 (5.1) |
| DeFronzo RA 2005 | exen 5μg SC BID; exen 10μg SC BID | 30 weeks | exen 5μg BID: 110; 10μg BID: 113; pbo: 113 | exen 5μg BID: 53 (11); 10μg BID: 52 (11); pbo: 54 (9) | exen 5μg BID: 51.8; 10μg BID: 60.2; pbo: 59.3 | USA | exen 5μg BID: Caucasian 77.3, Black 10.9, Hispanic 7.3, Other 4.6; 10μg BID: Caucasian 79.6, Black 8.8, Hispanic 8.0, Other 3.5; pbo: Caucasian 72.6, Black 13.3, Hispanic 10.6, Other 3.5 | exen 5μg BID: 6.2 (5.9); 10μg BID: 4.9 (4.7); pbo: 6.6 (6.1) |
| Derosa G  2013 | exen 10μg BID | 52 weeks | exen 10μg BID: 86; pbo: 85 | exen 10μg BID: 57.3 (7.7); pbo: 56.7 (7.3) | exen 10μg BID: 43; pbo: 41 | Italy | not reported | exen 10μg BID: 0.63; pbo: 0.65 |
| Kim D 2007 | exen LAR 0.8mg SC QW; exen 2.0mg SC QW | 13 weeks | exen 0.8mg LAR: 16; exen LAR 2.0mg: 15; pbo: 14 | exen 0.8mg LAR: 55 (12); exen 2.0mg LAR: 51 (11); pbo: 55 (9) | exen 0.8mg LAR: 75; exen 2.0mgLAR: 67; pbo 36 | not reported; | exen 0.8mg LAR: Caucasian 56, Black 13, Hispanic 19, Other 13; exen 2.0mg LAR: Caucasian 60, Black 20, Hispanic 20, Other 0; pbo: Caucasian 64, Black 14, Hispanic 21, Other 0 | exen 0.8mg LAR: 5 (3); exen 2.0mg LAR: 4 (5); pbo: 4 (4) |
| Lingvay I  2018 | sema 0.05mg QD; sema 0.1mg QD; sema 0.2mg QD; sema 0.3mg; lira 0.3mg QD; lira 0.6mg QD; lira 1.2mg QD; lira 1.8mg QD | 26 weeks | sema 0.05mg: 64, 0.1mg: 63, 0.2mg: 65, 0.3mg: 63; pbo: 129 | sema 0.05mg: 57.5 (9.8), 0.1mg: 57.5 (10.0), 0.2mg: 58.4 (9.6), 0.3mg: 54.8 (9.7); pbo: 57.1 (9.2) | sema 0.05mg: 51.6, 0.1mg: 55.6, 0.2mg: 66.1, 0.3mg: 50.8; pbo: 55.8 | Austria, Canada, Czech Republic, Germany, Malaysia, Russia, Serbia, South Africa, UK, US | sema 0.05mg: Asian 9.4, Black or African American 14.1, White 76.6, Other 0; 0.1mg: Asian 14.3, Black or African American 6.3, White 79.4, Other 0; 0.2mg: Asian 7.7, Black or African American 9.2, White 78.5, Other 4.6; 0.3mg: Asian 11.1, Black or African American 17.5, White 69.8, Other 1.6; pbo: Asian 10.9, Black or African American 8.5, White 79.8, Other 0.8 | sema 0.05mg: 6.5 (4.6), 0.1mg: 8.1 (7.3), 0.2mg: 7.2 (5.7), 0.3mg: 6.5 (4.4); pbo: 7.1 (4.5) |
| Lingvay I  2018 | sema 0.05mg QD; sema 0.1mg QD; sema 0.2mg QD; sema 0.3mg; lira 0.3mg QD; lira 0.6mg QD; lira 1.2mg QD; lira 1.8mg QD | 26 weeks | lira 0.3mg: 64, 0.6mg: 64, 1.2mg: 64, 1.8mg: 65; pbo: 129 | lira 0.3mg: 57.2 (10.8), 0.6mg: 59.5 (9.8), 1.2mg: 53.7 (11.4), 1.8mg: 55.8 (9.2); pbo: 57.1 (9.2) | lira 0.3mg: 45.3, 0.6mg: 50.0, 1.2mg: 53.1, 1.8mg: 50.8; pbo: 55.8 | Austria, Canada, Czech Republic, Germany, Malaysia, Russia, Serbia, South Africa, UK, US | lira 0.3mg: Asian 6.3, Black or African American 6.3, White 82.8, Other 4.7; 0.6mg: Asian 3.1, Black or African American 6.3, White 87.5, Other 3.1; 1.2mg: Asian 6.3, Black or African American 9.4, White 84.4, Other 0; 1.8mg: Asian 16.9, Black or African American 6.2, White 73.8, Other 3.1; pbo: Asian 10.9, Black or African American 8.5, White 79.8, Other 0.8 | lira 0.3mg: 8.1 (7.1), 0.6mg: 6.8 (4.6), 1.2mg: 6.9 (4.9), 1.8mg: 6.6 (5.2); pbo: 7.1 (4.5) |
| Nauck MA 2016 | sema OW  without dose escalation (0.1–0.8 mg); or with dose escalation (E) (0.4 mg steps to  0.8 or 1.6mg E over 1–2 weeks) | 12 weeks | sema QW 0.1mg: 47; SC QW 0.2mg: 44; SC QW 0.4mg: 49; SC QW 0.8mg: 44; SC 0.8mg E: 45; SC QW 1.6mg E: 45; pbo:46 | sema QW 0.1mg 55.2 (10.1); SC QW 0.2mg: 54.7 (10.0); SC QW 0.4mg 53.8 (10.2); SC QW 0.8mg: 55.0 (9.7); SC 0.8mg E: 55.9 (7.9); SC QW 1.6mg E: 56.4 (10.5); pbo: 55.3 (10.6) | sema QW 0.1mg: 66; SC QW 0.2mg: 70; SC QW 0.4mg: 77; SC QW 0.8mg: 52; SC 0.8mg E 63; SC QW 1.6mg E: 55; pbo: 61 | Austria, Bulgaria, Finland, France, Germany, Hungary, India, Italy, Serbia, South Africa, Spain, Switzerland, Turkey, United Kingdom | White 75% (breakdown n/a) | sema QW 0.1mg: 3.6 (5.0); SC QW 0.2mg: 2.3 (2.7); SC QW 0.4mg: 2.0 (2.3); SC QW 0.8mg: 3.0 (3.0); SC 0.8mg E: 2.6 (2.1); SC QW 1.6mg E: 1.8 (2.0); pbo: 2.4 (3.3) |
| Ratner RE 2010 | [within 1h of breakfast] lixi 5μg SC QD; lixi 10μg SC QD; lixi 20μg SC QD; lixi 30μg SC QD  lixi 5μg SC BID; lixi 10μg SC BID; lixi 20μg SC BID; lixi 30μg SC BID | 13 weeks | lixi 5μg SC QD: 55; 10μg SC QD: 52; 20μg SC QD: 55; 30μg SC QD: 54  5μg SC BID: 53; 10μg SC BID: 56; 20μg SC BID: 54; 30μg SC BID: 54; pbo 109 | lixi 5μg SC QD: 56.8 (7.8); 10μg SC QD: 55.4 (9.2); 20μg SC QD: 55.4 (9.9); 30μg SC QD: 56.5 (8.7)  5μg SC BID: 57.1 (8.2); 10μg SC BID: 56.0 (7.9); 20μg SC BID: 56.7 (8.3); 30μg SC BID: 55.3 (9.1); pbo: 56.3 (9.2) | lixi 5μg SC QD: 47.3; 10μg SC QD: 59.6; 20μg SC QD: 50.9; 30μg SC QD: 50 5μg SC BID: 47.2; 10μg SC BID: 51.8; 20μg SC BID: 37.0; 30μg SC BID: 42.6; pbo: 56.0 | multinational; not specified | lixi 5μg SC QD: Caucasian 69.1, Black 9.1, Other 21.8; 10μg SC QD: Caucasian 69.2, Black 11.5, Other 19.3; 20μg SC QD: Caucasian 81.8, Black 1.8, Other 16.4; 30μg SC QD: Caucasian 79.6, Black 11.1, Other 9.3; 5μg SC BID: Caucasian 86.8, Black 3.8, Other 9.4; 10μg SC BID: Caucasian 80.4, Black 3.6, Other 16.0; 20μg SC BID: Caucasian 77.8, Black 3.7, Other 18.5; 30μg SC BID: Caucasian 64.8, Black 16.7, Other 18.5; pbo: Caucasian 77.1, Black 11.0, Other 11.9 | lixi 5μg SC QD: 7.2 (4.9); 10μg SC QD: 6.2 (4.1); 20μg SC QD: 6.4 (6.8); 30μg SC QD: 6.0 (4.8) 5μg SC BID: 6.2 (6.0); 10μg SC BID: 6.4 (5.0); 20μg SC BID: 6.6 (5.1); 30μg SC BID: 7.0 (5.4); pbo: 7.1 (5.4) |
| Rosenstock J 2009 B | albi 4mg SC QW; albi 15mg SC QW; albi 30mg SC QW  albi 15mg SC biweekly; albi 30mg SC biweekly; albi 50mg SC biweekly  albi 50mg SC monthly; albi 100mg SC monthly | 16 weeks | albi 4mg SC QW: 34; 15mg SC QW: 34; 30mg SC QW: 29  15mg SC biweekly:30; 30mg SC biweekly: 32; 50mg SC biweekly: 34  50mg SC monthly: 35; 100mg SC monthly: 33; pbo: 50 | albi 4mg SC QW: 50.4 (10.3); 15mg SC QW: 55.5 (10.5); 30mg SC QW: 54.2 (9.7)  15mg SC biweekly: 52.5 (9.6); 30mg SC biweekly: 55.5 (9.9); 50mg SC biweekly: 51.1 (10.3)  50mg SC monthly: 54.1 (11.3); 100mg SC monthly: 54.4 (9.9); pbo: 54.0 (10.6) | albi 4mg SC QW: 42.9; 15mg SC QW: 51.4; 30mg SC QW: 25.8 15mg SC biweekly: 42.4; 30mg SC biweekly: 50.0; 50mg SC biweekly: 54.3 50mg SC monthly: 48.6; 100mg SC monthly: 55.9; pbo: 54.9 | USA, Mexico, Chile, Dominican Republic | not reported | albi 4mg SC QW: 4.4 (4.1); 15mg SC QW: 4.7 (4.6); 30mg SC QW: 5.2 (5.4) 15mg SC biweekly: 4.3 (4.3); 30mg SC biweekly: 5.5 (4.5); 50mg SC biweekly: 5.2 (5.5) 50mg SC monthly: 5.3 (3.7); 100mg SC monthly: 4.3 (3.7); pbo: 3.9 (3.0) |

**Characteristics of Included Studies – GLP1RA Added to Metformin Background continued**

| **Study ID** | **Baseline A1C % (SD)** | **Mean Change in HbA1C % vs. baseline** | **Mean Difference in HbA1c % vs pbo** | **HYPO def** | **Ascertainment of Hypo** | **Rescue Medication** | **Excl of Pts w events? At screening or during study)** |
| --- | --- | --- | --- | --- | --- | --- | --- |
| Ahrén B 2013 | lixi 20μg AM: 8.0 (0.9); 20μg PM: 8.1 (0.9); pbo: 8.1 (0.9) | [LSM] lixi 20μg AM: -0.9% (SE ±0.07); pbo: -0.4 (SE ±0.08); 20μg PM: -0.8 (SE ±0.07); pbo: -0.4 (SE ±0.08) | [LSM] lixi 20μg AM: -0.5 (SE ± 0.09) (95% CI -0.66 to -0.31); 20μg PM: -0.4 (SE ± 0.09) (95% CI -0.54 to -0.19) | Symptomatic hypoglycemia was defined as symptoms of hypoglycemia with an accompanying blood glucose <3.3 mmol/L (60 mg/dL) and/or prompt recovery with oral carbohydrate, intravenous glucose, or glucagon injection. Severe symptomatic hypoglycemia was defined as symptomatic hypoglycemia that required the assistance of another person, and that was associated either with a plasma glucose level <2.0 mmol/L (36 mg/dL) or, if no plasma glucose measurement was obtainable, with prompt recovery with carbohydrate, intravenous glucose, or glucagon injection. | not reported | Rescue confounding. Rescue (sulfonylureas as the first option) permitted. | One patient (0.4%)  in the lixisenatide morning injection arm and three patients (1.2%) in the lixisenatide  evening injection arm discontinued  study therapy owing to a  treatment-emergent AE of hypoglycemia  during the 24-week treatment period. |
| Bolli GB 2014 | lixi one-step: 8.0 (0.9); two-step: 8.1 (0.9); pbo: 8.0 (0.8) | [LSM] lixi one-step: -0.9 (SE ±0.10); two-step: -0.8 (SE ±0.1); pbo: -0.4 (SE ±0.10) | [LSM] lixi one-step: -0.5 (-0.7 to -0.3); two-step: -0.4 (-0.6 to -0.2) | Symptomatic hypoglycaemia defined as event with clinical symptoms with either plasma glucose < 60 mg/dl (3.3 mmol/L) or prompt recovery after oral carbohydrate administration (if no plasma glucose measurement was available), intravenous glucose or glucagon administration.  Severe hypoglycaemia—event with clinical symptoms considered to result from hypoglycaemia in which the participant required the assistance of another person, because the participant could not treat him/herself as a result of acute neurological impairment directly resulting from the hypoglycemic event, and one of the following: event was associated with plasma glucose < 2.0 mmol/l (36 mg/dl); if no plasma glucose measurement is available, then the event was associated with prompt recovery after oral carbohydrate, intravenous glucose or glucagon administration. | Patient-reported | Rescue [medications not specified] permitted. | no |
| Davies M 2017 | sema 2.5mg PO QD: 8.0 (0.7) 5mg PO QD: 7.8 (0.6) 10mg PO QD: 7.8 (0.7) 20mg PO QD: 7.9 (0.7) 40mg PO QD: 8.0 (0.7) 40mg PO QD slow dose (8w) escalation: 8.0 (0.7) 40mg PO QD fast dose (2w) escalation: 7.8 (0.8) pbo: 8.0 (0.8) | pooled sema 40mg: -1.8; pbo 0.3 (CI provided for ETD) | sema 2.5mg PO QD: -0.4 (-0.7, -0.1) 5mg PO QD: -0.9 (-1.2, -0.6) 10mg PO QD: -1.2 (-1.5, -0.9) 20mg PO QD: -1.4 (-1.7, -1.0) 40mg PO QD: -1.6 (-1.9, -1.3) 40mg PO QD slow dose (8w) escalation: -1.4 (-1.7, -1.1) 40mg PO QD fast dose (2w) escalation: -1.3 (-1.6, -1.0) | Severe or BG-confirmed’ hypoglycemic episodes are defined as severe by ADA classification or BG-confirmed with a plasma glucose value of <56 mg/dL (<3.1 mmol/L) with/without  symptoms consistent with hypoglycemia; ADA classified, ≤70 mg/dL (≤3.9 mmol/L); c ADA-classified ‘severe’ hypoglycemia is an episode requiring the assistance of another person to actively administer carbohydrate, glucagon, or take other corrective actions.  The typical signs and symptoms of confirmed hypoglycaemia include hunger, slight headache, nausea, light-headedness, palpitations and sweating. Major hypoglycaemia (severe) may produce loss of consciousness.  Symptoms of confirmed and not major hypoglycaemia should be treated by ingestion of carbohydrates. | Patient-reported; Plasma glucose should always be measured and recorded when a hypoglycemic episode is suspected. The record should include the following information:  Date and time of hypoglycemic episode, The plasma glucose level before treating the episode (if available), Whether the episode was symptomatic, Whether the subject was able to treat him/herself, Date and time of last trial product administration prior to episode, Type of last trial product prior to episode, Date and time of last main meal prior to episode, Whether the episode occurred in relation to increased physical activity The answer to the question: “Was subject able to treat him/herself?” must be answered “No” if oral carbohydrates, glucagon or IV glucose had to be administered to the subject by another person. | Rescue medication permitted. | no |
| DeFronzo RA 2005 | exen 5μg BID: 8.3 (1.1); 10μg BID: 8.2 (1.0); pbo: 8.2 (1.0) | [LSM] (SE) exen 5μg BID: -0.4 (0.1); 10μg BID: -0.8 (0.1); pbo: 0.1 (0.1) | [LSM] (manually calculated, no SE or CI) exen 5μg BID: -0.5; 10μg BID: -0.9 | Treatment-emergent adverse events were defined as those occurring upon or after receiving the first randomized dose. The intensity of hypoglycemic episodes was defined as mild/moderate or severe. For mild/moderate hypoglycemia, subjects reported symptoms consistent with hypoglycemia that may have been documented by a plasma glucose concentration value <3.3 mmol/l. For severe hypoglycemia, subjects required the assistance of another person to obtain treatment for their hypoglycemia, including intravenous glucose or intramuscular glucagon. | Patient-reported | not reported | no |
| Derosa G  2013 | exen 10μg BID: 8.1 (0.8); pbo: 7.9 (0.6) | exen 10μg BID: -1.2; pbo: -0.4 manually calculated | manually calculated  -0.8  (p<0.05) | Hypoglycemia defined as FPG <3.3mmol/L. | BG confirmed | not reported | no |
| Kim D 2007 | exen 0.8mg LAR: 8.6 (1.1); 2.0mg exen LAR: 8.3 (1.1); pbo: 8.6 (1.4) | exen 0.8mg LAR: -1.4 (SE ± 0.3); 2.0mg exen LAR: -1.7 (SE ± 0.3); pbo: 0.4 (SE ± 0.3) | exen 0.8mg LAR: -1.8; exen 2.0mg LAR: -2.1  manually calculated | not reported | Patient-reported  For self-monitored blood glucose profiles, subjects were given blood glucose  meters and instructed to perform  measurements by fingerstick at the fingertip.  Pre-prandial glucose was measured 15  min before each meal, postprandial glucose  was measured 1.5–2 h after each  meal, and an additional glucose measurement  was taken at 0300 h. Measurements  were recorded on 3 separate days for both  baseline and week 15. | not reported | No, but one patient withdrew due to loss of glycemic control. |
| Lingvay I  2018 | sema 0.05mg: 7.9 (0.7), 0.1mg: 7.9 (0.8), 0.2mg: 8.0 (0.8), 0.3mg: 8.2 (0.8); pbo: 8.1 (0.9) | sema 0.05mg: -1.1, 0.1mg: -1.4, 0.2mg: -1.7, 0.3mg: -1.9; pbo: -0.02 | sema 0.05mg: -1.04 (-1.30 to -0.77), 0.1mg: -1.34 (-1.61 to -1.08), 0.2mg: -1.69 (-1.95 to -1.42), 0.3mg: -1.86 (-2.12 to -1.60) | Only severe hypoglycemia defined as BG < 3.1 mmol/L  with symptoms consistent  with hypoglycemia | BG confirmed < 3.1 mmol/L | Patients requiring rescue therapy were discontinued. | no |
| Lingvay I  2018 | lira 0.3mg: 8.1 (0.9), 0.6mg: 8.1 (0.8), 1.2mg: 8.1 (0.9), 1.8mg: 8.1 (0.8); pbo: 8.1 (0.9) | lira 0.3mg: -0.5, 0.6mg: -0.9, 1.2mg: -0.8, 1.8mg: -1.3; pbo: -0.02 | manually calculated lira 0.3mg: -0.48, 0.6mg: -0.88, 1.2mg: -0.78, 1.8mg: -1.28 | Only severe hypoglycemia defined as BG < 3.1 mmol/L  with symptoms consistent  with hypoglycemia | BG confirmed < 3.1 mmol/L | Patients requiring rescue therapy were discontinued. | no |
| Nauck MA 2016 | sema QW 0.1mg: 8.2 (0.9); SC QW 0.2mg: 8.2 (0.9); SC QW 0.4mg: 8.1 (0.9); SC QW 0.8mg: 8.2 (0.9); SC 0.8mg E: 8.0 (0.8); SC QW 1.6mg E: 8.0 (0.7); pbo: 8.1 (0.8) | [LSM] sema QW 0.1mg: -0.6; SC QW 0.2mg: -0.9 (p<0.05); SC QW 0.4mg: -1.1 (p<0.001) ; SC QW 0.8mg:-1.5 (p<0.0001); SC 0.8mg E:-1.4 (p<0.0001); SC QW 1.6mg E:-1.7 (p<0.0001); pbo: -0.5 | [LSM] sema QW 0.1mg: -0.1 (-0.5 to 0.3); SC QW 0.2mg: -0.4 (-0.8 to -0.0); SC QW 0.4mg: -0.6 (-1.0 to -0.2); SC QW 0.8mg: -1.0 (-1.4 to -0.6); SC 0.8mg E: -1.0 (-1.3 to -0.6); SC QW 1.6mg E: -1.2 (-1.6 to -0.8) | Minor hypoglycemia defined as a plasma glucose level <3.1 mmol/L and therefore counted as any. Major hypoglycemia defined as requiring assistance and captured as severe. | patient-reported | not reported | no |
| Ratner RE 2010 | lixi 5μg SC QD: 7.58 (0.7); 10μg SC QD: 7.52 (0.6); 20μg SC QD: 7.58 (0.7); 30μg SC QD: 7.52 (0.7) 5μg SC BID: 7.60 (0.6); 10μg SC BID: 7.54 (0.6); 20μg SC BID: 7.61 (0.7); 30μg SC BID: 7.46 (0.5); pbo: 7.53 (0.6) | [LSM] lixi 5μg SC QD: -0.47 (p=0.0056); 10μg SC QD: -0.50 (p=0.0033); 20μg SC QD: -0.69 (p<0.0001)); 30μg SC QD: -0.76 (p<0.0001) 5μg SC BID: -0.65 (p<0.0001); 10μg SC BID: -0.78 (p<0.0001); 20μg SC BID: -0.75 p<0.0001; 30μg SC BID: -0.87 (p<0.001); pbo: -0.18 (n/a) | [LSM] manually calculated lixi 5μg SC QD: -0.29; 10μg SC QD: -0.32; 20μg SC QD: -0.51; 30μg SC QD: -0.58; 5μg SC BID: -0.47; 10μg SC BID: -0.60; 20μg SC BID: -0.57; 30μg SC BID: -0.69 | Symptomatic hypoglycaemia was defined as symptoms consistent with hypoglycaemia, with an accompanying blood glucose < 3.3 mmol⁄ l or prompt recovery with carbohydrate. Severe reported but not defined. | Patient-reported | not reported | no |
| Rosenstock J 2009 B | albi 4mg SC QW: 8.1 (1.0); 15mg SC QW: 8.0 (0.9); 30mg SC QW: 8.0 (0.9) 15mg SC biweekly: 8.2 (1.0); 30mg SC biweekly: 8.0 (1.0); 50mg SC biweekly: 8.0 (0.7) 50mg SC monthly: 7.9 (0.8); 100mg SC monthly: 8.0 (1.0); pbo: 7.9 (0.9) | albi 4mg SC QW: -0.11 (1.16); 15mg SC QW: -0.49 (0.74); 30mg SC QW: -0.87 (0.65)  15mg SC biweekly: -0.56 (0.97); 30mg SC biweekly: -0.79 (0.98); 50mg SC biweekly: -0.79 (1.04)  50mg SC monthly: -0.55 (1.01); 100mg SC monthly: -0.87 (0.87) | not reported. The primary efficacy end point was change from baseline A1C at week 16 versus placebo across different doses within each schedule (weekly, biweekly, and monthly). | not reported | not reported | not reported | no |

**Characteristics of Included Studies – SGLT2i Added to Metformin Background**

| **Study ID** | **Dose** | **Study Duration** | **n=** | **Mean Age (SD)** | **Gender (% male)** | **Countries Studied** | **Ethnicity (%)** | **Duration of Diabetes in Years (SD)** |
| --- | --- | --- | --- | --- | --- | --- | --- | --- |
| Amin NB 2015 | ertu 1mg QD; ertu 5mg QD; ertu 10mg QD; ertu 25mg QD | 12 weeks | ertu 1mg QD: 54; 5mg QD: 55; 10mg QD: 55; 25mg QD: 55; pbo: 54 | ertu 1mg QD: 53.1 (9.1); 5mg QD: 54.7 (7.7); 10mg QD: 57.3 (6.5); 25mg QD: 54.2 (8.8); pbo: 54 (8.1) | ertu 1mg QD: 63; 5mg QD: 74.5; 10mg QD: 56.4; 25mg QD: 67.3; pbo:56.6 | Canada, India, South Korea, Mexico, USA | not reported | ertu 1mg QD: 6.3 range (0.1 to 24); 5mg QD: 6.7 (0.3 to 30.0); 10mg QD: 6.1 (0.2 to 20.0); 25mg QD: 6.0 (0.3 to 18.2); pbo: 6.4 (0.3 to 20.5) |
| Bailey CJ 2010 | dapa 2.5mg QD; dapa 5mg QD; dapa 10mg QD | 24 weeks | dapa 2.5mg QD: 137; 5mg QD: 137; 10mg QD: 135; pbo: 137 | dapa 2.5mg QD: 55.0 (9.3); 5mg QD: 54.3 (9.4); 10mg QD: 52.7 (9.9); pbo: 53.7 (10.3) | dapa 2.5mg QD: 51; 5mg QD: 50; 10mg QD: 57; pbo: 55 | USA, Canada, Argentina, Mexico, Brazil | not reported | dapa 2.5mg QD: 6.0 (6.2); 5mg QD: 6.4 (5.8); 10mg QD: 6.1 (5.4); pbo: 5.8 (5.1) |
| Bolinder J 2012 | dapa 10mg QD | 24 weeks | dapa 10mg QD: 89; pbo: 91 | dapa 10mg: 60.6 (8.2); pbo: 60.8 (6.9) | dapa 10mg: 55.1; pbo: 56 | Bulgaria, Czech Republic, Hungary, Poland and Sweden | dapa 10mg white: 100; pbo: 100 | dapa 10mg: 6.0 (4.5); pbo: 5.5 (5.3) |
| Häring HU 2014 | empa 10mg QD; empa 25mg QD | 24 weeks | empa 10mg QD: 217; 25mg QD: 213; pbo: 207 | empa 10mg QD: 55.5 (9.9); 25mg QD: 55.6 (10.2); pbo: 56.0 (9.7) | empa 10mg QD: 58; 25mg QD: 56; pbo: 56 | Canada, China, France, Germany, India, South Korea, Mexico, Slovakia, Slovenia, Taiwan, Turkey, USA | empa 10mg QD: White 52, Asian 46, Black/African American 2, American Indian/Alaska Native 1; 25mg QD: White 53, Asian 46, Black/African American 0, American Indian/Alaska Native 1; pbo: White 55, Asian 44, Black/African American 1, American Indian/Alaska Native 0 | empa 10mg QD: ≤ 1 year: 9, >1-5 years: 36, >5-10 years 31, >10 years 24; 25mg QD: ≤ 1 year 9, >1-5 years 32, >5-10 years 35, >10 years 24; pbo: ≤ 1 year 9, >1-5 years 40, >5-10 years 31, >10 years 19 |
| Ikeda S 2015 | tofo 2.5mg QD; tofo 5mg QD; tofo 10mg QD; tofo 20mg QD; tofo 40mg QD | 12 weeks | tofo 2.5mg QD: 66; 5mg QD: 65; 10mg QD: 66; 20mg QD: 64; 40mg QD: 67; pbo: 66 | tofo 2.5mg QD: 53.3 (10.86); 5mg QD: 54.8 (10.53); 10mg QD: 54.5 (10.70); 20mg QD: 56.3 (9.79); 40mg QD: 57.5 (9.31); pbo: 53.9 (11.12) | tofo 2.5mg QD: 51.5; 5mg QD: 47.7; 10mg QD: 51.5; 20mg QD: 67.2; 40mg QD: 46.3; pbo: 54.5 | multinational; not reported | tofo [WHITE %] 2.5mg QD: 69.7; 5mg QD: 63.1; 10mg QD: 60.6; 20mg QD: 70.3; 40mg QD: 58.2; pbo: 63.6 | tofo 2.5mg QD: 4.88 (3.915); 5mg QD: 5.01 (3.599); 10mg QD: 5.77 (4.385); 20mg QD: 5.21 (3.934); 40mg QD: 6.44 (5.811); pbo: 5.98 (5.287) |
| Ji L  2019 | ertu 5mg QD; ertu 15mg QD | 26 weeks | ertu 5mg: 170; ertu 15mg: 169; pbo: 167 | ertu 5mg: 56.1 (9.0); ertu 15mg: 56.3 (9.3); pbo: 56.9 (9.0) | ertu 5mg: 55.9; ertu 15mg: 58.0; pbo: 52.7 | Asia | ertu 5mg Chinese 80.0, Hong Kong 5.9, Korean 7.6, Philippines 4.1, Taiwanese 2.4; ertu 15mg: Chinese 79.9, Hong Kong 5.9, Korean 5.9, Philippines 4.7, Taiwanese 3.6; pbo: Chinese 80.8, Hong Kong 4.2, Korean 5.4, Philippines 4.8, Taiwanese 4.8 | ertu 5mg: 7.0 (5.0); ertu 15mg: 7.5 (5.1); pbo: 6.4 (5.1) |
| Kashiwagi A 2015 | ipra 50mg QD | 24 weeks | ipra 50mg: 112; pbo: 56 | ipra 50mg: 56.2 (10.67); pbo: 57.7 (9.24) | ipra 50mg: 58.9; pbo: 58.9 | Japan | Japanese (112) | ipra 50mg: 7.49; pbo: 8.05 |
| Lu CH 2016 | ipra 50mg QD | 24 weeks | ipra 50mg: 87; pbo: 83 | ipra 50mg: 53.9 (11.3); pbo: 53.4 (11.3) | ipra 50mg: 50.6; pbo: 39.8 | South Korea, Taiwan | ipra 50mg: Korean (43), Taiwanese (44) | ipra 50mg: 6.49; pbo: 5.82 |
| Qiu R 2014 | cana 50mg BID; cana 150mg BID | 18 weeks | cana 50mg BID: 93; 150mg BID: 93; pbo: 93 | cana 50mg BID: 58.6 (8.9); 150mg BID: 56.7 (10.3); pbo: 57.0 (9.3) | cana 50mg BID: 43.0; 150mg BID: 47.3; pbo: 49.5 | 7 countries; not reported | cana 50mg BID: White 80.6, Black or African American 5.4, Asian 3.2, Other 10.8; 150mg BID: White 89.2, Black or African American 1.1, Asian 6.5, Other 3.2; pbo: White 78.5, Black or African American 4.3, Asian 9.7, Other 7.5 | cana 50mg BID: 6.7 (4.9); 150mg BID: 7.3 (6.0); pbo: 7.0 (6.4) |
| Rosenstock J 2012 | cana 50mg QD; cana 100mg QD; cana 200mg QD; cana 300mg QD; cana 300mg BID | 12 weeks | cana 50mg QD: 64; 100mg QD: 64; 200mg QD: 65; 300mg QD: 64; 300mg BID: 64; pbo: 65 | cana 50mg QD: 53.3 (8.5); 100mg QD: 51.7 (8.0); 200mg QD: 52.9 (9.6); 300mg QD: 52.3 (6.9); 300mg BID: 55.2 (7.1); pbo: 53.3 (7.8) | cana 50mg QD: 53; 100mg QD: 56; 200mg QD: 51; 300mg QD: 56; 300mg BID: 44; pbo: 48 | not reported | not reported | cana 50mg QD: 5.6 (5.0); 100mg QD: 6.1 (4.7); 200mg QD: 6.4 (5.7); 300mg QD: 5.9 (5.2); 300mg BID: 5.8 (4.6); pbo: 6.4 (5.0) |
| Rosenstock J 2013 | empa 1mg QD; empa 5mg QD; empa 10mg QD; empa 25mg QD; empa 50mg QD | 12 weeks | empa 1mg QD: 71; 5mg QD: 71; 10mg QD: 71; 25mg QD: 70; 50mg QD: 70; pbo: 71 | empa 1mg QD: 57 (8.8); 5mg QD: 60 (7.3); 10mg QD: 59 (9.0); 25mg QD: 59 (8.1); 50mg QD: 56 (9.4); pbo: 60 (8.5) | empa 1mg QD: 58; 5mg QD: 41; 10mg QD: 47; 25mg QD: 53; 50mg QD: 56; pbo: 47 | 16 countries; not specified | empa 1mg QD: Non-Hispanic White 86, Hispanic White 13, Non-Hispanic Black 0, Other 1; 5mg QD: Non-Hispanic White 83, Hispanic White 16, Non-Hispanic Black 0, Other 1; 10mg QD: Non-Hispanic White 78, Hispanic White 20, Non-Hispanic Black 3, Other 0; 25mg QD: Non-Hispanic White 83, Hispanic White 16, Non-Hispanic Black 1, Other 0; 50mg QD: Non-Hispanic White 86, Hispanic White 11, Non-Hispanic Black 3, Other 0; pbo: Non-Hispanic White 90, Hispanic White 9, Non-Hispanic Black 1, Other 0 | not reported |
| Rosenstock J 2015 | sota 75mg QD; sota 200mg QD; sota 200mg BID; sota 400mg QD | 12 weeks | sota 75mg QD: 59; 200mg QD: 60; 200mg BID: 60; 400mg QD: 60; pbo: 60 | sota 75mg QD: 56.1 (9.6); 200mg QD: 55.6 (9.3); 200mg BID: 56.4 (8.8); 400mg QD: 56.1 (9.5); pbo: 55.1 (9.8) | sota 75mg QD: 57.6; 200mg QD: 28.3; 200mg BID: 48.3; 400mg QD: 48.3; pbo: 43.3 | USA | sota 75mg QD: Black/African American 8.5, White 81.4, Other [American Indian/Alaska Native/Asian/Other/Multiple] 10.2; 200mg QD: Black/African American 15.0, White 85.0, Other [American Indian/Alaska Native/Asian/Other/Multiple] 0; 200mg BID: Black/African American 8.3, White 88.3, Other [American Indian/Alaska Native/Asian/Other/Multiple] 3.3; 400mg QD: Black/African American 10.0, White 85.0, Other [American Indian/Alaska Native/Asian/Other/Multiple] 5.0; pbo: Black/African American 10.0, White 81.7, Other [American Indian/Alaska Native/Asian/Other/Multiple] 8.3 | not reported |
| Rosenstock J  2018 | ertu 5mg QD; ertu 15mg QD | 26 weeks | ertu 5mg: 207; ertu 15mg: 205; pbo: 209 | ertu 5mg: 56.6 (8.1); ertu 15mg: 56.9 (9.4); pbo: 56.5 (8.7) | ertu 5mg: 46.9; ertu 15mg: 45.4; pbo: 46.9 | North America, South America, Europe, Asia, South Africa, Australia, New Zealand | ertu 5mg Asian: 16.4, Black or African American: 10.6, Multiple: 8.2, White: 64.7; ertu 15mg Asian: 17.1, Black or African American: 11.2, Multiple: 6.8, White: 64.9; pbo Asian: 14.8, Black or African American: 9.1, Multiple: 7.2, White: 68.9 | ertu 5mg: 7.9 (6.1); ertu 15mg: 8.1 (5.5); pbo: 8.0 (6.3) |
| Ross S 2015 | empa 12.5mg BID; empa 25mg QD; empa 5mg BID; empa 10mg QD | 16 weeks | empa 12.5mg BID: 215; 25mg QD: 214; 5mg BID: 215; 10mg QD: 214; pbo: 107 | empa 12.5mg BID: 57.6 (9.9); 25mg QD: 58.2 (10.2); 5mg BID: 58.8 (9.8); 10mg QD: 58.5 (10.8); pbo: 57.9 (11.2) | empa 12.5mg BID: 57.2; 25mg QD: 53.3; 5mg BID: 55.8; 10mg QD: 50.5; pbo: 51.4 | Countries not specified; Europe, North American, Latin America | empa 12.5mg BID: White 81.9, Black/African-American 7.9, Asian 7.0, Other 3.3; 25mg QD: White 89.3, Black/African-American 4.7, Asian 4.2, Other 1.9; 5mg BID: White 87.9, Black/African-American 7.9, Asian 2.8, Other 1.4; 10mg QD: White 84.1, Black/African-American 6.5, Asian 4.7, Other 4.7; pbo: White 86.9, Black/African-American 7.5, Asian 1.9, Other 3.7 | (time since dx.) empa 12.5mg BID: ≤ 1 year 12.6; >1 to ≤5 years 35.8; >5 years 51.6; 25mg QD: ≤ 1 year 7.0; >1 to ≤5 years 39.3; >5 years 53.7; 5mg BID: ≤ 1 year 9.3; >1 to ≤5 years 35.8; >5 years 54.9; 10mg QD: ≤ 1 year 6.5; >1 to ≤5 years 31.8; >5 years 61.7; pbo: ≤ 1 year 8.4; >1 to ≤5 years 29.0; >5 years 62.6 |
| Schumm-Draeger PM 2015 | dapa 2.5mg BID; dapa 5mg BID; dapa 10mg QD | 16 weeks | dapa 2.5mg BID: 100; 5mg BID: 99; 10mg QD: 99; pbo: 101 | dapa 2.5mg BID: 58.3 (9.0); 5mg BID: 55.3 (9.3); 10mg QD: 58.5 (9.8); pbo: 58.5 (9.4) | dapa 2.5mg BID: 37.0; 5mg BID: 46.5; 10mg QD: 49.5; pbo: 46.5 | Countries not specified; Europe, South Africa | dapa 2.5mg BID: White 79.0, Black 10.0, Asian 8.0, Other 3.0; 5mg BID: White 84.8, Black 5.1, Asian 7.1, Other 3.0; 10mg QD: White 81.8, Black 6.1, Asian 3.0, Other 9.1; pbo: White 81.2, Black 5.0, Asian 9.9, Other 4.0 | dapa 2.5mg BID: 4.80 (3.87); 5mg BID: 5.12 (4.2); 10mg QD: 5.45 (4.05); pbo: 5.53 (4.23) |
| Shestakova MV  2018 | ipra 50mg QD | 24 weeks | ipra 50mg: 110; pbo: 55 | ipra 50mg: 58.9 (9.3); pbo: 58.0 (9.5) | ipra 50mg: 43.6; pbo: 40.0 | Russia | ipra 50mg White: 100, Asian: 0; pbo White: 98.2, Asian: 1.8 | ipra 50mg: 6.65; pbo: 6.56 |
| Wilding JPH 2013 | ipra 12.5mg QD; ipra 50mg QD; ipra 150mg QD; ipra 300mg QD | 12 weeks | ipra 12.5mg QD: 69; 50mg QD: 68; 150mg QD: 67; 300mg QD: 72; pbo: 66 | ipra 12.5mg QD: 56.6 (8.5); 50mg QD: 58.6 (7.6); 150mg QD: 58.1 (8.2); 300mg QD: 56.6 (8.9); pbo: 57.3 (8.6) | ipra 12.5mg QD: 47.8; 50mg QD: 47.1; 150mg QD: 56.7; 300mg QD: 50.0; pbo: 54.5 | not reported | ipra 12.5mg QD: White 91.3, Non-white 8.7; 50mg QD: White 97.1, non-white 2.9; 150mg QD: white 97.0, non-white 3.0; 300mg QD: white 90.3, non-white 9.7; pbo: white 95.5, non-white 4.5 | ipra 12.5mg QD: 6.8 (6.4); 50mg QD: 6.0 (5.3); 150mg QD: 5.7 (4.8); 300mg QD: 5.5 (4.8); pbo 5.7 (3.2) |
| Yang W 2016 | dapa 5mg QD; dapa 10mg QD | 24 weeks | dapa 5mg QD: 147; 10mg QD: 152; pbo: 145 | dapa 5mg QD: 53.1 (9.1); 10mg QD: 54.6 (9.5); pbo: 53.5 (9.2) | dapa 5mg QD: 45.6; 10mg QD: 57.9; pbo: 59.3 | China, India, South Korea | dapa 5mg QD: Asian Indian 7.5, Chinese 86.4, Korean 6.1; 10mg QD: Asian Indian 8.6, Chinese 84.9, Korean 6.6; pbo: Asian Indian 6.9, Chinese 86.9, Korean 6.2 | dapa 5mg QD: 4.2 (3.8); 10mg QD: 5.3 (4.6); pbo 5.3 (4.4) |

**Characteristics of Included Studies – SGLT2i Added to Metformin Background continued**

| **Study ID** | **Baseline A1C % (SD)** | **Mean Change in HbA1C % vs. baseline** | **Mean Difference in HbA1c % vs pbo** | **HYPO def** | **Ascertainment of Hypo** | **Rescue Medication** | **Excl of Pts w events? At screening or during study)** |
| --- | --- | --- | --- | --- | --- | --- | --- |
| Amin NB 2015 | ertu 1mg QD: 8.01 (0.17); 5mg QD: 7.88 (0.13); 10mg QD: 8.13 (0.17); 25mg QD: 8.30 (0.16); pbo: 8.08 (0.14) | [LSM] (80% CI) ertu 1mg QD: -0.56 (-0.69 to -0.42); 5mg QD: -0.80 (-0.84 to -0.66); 10mg QD: -0.73 (-0.87 to -0.58); 25mg QD: -0.83 (-0.98 to -0.69); pbo: -0.11 (-0.25 to 0.04) | [LSM] manually calculated ertu 1mg QD: -0.45; 5mg QD: -0.69; 10mg QD: -0.62; 25mg QD: -0.72 | Hypoglycaemia was captured as an AE based on signs/symptoms, home blood glucose monitoring [defined as blood glucose ≤3.9mmol/l (70mg/dl) using an ACCU-CHEK home glucometer, or ≤4.1mmol/l (74mg/dl) using International Federation of Clinical Chemistry-referenced ACCU-CHEK glucometers]. Severe not defined. | Home blood glucose monitoring using an ACCU-CHEK  home glucometer, or ≤4.1mmol/l (74mg/dl) using International  Federation of Clinical Chemistry-referenced  ACCU-CHEK glucometers; The recommended home glucose monitoring frequency  was determined for each subject by the investigator; however,  a frequency of at least once daily (and in event of hypoglycaemia)  was emphasized. | Rescue not permitted. | no |
| Bailey CJ 2010 | dapa 2.5mg QD: 7.99 (0.90); 5mg QD: 8.17 (0.96); 10mg QD: 7.92 (0.82); pbo: 8.11 (0.96) | dapa 2.5mg QD: -0.67 (-0.81 to -0.53); 5mg QD: -0.70 (-0.85 to -0.56); 10mg QD: -0.84 (-0.98 to -0.70); pbo: -0.30 (-0.44 to -0.16) | manually calculated dapa 2.5mg QD: -0.37; 5mg QD: -0.40; 10mg QD: -0.54 | Major event, defined as a symptomatic episode requiring third party assistance because of severe impairment in consciousness or behavior, with a capillary or plasma glucose concentration <3 mmol/L, and prompt recovery after glucose or glucagon administration. | not reported | Rescue confounding. Rescue (pioglitazone or acarbose) permitted. For rescued patients, measurements obtained after initiation of rescue medication were not included in the efficacy analysis but were included in the safety analysis. | no |
| Bolinder J 2012 | dapa 10mg: 7.19 (0.44); pbo: 7.16 (0.53) | dapa 10mg: -0.39; pbo: -0.10 (n/a) | dapa 10mg: -0.28 (-0.42 to -0.15) | Major hypoglycemia was defined as a symptomatic episode requiring external assistance due to severely impaired consciousness or behavior, with capillary or plasma glucose levels below 3.0 mmol/liter and recovery after glucose or glucagon administration. Minor hypoglycemia was defined as either symptomatic episode with capillary or plasma glucose levels below 3.5 mmol/liter, irrespective of the need for external assistance, or an asymptomatic episode with capillary or plasma glucose levels below 3.5 mmol/liter that does not qualify as a major episode. Other hypoglycemia was defined as an episode with symptoms suggestive of hypoglycemia but without confirmative measurement. | not reported | Rescue confounding. Patients could receive rescue therapy exclusively with sitagliptin. Safety, not efficacy analysis includes patients after rescue therapy. | Patients could be discontinued due to inadequate glycemic control at the discretion of the study investigator. |
| Häring HU 2014 | empa 10mg QD: 7.94 (0.79); 25mg QD: 7.86 (0.87); pbo: 7.9 (0.88) | empa 10mg QD: -0.70 (0.05); 25mg QD: -0.77 (0.05); pbo: -0.13 (0.05) | empa 10mg QD: -0.57 (-0.70 to -0.43); 25mg QD: -0.64 (-0.77 to -0.50) differences in adjusted mean values versus placebo | Events consistent with hypoglycemia and with plasma glucose levels of ≤3.9 mmol/L and/or requiring assistance. Severe not defined. | Patient-reported. | Rescue permitted. Data after rescue set to missing and LOCF for efficacy values. | Yes. Where hyperglycemia or hypoglycemia  could not be controlled, the patient  was discontinued from the trial. |
| Ikeda S 2015 | tofo 2.5mg QD: 7.99 (0.759); 5mg QD: 8.01 (0.657); 10mg QD: 8.00 (0.713); 20mg QD: 7.92 (0.790); 40mg QD: 7.92 (0.780); pbo: 7.88 (0.694) | [LSM] tofo 2.5mg QD: -0.440 (-0.588 to -0.293); 5mg QD: -0.617 (-0.763 to -0.471); 10mg QD: -0.694 (-0.840 to -0.549); 20mg QD: -0.768 (-0.916 to -0.620); 40mg QD: -0.832 (-0.976 to -0.688); pbo: -0.269 (-0.415 to -0.123) LOCF | [LSM] manually calculated tofo 2.5mg QD: -0.171; 5mg QD: -0.348; 10mg QD: -0.425; 20mg QD: -0.499; 40mg QD: -0.563 | Plasma glucose ≤2.8mmol/l. Severe not separately defined. | not reported | not reported | no |
| Ji L  2019 | ertu 5mg: 8.1 (0.9); ertu 15mg: 8.1 (0.9); pbo: 8.1 (1.0) | [LSM] ertu 5mg: -1.0 (-1.1 to -0.9); ertu 15mg: -0.9 (-1.0 to -0.8); pbo: -0.2 (-0.3 to -0.1) | [LSM] ertu 5mg: -0.8 (-1.0 to -0.6); ertu 15mg: -0.7 (-0.9 to -0.5) | Hypoglycemia defined as symptomatic, documented (episodes with a glucose value of ≤3.9 mmol/l with or without symptoms) or severe. | not reported | Rescue therapy (glimepiride) permitted. Hypoglycemia  after rescue administration not included. | no |
| Kashiwagi A 2015 | ipra 50mg: 8.25 (0.719); pbo: 8.38 (0.738) | ipra 50mg: -0.87 (0.655); pbo: 0.38 (0.703) | ipra 50mg: -1.30 (-1.501 to -1.095) Adj mean difference | not reported | Patients were instructed how to recognize and treat the symptoms of hypoglycemia. | not reported | no |
| Lu CH 2016 | ipra 50mg: 7.74 (0.78); pbo: 7.75 (0.71) NGSP (National Glycohemoglobin Standardization Program) | ipra 50mg: -0.94 (0.747); pbo: -0.47 (0.806) | ipra 50mg: -0.46 (-0.66 to -0.27) | not reported | not reported | not reported | no |
| Qiu R 2014 | cana 50mg BID: 7.6 (0.9); 150mg BID: 7.6 (0.9); pbo: 7.7 (0.9) | [LSM] cana 50mg BID: -0.45; 150mg BID: -0.61; pbo: -0.01 | [LSM] cana 50mg BID: -0.44; 150mg BID: -0.60 (p<0.001 for both) | Documented hypoglycemia episodes included biochemically documented episodes (concurrent fingerstick or plasma glucose ≤3.9 mmol/L with or without symptoms) and severe episodes (i.e., those requiring the assistance of another individual or resulting in seizure or loss of consciousness. | biochemically documented only | not reported | no |
| Rosenstock J 2012 | cana 50mg QD: 8.00 (0.99); 100mg QD: 7.83 (0.96); 200mg QD: 7.61 (0.80); 300mg QD: 7.69 (1.02); 300mg BID: 7.73 (0.89); pbo: 7.75 (0.83) | [LSM] cana 50mg QD: -0.79; 100mg QD: -0.76; 200mg QD: -0.70; 300mg QD: -0.92; 300mg BID: -0.95; pbo: -0.22 | [LSM] manually calculated cana 50mg QD: -0.57; 100mg QD: -0.54; 200mg QD: -0.48; 300mg QD: -0.70; 300mg BID: -0.73 | not reported | patient-reported | not reported | no |
| Rosenstock J 2013 | empa 1mg QD: 7.8 (0.7); 5mg QD: 8.0 (0.7); 10mg QD: 7.9 (0.7); 25mg QD: 8.1 (0.8); 50mg QD: 7.9 (0.7); pbo: 8.0 (0.7) | empa 1mg QD: -0.09 (-0.24 to 0.07); 5mg QD: -0.23 (-0.39 to -0.08); 10mg QD: -0.56 (-0.71 to -0.41); 25mg QD: -0.55 (-0.70 to -0.40); 50mg QD: -0.49 (-0.64 to -0.33); pbo: 0.15 (-0.00 to 0.30) | manually calculated empa 1mg QD: -0.24; 5mg QD: -0.38; 10mg QD: -0.71; 25mg QD: -0.70; 50mg QD: -0.64 | Defined by preferred MedDRA terms.; severe plasma glucose levels  <3.0 mmol/l. | Events were assessed at screening and  visits 2−7 | not reported | no |
| Rosenstock J 2015 | sota 75mg QD: 8.0 (0.9); 200mg QD: 8.3 (1.0); 200mg BID: 8.4 (0.9); 400mg QD: 8.1 (1.0); pbo: 7.9 (0.9) | [LSM] sota 400 mg: -0.92 (p<0.001); pbo: -0.09 (p=0.403) | [LSM] manually calculated sota 75mg QD: -0.33; 200mg QD: -0.43; 200mg BID: -0.71; 400mg QD: -0.83 | not reported | not reported | Rescue permitted. Efficacy data collected after initiation of rescue therapy were excluded from analysis and  subjected to the LOCF algorithm as needed. | Yes, exclusion criteria: Has had 2 or more emergency room visits, doctors’ visits, or hospitalizations due to hypoglycemia within the 6 months -> but not excluded for hypoglycemic events occurring in current study. |
| Rosenstock J  2018 | ertu 5mg: 8.1 (0.9); ertu 15mg: 8.1 (0.9); pbo: 8.2 (0.9) | ertu 5mg: -0.7 (0.9); ertu 15mg: -1.0 (0.9); pbo: -0.2 (0.9) | ertu 5mg: -0.7 (-0.9 to -0.5); ertu 15mg: -0.9 (-1.0 to -0.7) | Documented hypoglycemia, defined as episodes with a  glucose level ≤3.9 mmol/L, with or without symptoms. Symptomatic hypoglycemia was also captured. Severe hypoglycemia was defined as an episode that required assistance, either medical or non-medical. | not reported | Rescue permitted, values after receipt of rescue. | no |
| Ross S 2015 | empa 12.5mg BID: 7.78 (0.79); 25mg QD: 7.73 (0.79); 5mg BID: 7.79 (0.88); 10mg QD: 7.84 (0.75); pbo: 7.69 (0.72) | empa 12.5mg BID: -0.84 (0.05); 25mg QD: -0.72 (0.05); 5mg BID: -0.68 (0.05); 10mg QD: -0.66 (0.05); pbo: -0.21 (0.08) | empa 12.5mg BID: -0.63 (0.10); 25mg QD: -0.52 (0.10); 5mg BID: -0.47 (0.10); 10mg QD: -0.45 (0.10) (all p<0.001) | Confirmed hypoglycemic AEs were defined as AEs  with plasma glucose ≤3.9mmol/l and/or requiring assistance. | not reported | Rescue medication permitted. Values observed after a patient started rescue therapy were set to missing and imputed  using a LOCF. | no |
| Schumm-Draeger PM 2015 | dapa 2.5mg BID: 7.77 (0.75); 5mg BID: 7.78mg (0.76); QD10mg QD: 7.71 (0.71); pbo: 7.94 (0.85) | dapa 2.5mg BID: -0.52 (0.0594); 5mg BID: -0.65 (0.0600); 10mg QD: -0.59 (0.0598); pbo: -0.30 (0.0593) Difference in adjusted mean change from baseline (SE) | Difference in adjusted mean change from baseline versus placebo+MET-IR (SE) (CI). dapa 2.5mg BID: -0.22 (0.0840) [-0.38 to -0.05]; 5mg BID: -0.35 (0.0843) [-0.52 to -0.18]; 10mg QD: -0.29 (0.0844) [-0.45 to -0.12] | Hypoglycemia was defined as a low blood glucose reading [<3.5mmol/l (<63 mg/dl)], with or without symptoms; a symptomatic event without a blood glucose reading was considered ‘suggestive of hypoglycemia’. Major defined as a symptomatic event requiring third-party assistance with a capillary or plasma glucose value (<3.0mmol/l (<54 mg/dl)). | not reported | Rescue not allowed and patients were discontinued from study. An increase in metformin dosing. | not reported |
| Shestakova MV  2018 | ipra 50mg: 8.39 (0.93); pbo: 8.46 (0.96) | ipra 50mg: -1.01 (0.85); pbo: -0.77 (1.10) | -0.26 (-0.53 to 0.00; p=0.0048) | Classification of hypoglycemic events follow the definitions from the ADA and Endocrine Society workgroup  report | not reported | not reported | no |
| Wilding JPH 2013 | ipra 12.5mg QD: 7.78 (0.64); 50mg QD: 7.76 (0.66); 150mg QD: 7.73 (0.69); 300mg QD: 7.87 (0.82); pbo 7.68 (0.60) | [LSM] (95% CI of LS mean) ipra 12.5mg QD: -0.53 (-0.71 to -0.36); 50mg QD: -0.65 (-0.83 to -0.47); 150mg QD: -0.72 (-0.90 to -0.53); 300mg QD: -0.79 (-0.97 to -0.62); pbo -0.31 (-0.50 to -0.13) | [LSM] manually calculated ipra 12.5mg QD: -0.22; 50mg QD: -0.34; 150mg QD: -0.41; 300mg QD: -0.48 | not reported | Patients were provided with a glucometer (Accu-Chek Performa; Hoffmann-La Roche Ltd, Basel, Switzerland) and asked to monitor capillary blood glucose twice daily (fasted and 2 h after a meal) and also when symptoms of hypoglycemia occurred. As classified by the investigator (not reported). | not reported | no |
| Yang W 2016 | dapa 5mg QD: 8.09 (0.72); 10mg QD: 8.17 (0.84); pbo 8.13 (0.85) | dapa 5mg QD: -0.82 (-0.94 to -0.70); 10mg QD: -0.85 (-0.96 to -0.73); pbo -0.23 (-0.35 to -0.11) | dapa 5mg QD: -0.59 (-0.76 to -0.42); 10mg QD: -0.62 (-0.79 to -0.45) | Reported hypoglycemic episodes were classified as major, minor, or ‘other’ according to symptoms, capillary or plasma glucose levels, and the requirement for external assistance. A major event was defined as a symptomatic episode requiring external (third party) assistance due to severe impairment in consciousness or  behavior with a capillary or plasma glucose value <3 mmol/L (<54 mg/dL) and prompt recovery after glucose or glucagon administration. | Patient-reported; not biochemically confirmed | Rescue confounding. Rescue (pioglitazone) permitted. Safety was summarized descriptively and included data after  rescue. | no |

**Characteristics of Included Studies – Second AHA Added to non-metformin Background**

| **Study ID** | **Dose** | **Study Duration** | **n=** | **Mean Age (SD)** | **Gender (% male)** | **Countries Studied** | **Ethnicity (%)** | **Duration of Diabetes in Years (SD)** |
| --- | --- | --- | --- | --- | --- | --- | --- | --- |
| Kadowaki T 2017 | cana 100mg QD  (as add-on to teneli) | 24 weeks | cana 100mg: 70; pbo: 68 | cana 100mg: 58.4 (8.9); pbo: 56.0 (9.5) | cana 100mg: 77.1; pbo: 77.9 | Japan | Japanese | cana 100mg: 8.34 (7.74); pbo: 6.50 (3.89) |
| Kadowaki T 2018 | teneli 20mg QD  (as add-on to cana) | 24 weeks | teneli 20mg: 77; pbo: 77 | teneli 20mg: 55.9 (8.3); pbo: 54.1 (10.2) | teneli 20mg: 83.1; pbo: 75.3 | Japan | Japanese | teneli 20mg: 8.15 (5.86); pbo: 7.34 (5.34) |
| Kaku K  2018 | lina 5mg QD  (as add-on to empa using  FDC of empa 10mg/lina 5mg (Part A) | 24 weeks (Part A) | Part A  empa 10mg/lina 5mg: 107; empa10mg/pbo: 108 | Part A  empa 10mg/lina 5mg: 58.0 (9.3); empa10mg/pbo: 56.3 (9.9) | Part A  empa 10mg/lina 5mg: 79.4; empa10mg/pbo: 78.7 | Japan | Japanese | Part A  empa 10mg/lina 5mg: 8.4 (5.6); empa10mg/pbo: 7.6 (5.0) |
| Kawamori R  2018 | empa 10mg QD  (as add on to lina 5mg in FDC empa/lina 10/5 mg QD | 24 weeks | empa 10mg QD: 182; pbo: 93 | empa 10mg QD: 60.0 (9.9); pbo: 59.8 (10.8) | empa 10mg QD: 78; pbo: 77.4 | Japan | Japanese | empa 10mg QD: 9.0 (7.2); pbo: 8.7 (6.1) |
| Odawara M 2015 | FDC of vilda 50mg/met 250mg bid or vilda 50mg/ met 500 mg bid  (patients previously on vilda monotherapy) | 14 weeks | vilda met: 115; vilda pbo: 56 | vilda met: 57.5 (10.9); vilda pbo: 56.2 (9.8) | vilda met: 71.3; vilda pbo: 71.4 | Japan | Japanese | vilda met: 7.0 (6.5); vilda pbo: 7.1 (6.9) |

**Characteristics of Included Studies – Second AHA Added to non-metformin Background continued**

| **Study ID** | **Baseline A1C % (SD)** | **Mean Change in HbA1C % vs. baseline** | **Mean Difference in HbA1c % vs pbo** | **HYPO def** | **Ascertainment of Hypo** | **Rescue Medication** | **Excl of Pts w events? At screening or during study)** |
| --- | --- | --- | --- | --- | --- | --- | --- |
| Kadowaki T 2017 | cana 100mg: 8.18 (0.90); pbo: 7.87 (0.83) | [LSM] (SE) cana 100mg: -0.97 (0.10); pbo: -0.10 (0.10) | [LSM] (SE) cana 100mg: -0.88 (-1.15 to -0.60) | Severe hypoglycemia: An event requiring assistance of another person to administer carbohydrate, glucagon, or other resuscitative actions Symptomatic hypoglycemia: Typical hypoglycemia symptoms with blood glucose levels of ≤70 mg/dL (3.89 mmol/L) at the time of onset Asymptomatic hypoglycemia: No typical hypoglycemia symptoms, but blood glucose levels were ≤70 mg/dL (3.89 mmol/L) Suspected symptomatic hypoglycemia: Typical hypoglycemia symptoms, but blood glucose levels were not measured. Relative hypoglycemia: Typical hypoglycemia symptoms with blood glucose levels >70 mg/dL at the time of onset. | Subjects were instructed to fill out the required information in their patient diaries. If patients observed symptoms of hypoglycemia, they performed self-monitoring of blood glucose (SMBG) when possible and visited their doctor immediately if the administration of sucrose (sugar) did not alleviate the symptoms. Subjects recorded symptoms of hypoglycemia and data of SMBG (if possible) in their patient diary. Investigators instructed every subject to bring their patient diaries to each study center visit for viewing. If the investigator considered any events in the diary to be hypoglycemia that occurred after initiation of the blinded study drugs, they noted this in the hypoglycemia field in the clinical report form. Any such events that were considered adverse events but not hypoglycemia was noted in the adverse event field of the clinical report form. | not reported | no |
| Kadowaki T 2018 | teneli 20mg: 7.98 (0.80); pbo: 8.09 (0.85) | [LSM] (SE) teneli 20mg: -0.94 (0.08); pbo: 0.00 (0.08) | [LSM] (SE) teneli 20mg: -0.94 (-1.16 to -0.72) | not reported | not reported | not reported | no |
| Kaku K  2018 | Part A  empa 10mg/lina 5mg: 8.34 (0.54); empa10mg/pbo: 8.40 (0.68) | Adjusted mean (SE)  Part A  empa 10mg/lina 5mg: -0.94 (0.05); empa10mg/pbo: -0.12 (0.06) | Part A: -0.82  (-0.97 to -0.67) | Hypoglycemia defined as symptomatic or confirmed (plasma glucose levels ≤ 3.9 mmol/L or requirement of assistance). | not reported | Rescue permitted with exception of SGLT2i, DPP4i and GLP1RA | no |
| Kawamori R  2018 | empa 10mg QD: 8.27 (0.65); pbo: 8.36 (0.74) | empa 10mg QD: -0.93 (SE 0.06); pbo: 0.21 (0.09) | empa 10mg: -1.14 (-1.36 to -0.91) (p<0.0001) | Hypoglycemia defined as plasma glucose levels ≤3.9 mmol/L or  requiring assistance. | not reported | Rescue permitted except with GLP1RA, DPP4i or SGLT2i | no |
| Odawara M 2015 | ≤ 8% vilda met: 67%; vilda pbo: 66.1%; >8 to ≤ 9% vilda met: 20.0%; vilda pbo: 19.6%; >9% vilda met: 13.0%; vilda pbo: 14.3% | vilda met 50/250mg bid: -0.6 (p<0.001); vilda met50/500mg bid: -1.0 (p<0.001); vilda pbo: 0.1 (FAS) | vilda met 50/250mg bid: -0.7; vilda met 50/500mg bid: -1.1  (manually calculated) | Hypoglycemia was defined as symptoms suggestive of hypoglycemia, further confirmed by self-monitored blood glucose measurement of <3.1 mmol/L. The event was considered severe if the patient required assistance of another person or hospitalization. | Patients were asked to record  hypoglycemic events in a study diary. | Rescue not permitted | no |

**Characteristics of Included Studies – Dual Therapy Initiation**

| **Study ID** | **Dose** | **Study Duration** | **n=** | **Mean Age (SD)** | **Gender (% male)** | **Countries Studied** | **Ethnicity (%)** | **Duration of Diabetes in Years (SD)** |
| --- | --- | --- | --- | --- | --- | --- | --- | --- |
| Goldstein BJ 2007 | sita 50mg + met 500mg BID; sita 50mg + met 1000mg BID | 24 weeks | sitagliptin 50mg + metformin 500mg BID: 190; sitagliptin 50mg + metformin 1000mg BID: 182; placebo: 176 | sitagliptin 50mg + metformin 500mg BID: 54.1 (10.0); sitagliptin 50mg + metformin 1000mg BID: 53.3 (9.6); placebo 53.6 (10.0) | sitagliptin 50mg + metformin 500mg BID: 55.3; sitagliptin 50mg + metformin 1000mg BID: 42.3; pbo: 52.8 | multinational; not specified | sitagliptin 50mg + metformin 500mg BID: White 53.7, Black 6.8, Hispanic 28.9, Asian 4.7, Other 5.8; sitagliptin 50mg + metformin 1000mg BID: White 52.2, Black 7.7, Hispanic 26.9, Asian 6.0, Other 7.1; pbo: White 46.0, Black 9.7, Hispanic 26.7, Asian 6.8, Other 10.8 | sitagliptin 50mg + metformin 500mg BID: 4.5 (4.7); sitagliptin 50mg + metformin 1000mg BID: 4.4 (4.2); pbo: 4.6 (4.9) |
| Haak T 2012 | lina 2.5mg BID + 500mg met BID; lina 2.5mg BID + 1000mg met BID | 24 weeks | lina 2.5mg BID + 500mg met BID: 143; lina 2.5mg BID + 1000mg met BID: 143; pbo: 72 | lina 2.5mg BID + 500mg met BID: 55.6 (11.2); lina 2.5mg BID + 1000mg met BID: 56.4 (10.7); pbo: 55.7 (11.0) | lina 2.5mg BID + 500mg met BID: 51.0; lina 2.5mg BID + 1000mg met BID: 53.8; pbo: 50.0 | 14 countries; not specified | lina 2.5mg BID + 500mg met BID: white 72.0, Asian 25.9, Black 1.4, Hawaiian Pacific Islander 0.7; lina 2.5mg BID + 1000mg met BID: white 65.7, Asian 33.6, Black 0.7, Hawaiian/Pacific Islander 0.0; pbo: White 63.9, Asian 36.1, Black 0.0, Hawaiian/Pacific Islander 0.0 | lina 2.5mg BID + 500mg met BID: ≤1: 38.0, > 1 to 5: 33.6, > 5: 28.5; lina 2.5mg BID + 1000mg met BID: ≤1: 36.4, > 1 to 5: 35.7, > 5: 27.9; pbo: ≤1: 30.8, > 1 to 5: 35.4, > 5: 33.8 |
| Ji L 2016 | sita 50mg BID + met 500mg BID; sita 50mg BID + met 850mg BID | 24 weeks | sita 50mg BID + met 500mg BID: 122; sita 50mg BID + met 850mg BID: 125; pbo: 126 | sita 50mg BID + met 500mg BID: 52.6 (11.3); sita 50mg BID + met 850mg BID: 52.4 (9.3); pbo 53.6 (9.7) | sita 50mg BID + met 500mg BID: 69.7; sita 50mg BID + met 850mg BID: 53.6; pbo: 68.5 | China | sita 50mg BID + met 500mg BID: Asian 100; sita 50mg BID + met 850mg BID: Asian 100; pbo 100 | sita 50mg BID + met 500mg BID: 1.1 (0.3); sita 50mg BID + met 850mg BID: 1.1 (0.3); pbo: 1.1 (0.2) |
| Ji L 2017 | alo 12.5mg + met 500mg FDC BID | 26 weeks | alo + met: 158; pbo: 161 | alo+met: 53.4 (10.46); pbo: 52.2 (10.17) | alo+met: 57.2; pbo: 58.3 | China | alo+met: Asian 100; pbo: Asian 98.8, American Indian or Alaskan Native 1.2, Multiracial 0 | not reported |
| Miller S 2018 | ertu 5mg QD + sita 100mg QD; ertu 15mg QD + sita 100mg QD | 26 weeks | ertu 5mg QD + sita 100mg QD: 98; ertu 15mg QD + sita 100mg QD: 96; pbo: 97 | ertu 5mg QD + sita 100mg QD: 56.4 (9.3); ertu 15mg QD + sita 100mg QD: 56.1 (10.1); pbo: 54.3 (10.3) | ertu 5mg QD + sita 100mg QD: 58.2; ertu 15mg QD + sita 100mg QD: 55.2; pbo: 58.8 | USA | ertu 5mg QD + sita 100mg QD: White 93.9, Black or African American 2.0, American Indian or Alaska Native 4.1, Multiple 0.0, Native Hawaiian or other Pacific Islander (0), Hispanic or Latino 34.7; ertu 15mg QD + Sita 100mg QD: White 84.4, Black or African American 7.3, American Indian or Alaska Native 6.3, Multiple 1.0, Native Hawaiian or other Pacific Islander 1.0, Hispanic or Latino 35.4; pbo: White 92.8, Black or African American 4.1, American Indian or Alaska Native 2.1, Multiple 1.0, Native Hawaiian or other Pacific Islander (0), Hispanic or Latino 38.1 | ertu 5mg QD + sita 100mg QD: 5.7 (5.0); ertu 15mg QD + sita 100mg QD: 6.5 (6.5); pbo: 6.8 (6.5) |
| Pratley RE 2014 | alo 12.5mg + met 500mg BID; alo 12.5mg + met 1000mg BID | 26 weeks | alo 12.5mg + met 500mg BID: 111; alo 12.5mg + met 1000mg BID: 114; pbo: 109 | alo 12.5mg + met 500mg BID: 53.7 (11.59); alo 12.5mg + met 1000mg BID: 54.6 (10.42); pbo: 53.1 (9.60) | alo 12.5mg + met 500mg BID: 43.2; alo 12.5mg + met 1000mg BID: 54.4; pbo: 50.5 | worldwide; not specified | alo 12.5mg + met 500mg BID: White 68.5, Asian 18.0, Black or African American 5.4, Other [American Indian or Alaska Native, Native Hawaiian or Other Pacific Islander and Multiracial 8.1; alo 12.5mg + met 1000mg BID: white 68.4, Asian 22.8, Black or African American 4.4, Other [American Indian or Alaska Native, Native Hawaiian or Other Pacific Islander and Multiracial 4.4; pbo: white 69.7, Asian 18.3, Black or African American 7.3, Other [American Indian or Alaska Native, Native Hawaiian or Other Pacific Islander and Multiracial 4.6 | alo 12.5mg + met 500mg BID: 4.1 (4.78); alo 12.5mg + met 1000mg BID: 4.2 (4.97); pbo: 4.3 (4.78) |

**Characteristics of Included Studies – Dual Therapy Initiation continued**

| **Study ID** | **Baseline A1C % (SD)** | **Mean Change in HbA1C % vs. baseline** | **Mean Difference in HbA1c % vs pbo** | **HYPO def** | **Ascertainment of Hypo** | **Rescue Medication** | **Excl of Pts w events? At screening or during study)** |
| --- | --- | --- | --- | --- | --- | --- | --- |
| Goldstein BJ 2007 | sita 50mg + met 500mg BID: 8.8 (1.0); sita 50mg + met 1000mg BID: 8.7 (0.9); pbo: 8.7 (1.0) | [LSM] sita 50mg + met 500mg BID: -1.40 (-1.56 to -1.24); sita 50mg + met 1000mg BID: -1.90 (-2.06 to -1.74); pbo: 0.17 (0.0 to 0.33) | [LSM] sita 50mg + met 500mg BID: -1.57 (-1.8 to -1.34); sita 50mg + met 1000mg BID: -2.07 (-2.30 to -1.84) | not reported | not reported | Rescue (glyburide) permitted. Safety and efficacy endpoints excluded data after rescue. | no |
| Haak T 2012 | lina 2.5mg BID + 500mg met BID: 8.7 (1.0); lina 2.5mg BID + 1000mg met BID: 8.7 (1.0); pbo: 8.7 (1.0) | adjusted mean (SE) lina 2.5mg BID + 500mg met BID: -1.2 (0.1); lina 2.5mg BID + 1000mg met BID: -1.6 (0.1); pbo: 0.1 (0.1) | adjusted mean (SE) lina 2.5mg BID + 500mg met BID: -1.3 (0.1); lina 2.5mg BID + 1000mg met BID: -1.7 (0.1) | Severe hypoglycemia defined as requiring the assistance of another person to actively administer carbohydrate, glucagon or other. | Hypoglycemic episodes were recorded and analyzed separately from other AEs. Hypoglycemic event intensity was graded according to the investigator’s discretion. | Rescue (with sulphonylureas, thiazolidinediones or insulin) permitted. Values obtained after rescue medication was initiated were not used in the LOCF. | unlikely, but hypoglycemic events graded at investigators discretion; criteria not specified and could lead to patient exclusion |
| Ji L 2016 | sita 50mg BID + met 500mg BID: 8.5 (1.0); sita 50mg BID + met 850mg BID: 8.6 (0.9); pbo: 9.0 (1.1) | [LSM] sita 50mg BID + met 500mg BID: -1.67 (-1.92 to -1.43); sita 50mg BID + met 850mg BID: -1.83 (-2.07 to -1.58); pbo: -0.59 (-0.84 to -0.34) | [LSM] sita 50mg BID + met 500mg BID: -1.08 (-1.39 to -0.78); sita 50mg BID + met 850mg BID: -1.24 (-1.55 to -0.93) | Any episode with symptoms consistent with hypoglycemia (e.g., weakness, dizziness, shakiness, increased sweating, palpitations or confusion) was reported as an episode of symptomatic hypoglycemia without a requirement for confirmatory blood glucose values. Asymptomatic hypoglycemia was defined as an episode without symptoms of hypoglycemia, but with fingerstick glucose level ≤3.9 mmol/L (≤70 mg/dL). Severe hypoglycemia was defined as any episode requiring assistance, either medical or non-medical. Episodes with a markedly depressed level of consciousness, loss of consciousness or seizure were to be classified as having required medical assistance, whether or not medical assistance was obtained. | not reported | Rescue therapy (glipizide) permitted. The primary approach to analyzing safety data treated data obtained after the initiation of rescue therapy as missing; a secondary approach included all data, regardless of rescue therapy. | no |
| Ji L 2017 | alo+met: 8.39 (0.81); pbo: 8.21 (0.77) | [LSM] alo+met: -1.53; pbo: -0.19 | [LSM] manually calculated alo+met: -1.34 | not reported | Self-monitor blood glucose levels, keep a hypoglycemic diary | Rescue confounding. Hyperglycemic rescue permitted. | no |
| Miller S 2018 | ertu 5mg QD + sita 100mg QD: 8.9 (0.9); erti 15mg QD + sita 100mg QD: 9.0 (0.9); pbo: 9.0 (0.9) | [LSM] ertu 5mg QD + sita 100mg QD: -1.6 (-1.8 to -1.4); ertu 15mg QD + sita 100mg QD: -1.7 (-1.9 to -1.5); pbo: -0.4 (-0.7 to -0.2) | [LSM] ertu 5mg QD + sita 100mg QD: -1.2 (-1.5 to -0.8); ertu 15mg QD + sita 100mg QD: -1.2 (-1.6 to -0.9) | Symptomatic  hypoglycemia (defined as episodes with  clinical symptoms reported by the investigator  as hypoglycemia; biochemical documentation  not required).  Documented hypoglycemia (symptomatic and asymptomatic), defined as episodes with a glucose level <70 mg/dL (3.89 mmol/L), were recorded. Severe hypoglycemia defined as requiring non-medical assistance. | not reported | Rescue confounding. Rescue therapy (glimepiride) permitted.  Data following initiation of glycemic rescue were included for the analysis of serious AEs (SAEs), deaths, and discontinuations due to AEs, and excluded for the other endpoints. | no |
| Pratley RE 2014 | not reported; The majority of patients (60%overall) entered with a baseline  HbA1c of 8.5% or lower. | [LSM] (SE) alo 12.5mg + met 500mg BID: -1.22 (0.094); alo 12.5mg + met 1000mg BID -1.55 (0.09); pbo 0.15 (n/a) | [LSM] manually calculated alo 12.5mg + met 500mg BID: -1.37; alo 12.5mg + met 1000mg BID -1.70 | Mild to moderate hypoglycemia (blood glucose <70 mg/dl (3.89 mmol/L), symptomatic or asymptomatic). All hypoglycemic episodes were associated with a blood glucose <70 mg/dl (3.89 mmol/l). Severe required assistance from another person. | Use of a home glucose  monitor and diary to record hypoglycemic episodes. | Rescue (SU or other) permitted. | no |

**Characteristics of Included Studies – Third AHA added to dual therapy background**

| **Study ID** | **Dose** | **Study Duration** | **n=** | **Mean Age (SD)** | **Gender (% male)** | **Countries Studied** | **Ethnicity (%)** | **Duration of Diabetes in Years (SD)** |
| --- | --- | --- | --- | --- | --- | --- | --- | --- |
| Dagogo-Jack S 2018 | ertu 5mg QD; ertu 15mg QD  (added to met plus sita) | 24 wks with extension until 52 weeks | ertu 5mg QD: 156; 15mg QD: 153; pbo: 153 | ertu 5mg QD: 59.2 (9.3); 15mg QD: 59.7 (8.6); pbo 58.3 (9.3) | ertu 5mg QD: 51.9; 15mg QD: 53.6; pbo 65.4 | USA, Argentina, Colombia, Czech Republic, Hungary, Israel, Romania, Slovakia, Republic of Korea (South Korea), Malaysia, Bulgaria, Finland | ertu 5mg QD: White 73.1, Asian 21.2, Black or African American 1.3, American Indian or Alaska Native 0.6, Multiple 3.8, Hispanic or Latino 14.7; 15mg QD: White 75.2, Asian 18.3, Black or African American 2.6, American Indian or Alaska Native 3.3, Multiple 0.7, Hispanic or Latino 16.3; pbo: White 70.6, Asian 21.6, Black or African American 2.0, American Indian or Alaska Native 3.3, Multiple 2.6, Hispanic or Latino 15.7 | ertu 5mg QD: 9.9 (6.1); 15mg QD: 9.2 (5.3); pbo 9.4 (5.6) |
| Han KA  2018 | ipra 50mg QD  (added to met plus sita) | 24 weeks | ipra 50mg QD: 73; pbo: 66 | ipra 50mg QD: 57.62 (8.26); pbo: 57.44 (7.88) | ipra 50mg QD: 50.7; pbo: 48.5 | Korea | Korean | ipra 50mg QD: 11.62 (5.89); pbo: 11.33 (6.63) |
| Jabbour SA 2014 | dapa 10mg QD  (added to met with or without sita) | 24 weeks | dapa 10mg: 223; pbo: 224 | dapa 10mg: 54.8 (10.4); pbo: 55.0 (10.2) | dapa 10mg: 57.0; pbo: 52.7 | Argentina, Germany, Mexico, Poland, UK, USA | dapa 10mg: White 72.2, Black 4.9, Asian 0.9, Other 22.0; pbo: White: 76.3, Black 2.7, Asian 0.9, Other 20.1 | dapa 10mg: 5.7 (4.87); pbo: 5.64 (5.40) |
| Ludvik B 2018 | dula 1.5mg QW; dula 0.75mg QW  (SGLT2i with or without metformin) | 24 weeks | dula 1.5mg QW: 142; dula 0.75mg QW: 141; pbo: 140 | dula 1.5mg QW: 56.17 (9.26); dula 0.75mg QW: 58.55 (9.14); pbo: 57.1 (9.59) | dula 1.5mg QW: 54.0; dula 0.75mg QW: 49; pbo: 47 | Spain, Isreal, US, Austria, | dula 1.5mg QW: White 89, American Indian or Alaska Native: 1, Asian 0, Black of African American 2, Multiple 8; dula 0.75mg QW: White 90, American Indian or Alaska Native: 1, Asian 1, Black of African American 2, Multiple 6; pbo: White 89, American Indian or Alaska Native 3, Asian 0, Black of African American 4, Multiple 4 | dula 1.5mg QW: 9.21 (5.74); dula 0.75mg QW: 10.05 (6.56); pbo 8.87 (6.13) |
| Mathieu C 2015 | dapa 10mg QD  (added to saxa plus met) | 24 weeks | dapa 10mg: 160; pbo: 160 | dapa 10mg: 55.2 (8.6); pbo 55.0 (9.6) | dapa 10mg: 43.7; pbo: 47.5 | USA, Puerto Rico, Romania, Russian Federation, Poland, Mexico, UK | dapa 10mg: White 93.8, African American 5.0, Asian 0.6, Other 0.6; pbo: White 91.9, African American 6.3, Asian 0.6, Other 1.3 | dapa 10mg: 7.2 (5.7); pbo: 8.0 (6.6) |
| Matthaei S 2015 | saxa 5mg/day QD  (added to dapa plus met) | 24 weeks | saxa 5mg: 153, pbo: 162 | saxa 5mg: 54.7 (9.8); pbo: 54.5 (9.3) | saxa 5mg: 47.7; pbo: 46.9 | USA, Puerto Rico, Canada, Romania, Russian Federation, Poland, Mexico, Czech Republic, Hungary | saxa 5mg: White 88.9, Black 7.2, Asian 3.3, Other 0.7; pbo: White 87.0, Black 5.6, Asian 4.9, Other 2.5 | saxa 5mg: 8.1 (7.0); pbo: 7.4 (5.8) |
| Rodbard HW 2016 | cana 100mg; cana 300mg  (added to sita plus met) | 26 weeks | cana 108; pbo 108 | cana: 57.4 (9.3); pbo: 57.5 (10.1) | cana: 61.7; pbo: 51.9 | 5 countries; not specified | cana: White 74.8, Black African American 5.6, Asian 18.7, Other [Native Hawaiian or other Pacific Islander and other] 0.9; pbo: White 72.6, Black African American 15.1, Asian 011.3, Other [Native Hawaiian or other Pacific Islander and other] 0.9 | cana: 9.8 (5.4); pbo: 10.1 (5.9) |
| Søfteland E 2017 | empa  (added to lina plus metformin)  single-pill combination  of empa 10mg/lina 5mg QD  or empa 25mg/lina 5mg QD  PLUS  unchanged background metformin | 24 weeks | empa 10mg: 112; empa 25mg: 110; pbo: 110 | empa 10mg: 54.3 (9.6); empa 25mg: 55.4 (9.9); pbo 55.9 (9.7) | empa 10mg: 60.6; empa 25mg: 64.5; pbo: 55.6 | Australia, Brazil, Canada, France, Korea, New Zealand, Norway, Spain, Taiwan, USA | empa 10mg: White 61.5, Asian 23.9, Other 14.7; empa 25mg: White 59.1, Asian 27.3, Other 13.6; pbo: White 54.6, Asian 29.6, Other 15.7 | empa 10mg: ≤1 year 5.5, >1-5 years 27.5, >5-10 years 38.5, >10 years 28.4; empa 25mg: ≤1 year 6.4, >1-5 years 37.3, >5-10 years 31.8, >10 years 24.5; pbo: ≤1 year 8.3, >1-5 years 28.7, >5-10 years 35.2, >10 years 27.8 |

**Characteristics of Included Studies – Third AHA added to dual therapy background continued**

| **Study ID** | **Baseline A1C % (SD)** | **Mean Change in HbA1C % vs. baseline** | **Mean Difference in HbA1c % vs pbo** | **HYPO def** | **Ascertainment of Hypo** | **Rescue Medication** | **Excl of Pts w events? At screening or during study)** |
| --- | --- | --- | --- | --- | --- | --- | --- |
| Dagogo-Jack S 2018 | ertu 5mg QD: 8.1 (0.9); 15mg QD: 8.0 (0.8); pbo: 8.0 (0.9) | [LSM] ertu 5mg: -0.8 (-0.9 to -0.6); 15mg: -0.9 (-1.0 to -0.7); Pbo: -0.1 (-0.2 to 0.0) | [LSM] ertu 5mg: -0.7 (-0.9 to -0.5); 15mg: -0.8 (-0.9 to -0.6) | Documented hypoglycaemia (symptomatic and asymptomatic), defined as episodes with a glucose level ≤3.9 mmol/L (70 mg/dL), with or without symptoms, was recorded. Severe hypoglycemia not defined. | not reported | Rescue (glimepiride or insulin) permitted. Data post rescue excluded for 24 weeks but not 52-week analysis, with the exception  of those related to hypoglycaemia. | no |
| Han KA  2018 | ipra 50mg QD: 7.90 (0.69);  pbo: 7.92 (0.79) | ipra 50mg:  -0.79 (0.59); pbo: 0.03 (0.84) | ipra 50mg: -0.83 (-1.07 to -0.59) | not reported | not reported | Rescue medication with glimepiride. | no |
| Jabbour SA 2014 | dapa 10mg: 7.9 (0.8); pbo: 8.0 (0.8) | [LSM] dapa 10mg: -0.5 (-0.6 to -0.4); pbo: 0.0 (-0.1 to 0.1) | [LSM] adjusted mean change from baseline (LOCF), placebo-corrected change: dapa 10mg: -0.5 (-0.6 to -0.3) | not reported | not reported | Rescue therapy (glimepiride) permitted. Change in hba1c as primary endpoint excluded data after rescue therapy; unclear whether safety data excluded data after rescue | no |
| Ludvik B 2018 | dula 1.5mg QW: 8.04 (0.65); dula 0.75mg QW: 8.04 (0.61); pbo: 8.05 (0.66) | [LSM] dula 1.5mg QW: -1.34 (SE 0.06); dula 0.75mg QW: -1.21 (SE 0.06); pbo: -0.54 (SE 0.06) | [LSM] dula 1.5mg QW vs pbo: -0.79 (-0.97 to -0.61); dula 0.75mg QW vs pbo: -0.66 (-0.84 to -0.49) | Total hypoglycaemia (plasma glucose ≤70 mg/dL [3∙9 mmol/L]). Severe Hypoglycaemia: An episode requiring the assistance of another person to actively administer carbohydrate, glucagon, or other resuscitative actions. These episodes may have been associated with sufficient neuroglycopenia to induce seizure or coma. Plasma glucose (PG) measurements may not have been available during such an event, but neurological recovery attributable to the restoration of PG to normal was considered sufficient evidence that the event was induced by a low PG concentration. Documented Symptomatic Hypoglycaemia: Any time a patient felt that he/she was experiencing symptoms and/or signs associated with hypoglycemia and had a PG level of ≤70 mg/dL (3∙9 mmol/L), or PG <54 mg/dL (3∙0 mmol/L). Asymptomatic Hypoglycaemia: Any event not accompanied by typical symptoms of hypoglycaemia but with a measured PG of ≤70 mg/dL (3∙9 mmol/L), or PG <54 mg/dL (3∙0 mmol/L). Nocturnal Hypoglycaemia: Any hypoglycemic event that occurred between bedtime and waking. Probable Symptomatic Hypoglycaemia: An event during which symptoms of hypoglycaemia were not accompanied by a PG determination (but that was presumably caused by a PG concentration of ≤70 mg/dL [3∙9 mmol/L]), or PG <54 mg/dL (3∙0 mmol/L). Total hypoglycaemia included any event that met criteria for severe, documented symptomatic, asymptomatic, nocturnal, or probable symptomatic hypoglycaemia as defined above. | documented a criterion | Rescue permitted. Oral AHA or insulin, results provided with and without rescue. | Patients who had severe persistent hyperglycemia or hypoglycaemia between study visits were advised to contact the investigative site. For repeated episodes of hypoglycaemia, the metformin dose could be reduced, proceeding to complete withdrawal, as deemed necessary by the investigator. Continued risk of hypoglycaemia, despite discontinuation of metformin, warranted dose reduction or complete withdrawal of the SGLT2 inhibitor. Patients were to continue injectable study drug in either case. |
| Mathieu C 2015 | dapa 10mg: 8.24 (0.96); pbo: 8.17 (0.98) | dapa 10mg: -0.82 (-0.96 to -0.69); pbo -0.10 (-0.24 to 0.04) | dapa 10mg: -0.72 (-0.91 to -0.53) | Hypoglycemia episodes were classified as minor (symptomatic or asymptomatic with plasma glucose concentration of <63 mg/dL (3.5 mmol/L), regardless of  the need for external assistance), major (symptomatic requiring third-party assistance because of severe impairment in consciousness or behavior with or without a plasma glucose concentration of <54 mg/dL (3.0 mmol/L) and prompt recovery after glucose or glucagon administration), and other (suggestive episode not meeting the criteria for major or minor). | not reported | Rescue permitted. Data after the receipt of rescue medication were excluded in safety analysis (one page after rescue). | no |
| Matthaei S 2015 | saxa 5mg: 7.97 (0.83); pbo: 7.86 (0.93) | saxa 5mg: -0.51 (-0.63 to -0.39); pbo: -0.16 (-0.28 to -0.04) | saxa 5mg: -0.35 (-0.52 to -0.18) | Hypoglycemic episodes were classified as minor (symptomatic or asymptomatic, with plasma glucose concentration <63 mg/dL (3.5 mmol/L), regardless of need for external assistance); major (symptomatic, requiring third-party assistance because of severe impairment in consciousness or behavior, with or without plasma glucose concentration <54 mg/dL (3.0 mmol/L), and prompt recovery after glucose or glucagon administration); and other (a suggestive episode not meeting the criteria for major or minor). | not reported | Rescue therapy permitted. Primary efficacy (change in baseline hba1c) excluded data after rescue. Whether safety analysis excluded data after rescue was not addressed. | no |
| Rodbard HW 2016 | cana: 8.5 (0.9); pbo: 8.4 (0.8) | [LSM] cana: -0.91; pbo: -0.01 (CI n/a) | [LSM] cana: -0.89 (-1.19 to -0.59) | Documented hypoglycaemia [i.e. concurrent fingerstick or plasma glucose ≤3.9mmol/l (≤70mg/dl) with or without symptoms or severe episodes (i.e. requiring assistance from another person or resulting in seizure or loss of consciousness)]. | Patients were instructed to record information on the signs and symptoms of hypoglycaemia,  as well as associated SMBG measurements, if available. | Rescue not permitted and patients were discontinued from the study if requiring rescue. | no |
| Søfteland E 2017 | empa 10mg: 7.97 (0.84); empa 25mg: 7.97 (0.82); pbo: 7.97 (0.85) | empa 10mg: -0.65 (n/a); empa 25mg: -0.56 (n/a); pbo: 0.14 | empa 10mg: -0.79 (-1.02 to -0.55); empa 25mg: -0.70 (-0.93 to -0.46) | Confirmed hypoglycemic AEs (plasma glucose values ≤3.9 mmol/L and/or requiring assistance) | not reported | Rescue therapy permitted (but not DPP4i, GLP1RA or SGLT2i). Data post rescue set to missing for all primary analysis. | no |

**Supplemental Figure 1: Risk Ratio of Any Hypoglycemia**

(only forest plots containing ≥ 2 studies shown)

**
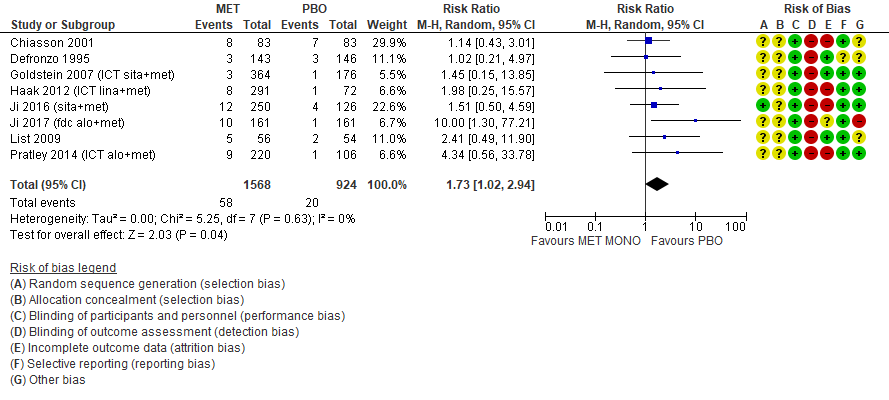
**

**
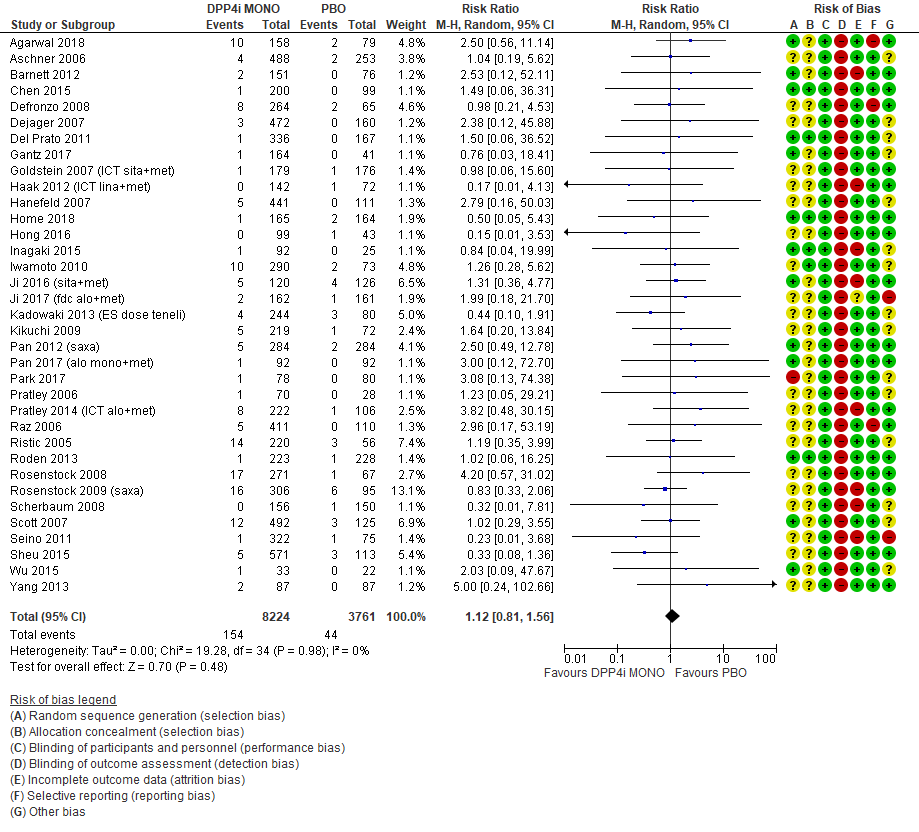
**

**
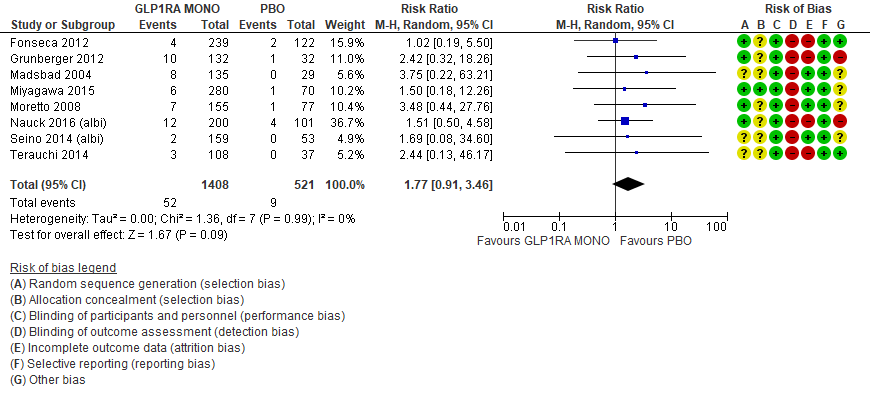
**

**
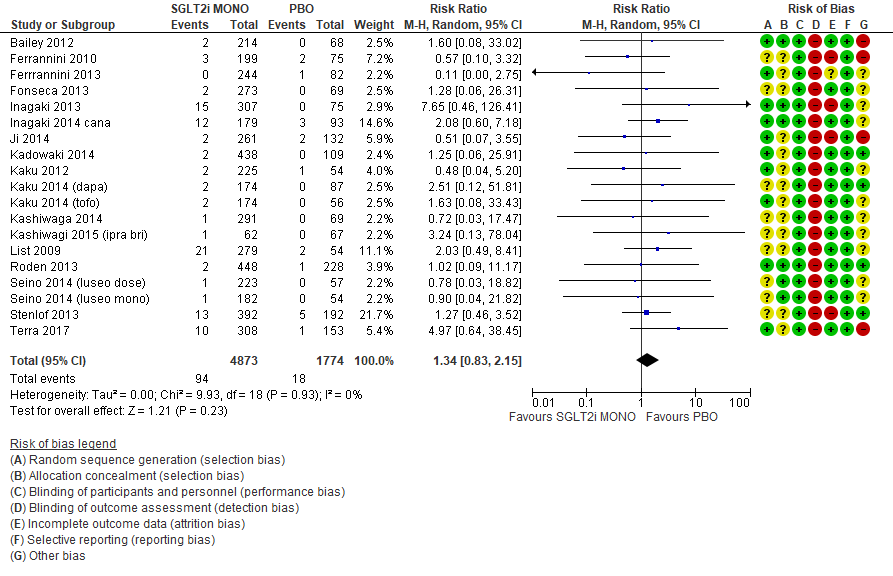
**

**
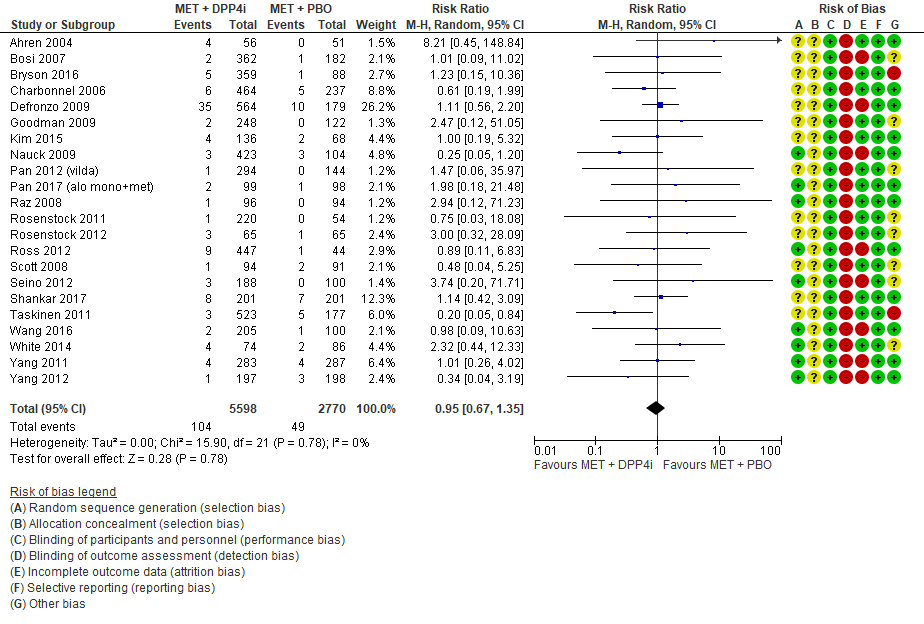
**

**
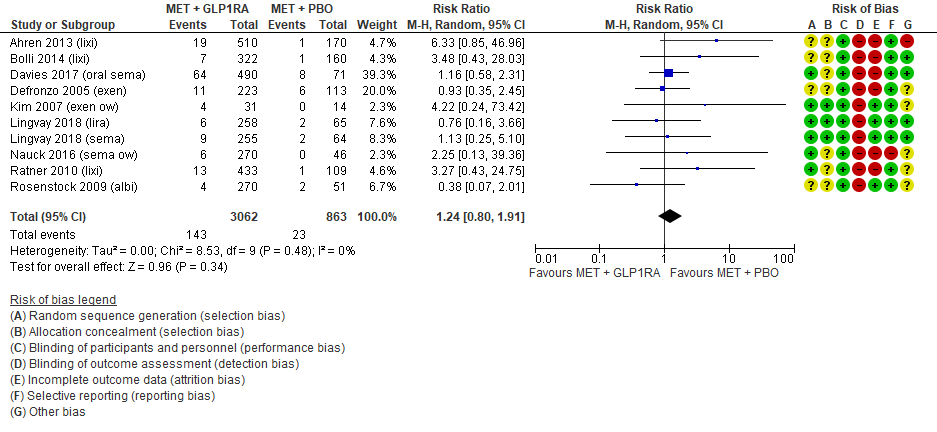
**

**
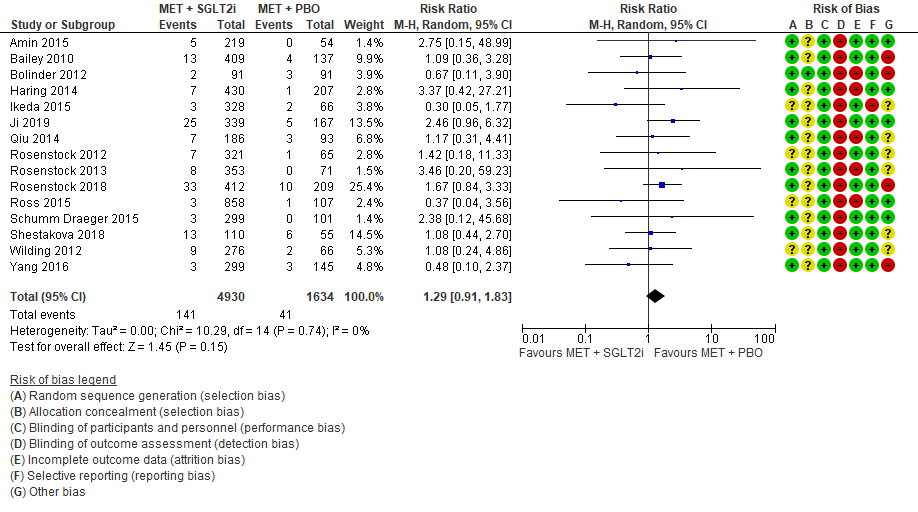
**

**
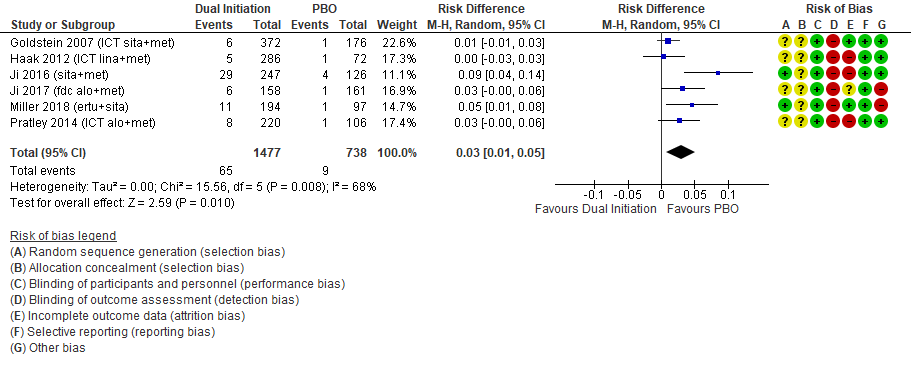
**

**
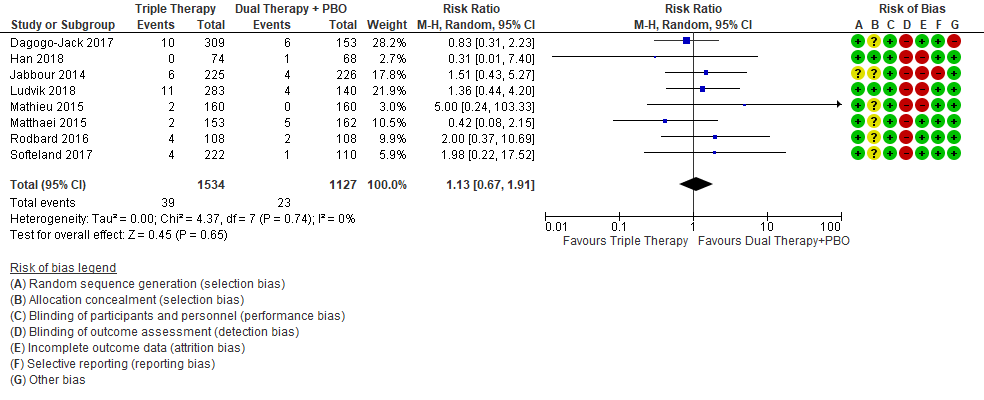
**

**Supplemental Figure 2: Risk Ratio of Severe Hypoglycemia**

(only forest plots containing ≥ 2 studies shown)

**
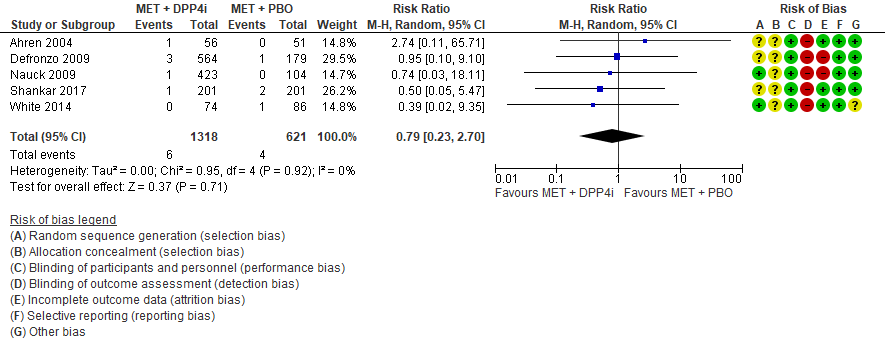
**

**
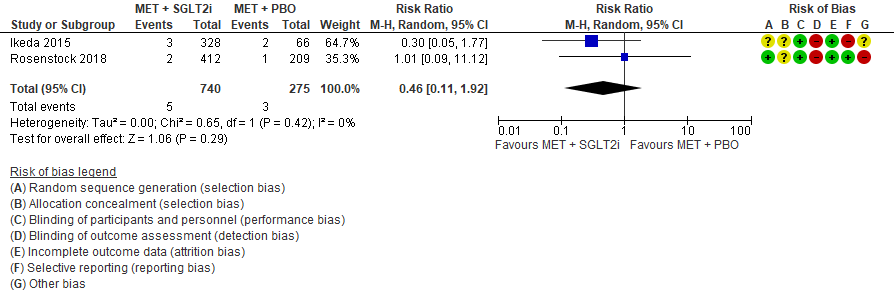
**

**
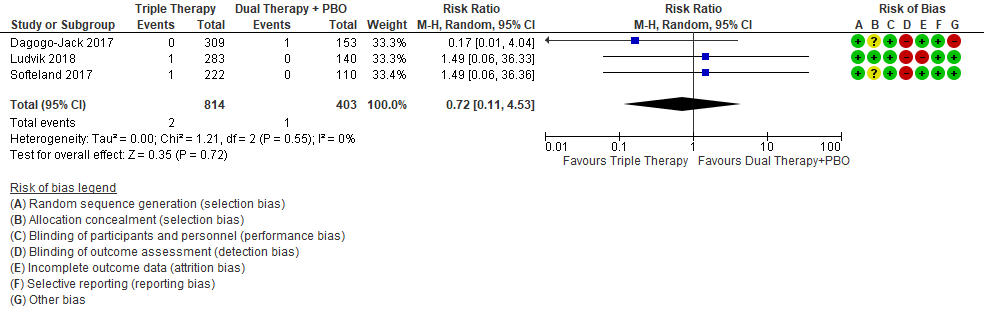
**

Supplemental Figure 3

(only funnel plots of ≥10 more studies shown)

Funnel Plot of DPP4i Monotherapy - Any Hypoglycemia

**
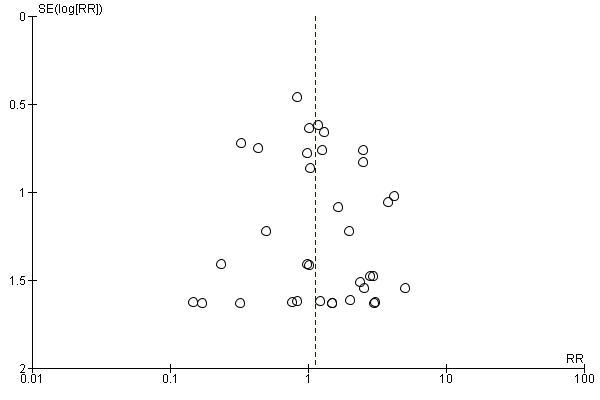
**

Funnel Plot of SGLT2i Monotherapy - Any Hypoglycemia

**
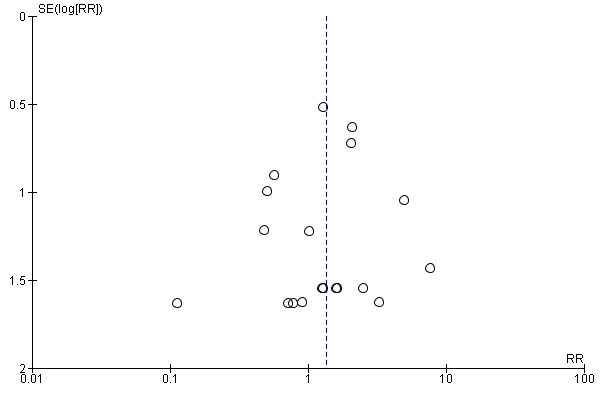
**

Funnel Plot of DPP4i Added to Metformin Background - Any Hypoglycemia


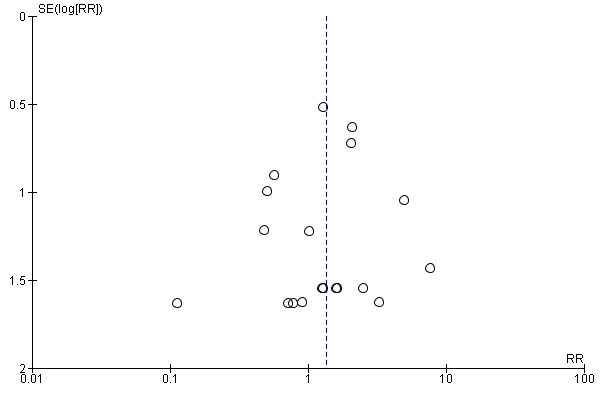


Funnel Plot of GLP1RA Added to Metformin Background - Any Hypoglycemia

**
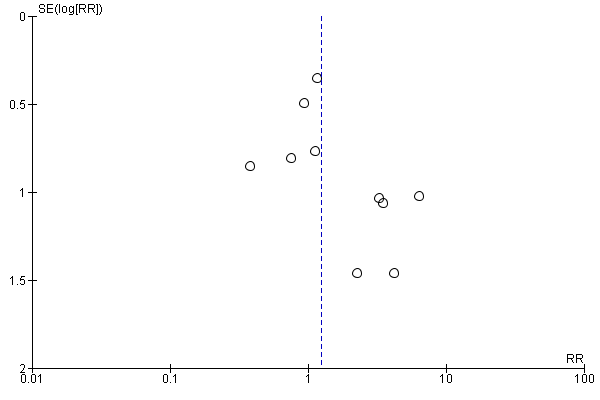
**

Funnel Plot of SGLT2i Added to Metformin Background - Any Hypoglycemia

**
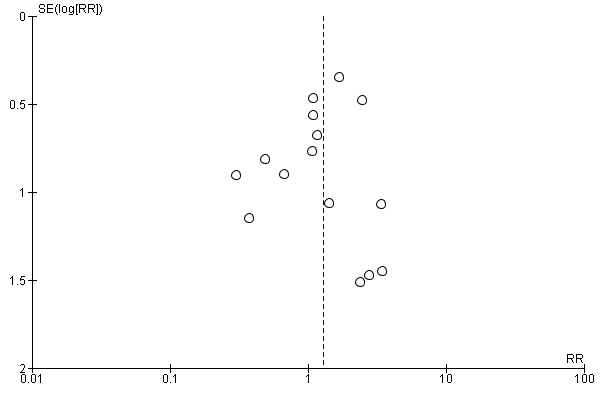
**

**Supplemental Figure 4:** **Risk Difference of Any Hypoglycemia**

**
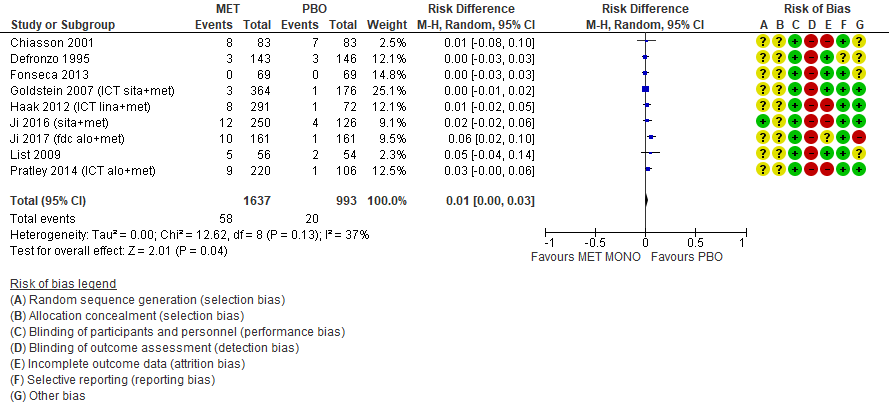
**

**
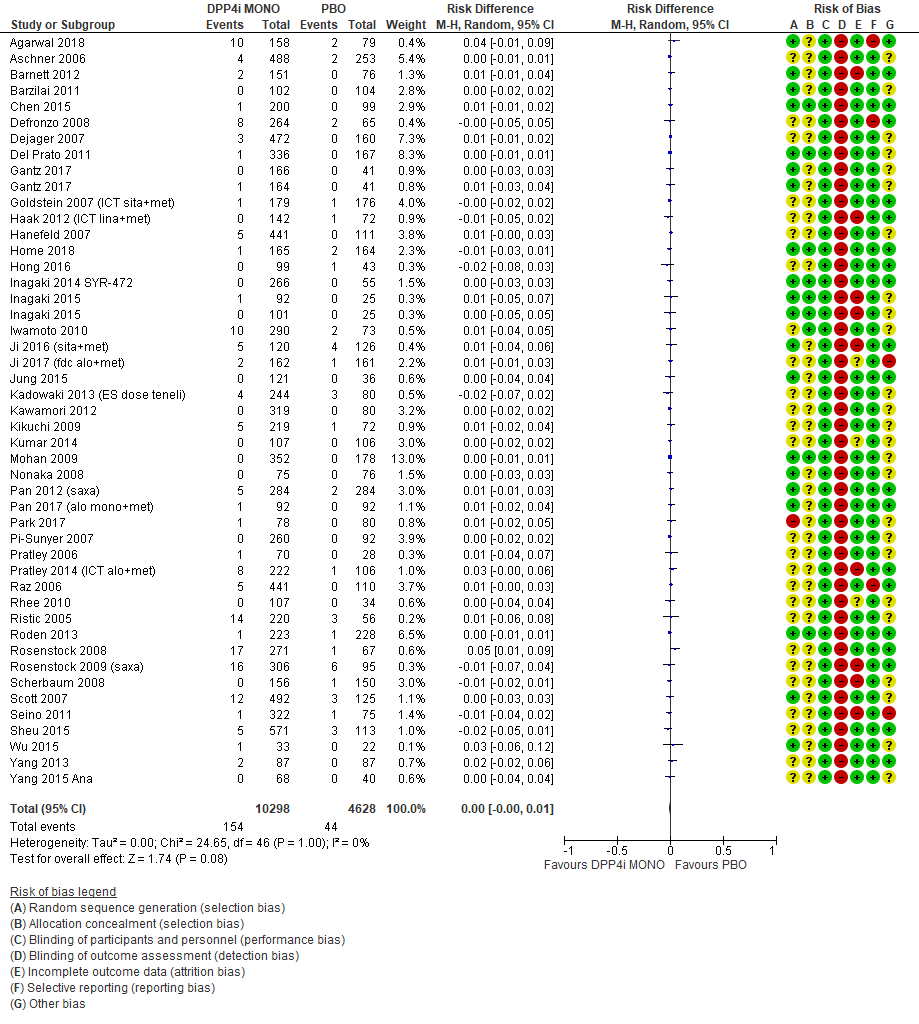
**

**
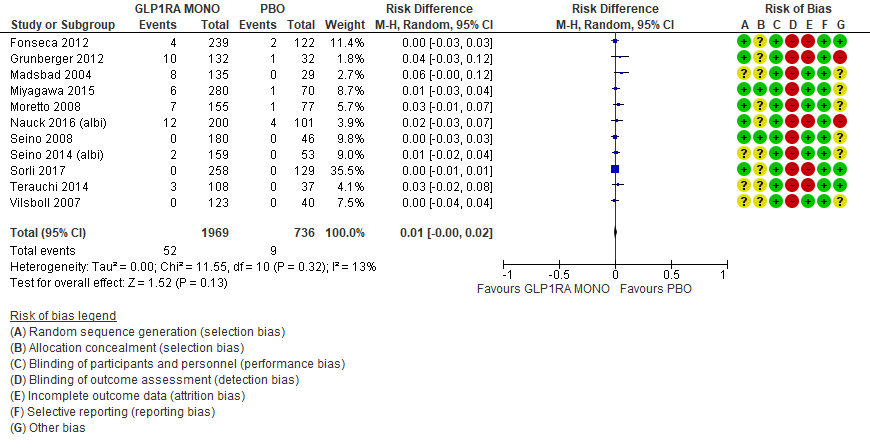
**

**
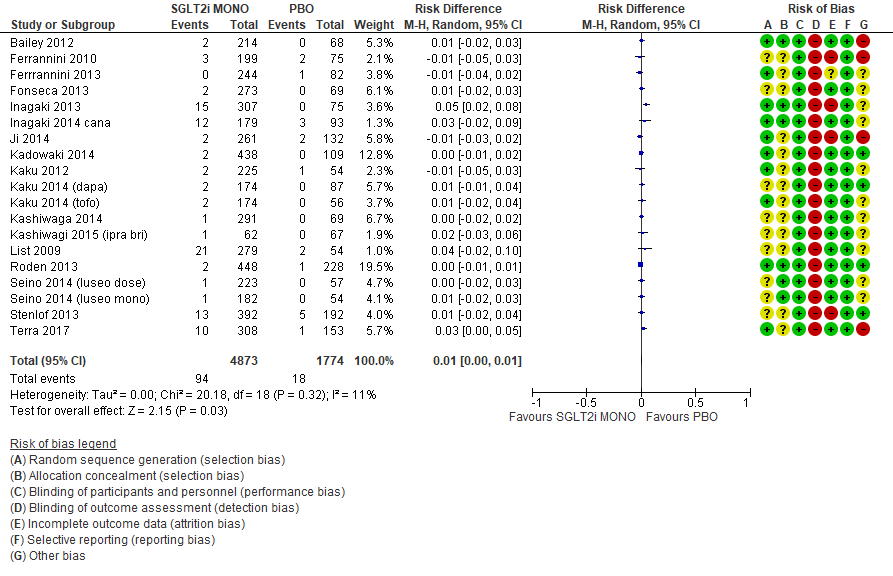
**

**
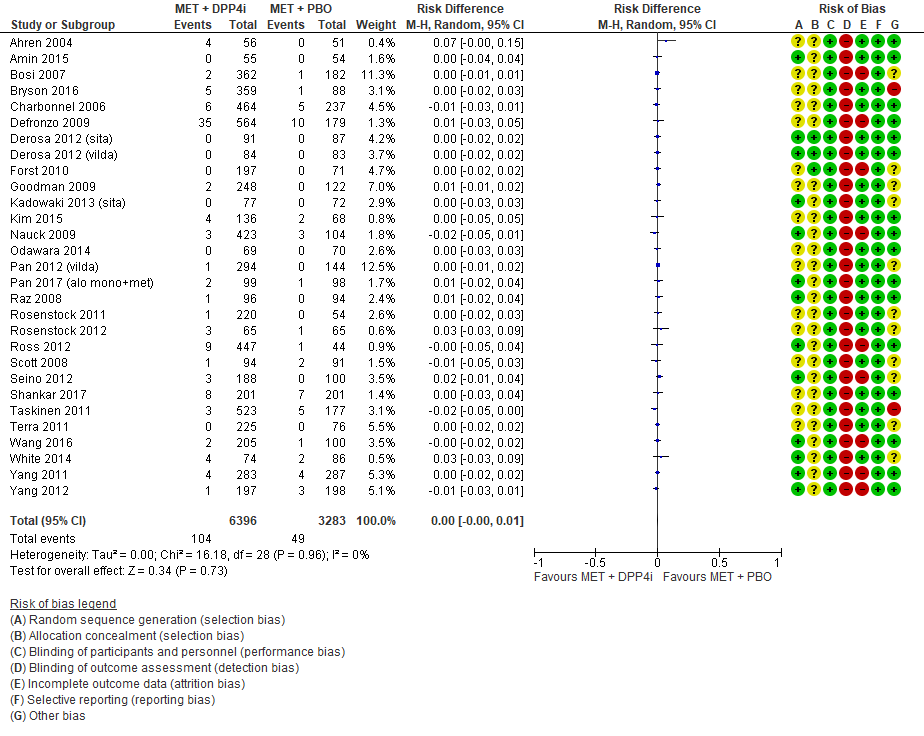
**

**
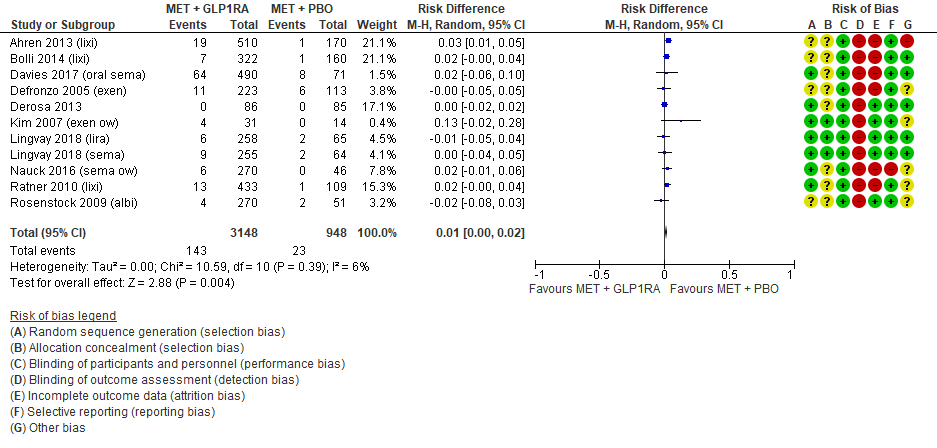
**

**
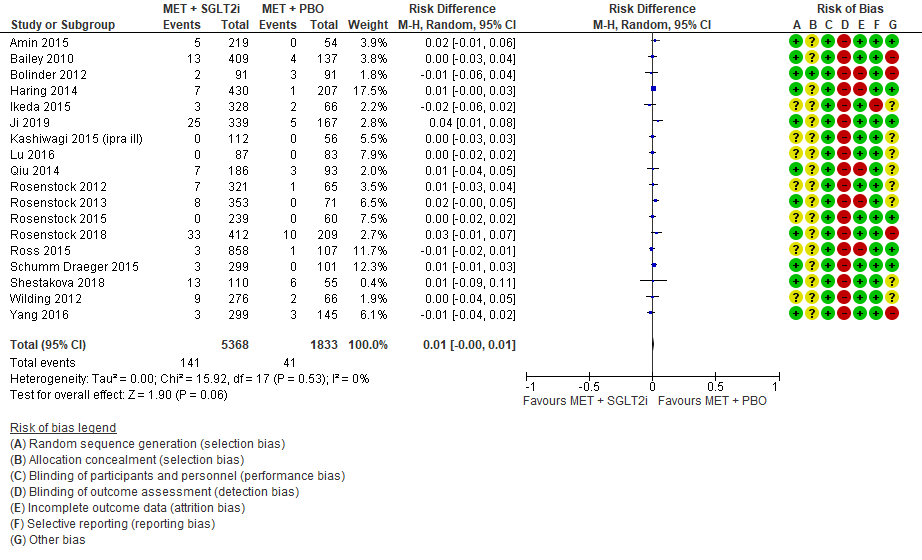
**

**
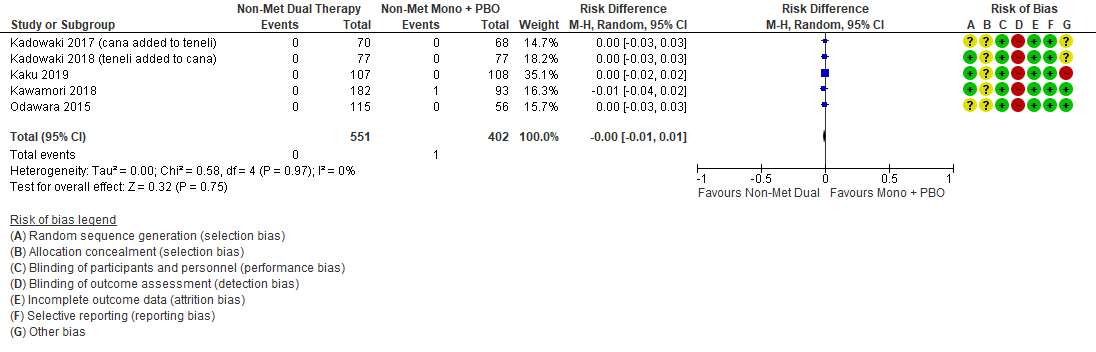
**

**
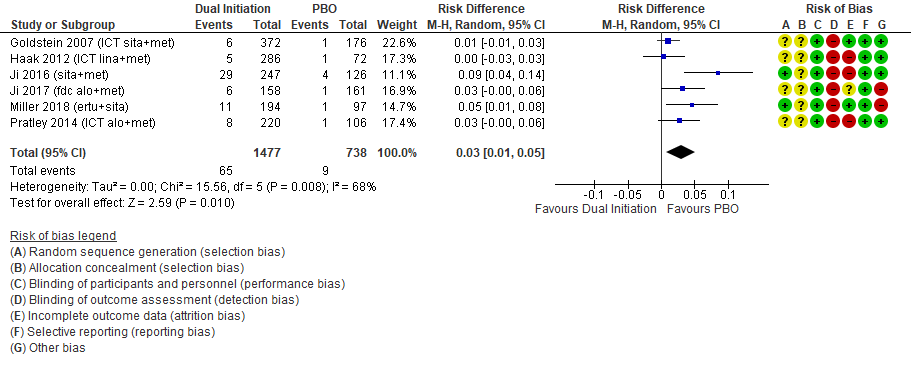
**

**
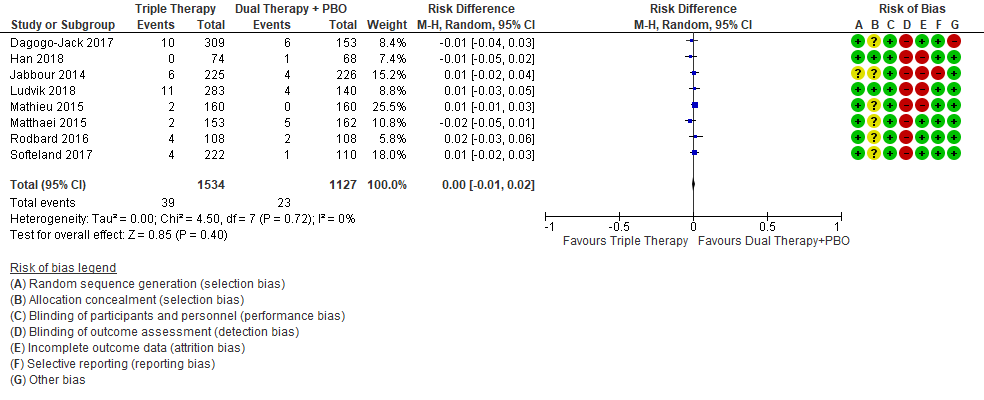
**

**Supplemental Figure 5: Risk Difference of Severe Hypoglycemia**

**
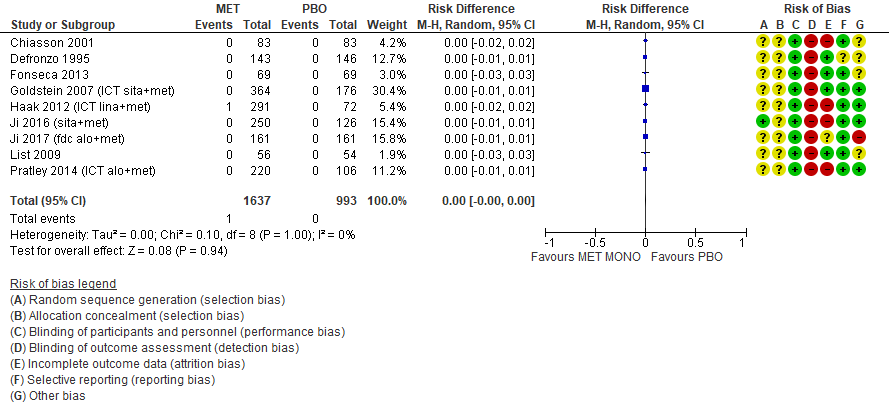
**

**
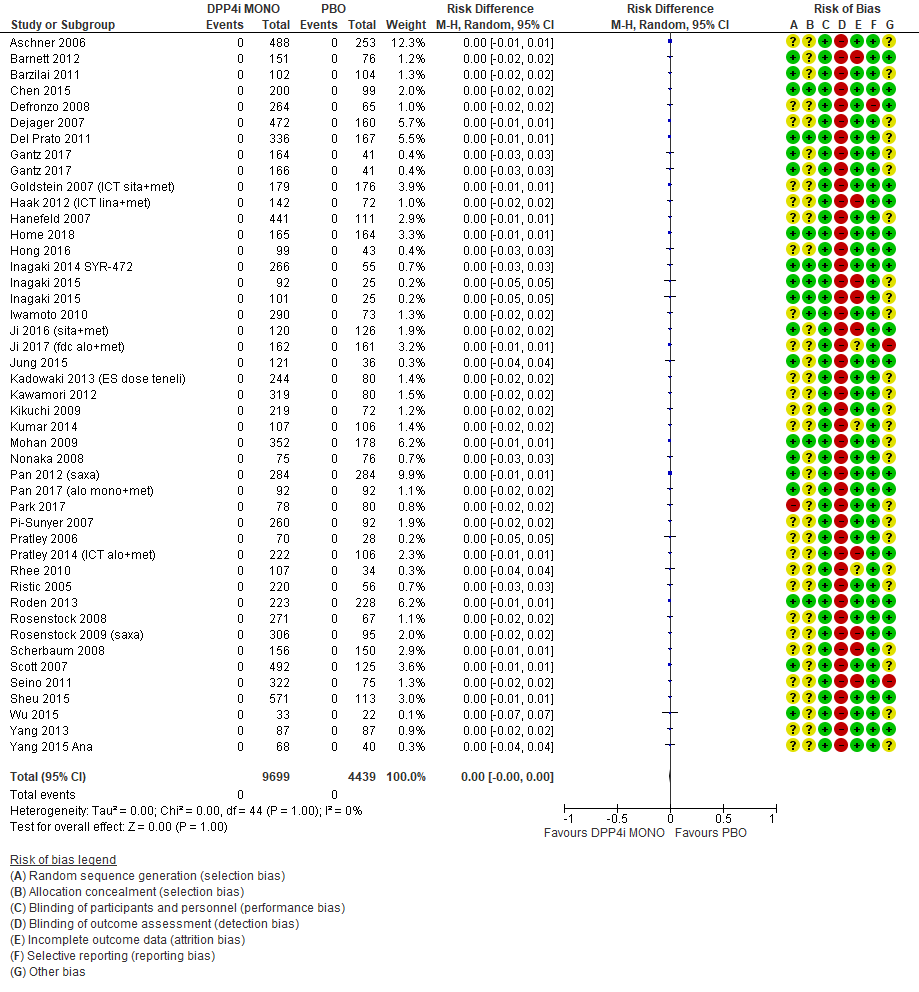
**

**
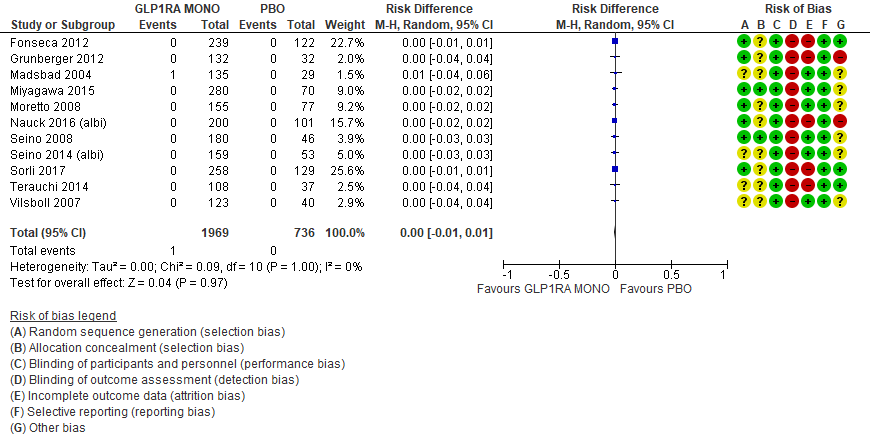
**

**
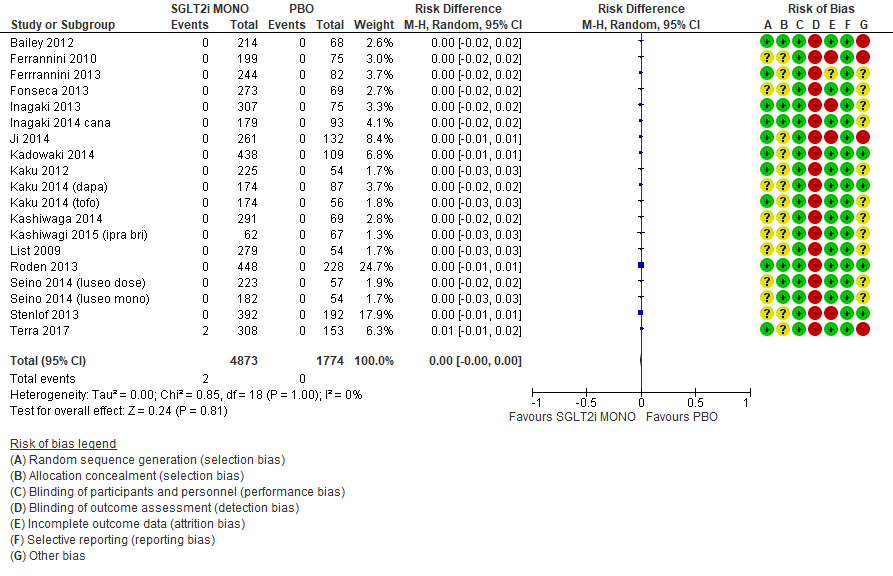
**

**
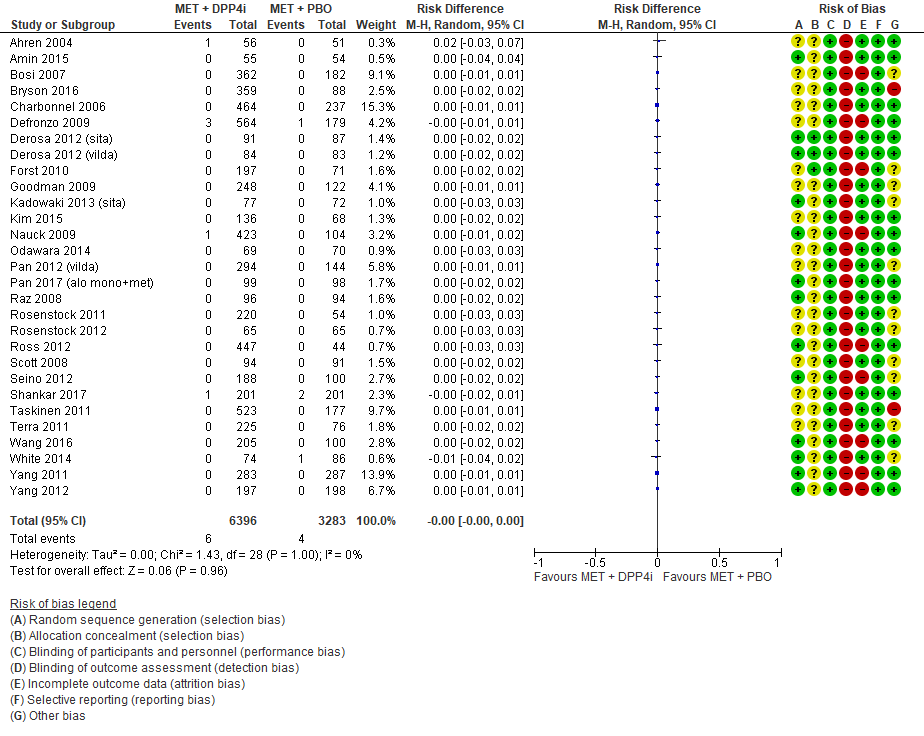
**

**
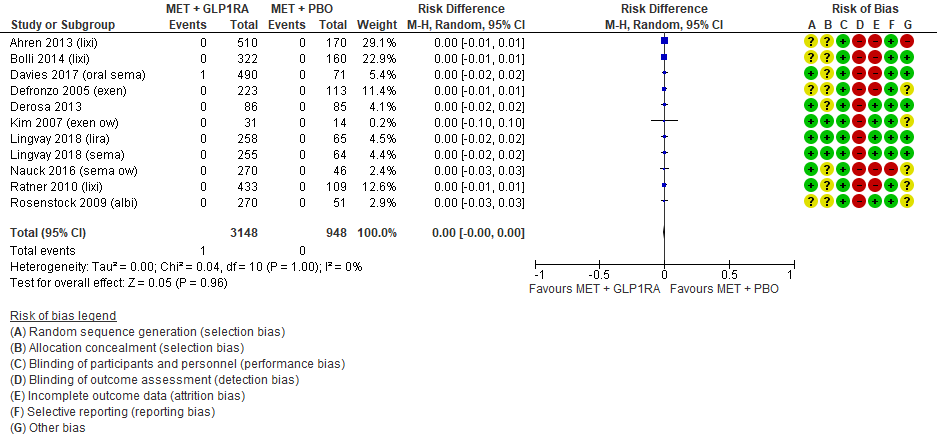
**

**
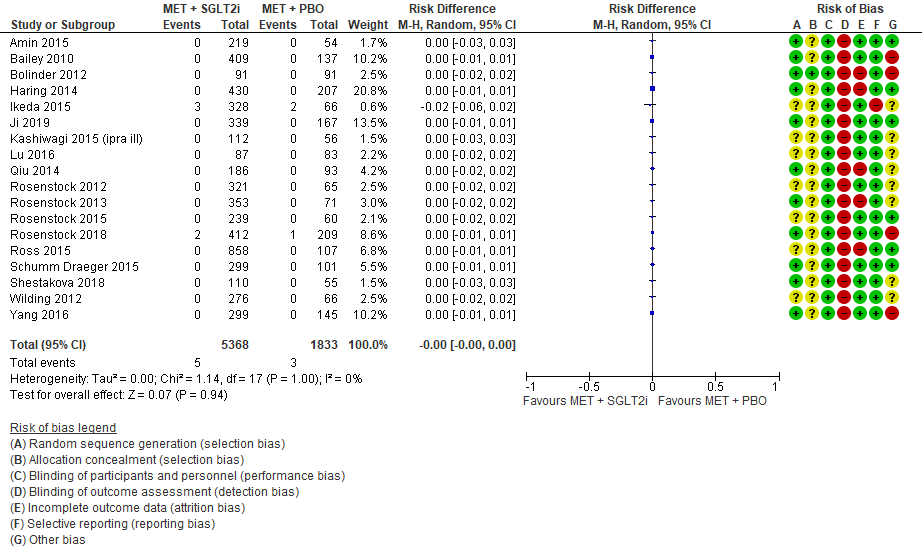
**

**
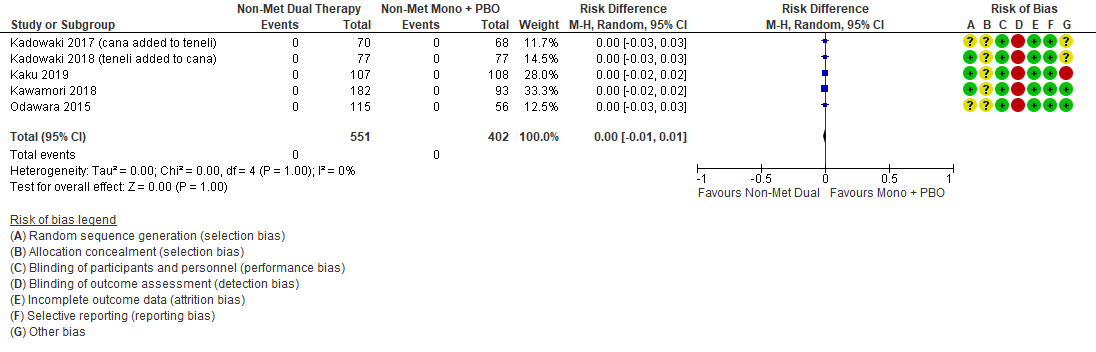
**

**
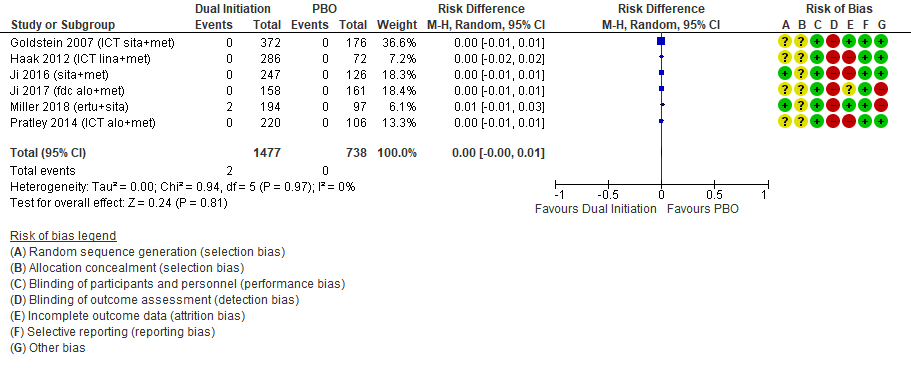
**

**
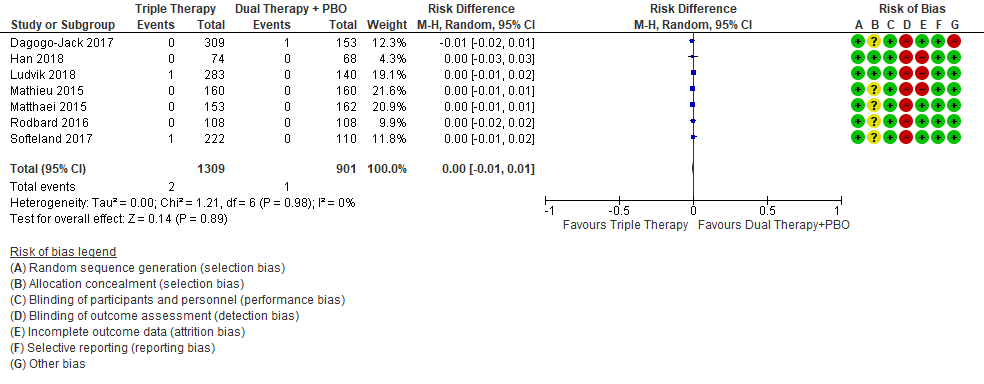
**

**Supplemental Table 3: Post Hoc Analysis if SH was ≤ 3.1 mmol/L**

**1 Monotherapy: Met vs PBO**

| **Outcome or Subgroup** | **Studies** | **Participants** | **Statistical Method** | **Effect Estimate** |
| --- | --- | --- | --- | --- |

| 1.1 ANY HYPO | 8 | 2492 | Risk Ratio (M-H, Random, 95% CI) | 1.73 [1.02, 2.94] |
| --- | --- | --- | --- | --- |
| 1.2 ANY HYPO (w BA0E) | 9 | 2630 | Risk Difference (M-H, Random, 95% CI) | 0.01 [0.00, 0.03] |

| 1.3 SH | 1 | 363 | Risk Ratio (M-H, Random, 95% CI) | 0.75 [0.03, 18.22] |
| --- | --- | --- | --- | --- |
| 1.4 SH (w BA0E) | 9 | 2630 | Risk Difference (M-H, Random, 95% CI) | 0.00 [-0.00, 0.00] |

**2 Monotherapy: DPP4i vs PBO**

| **Outcome or Subgroup** | **Studies** | **Participants** | **Statistical Method** | **Effect Estimate** |
| --- | --- | --- | --- | --- |

| 2.1 ANY HYPO | 35 | 11985 | Risk Ratio (M-H, Random, 95% CI) | 1.12 [0.81, 1.56] |
| --- | --- | --- | --- | --- |
| 2.2 ANY HYPO (w BAOE) | 45 | 14926 | Risk Difference (M-H, Random, 95% CI) | 0.00 [-0.00, 0.01] |

| 2.3 SH | 3 | 1036 | Risk Ratio (M-H, Random, 95% CI) | 1.03 [0.17, 6.15] |
| --- | --- | --- | --- | --- |
| 2.4 SH (w BA0E) | 43 | 14138 | Risk Difference (M-H, Random, 95% CI) | 0.00 [-0.00, 0.00] |

**3 Monotherapy: GLP1RA vs PBO**

| **Outcome or Subgroup** | **Studies** | **Participants** | **Statistical Method** | **Effect Estimate** |
| --- | --- | --- | --- | --- |

| 3.1 ANY HYPO | 8 | 1929 | Risk Ratio (M-H, Random, 95% CI) | 1.77 [0.91, 3.46] |
| --- | --- | --- | --- | --- |
| 3.2 ANY HYPO (w BA0E) | 11 | 2705 | Risk Difference (M-H, Random, 95% CI) | 0.01 [-0.00, 0.02] |

| 3.3 SH | 1 | 164 | Risk Ratio (M-H, Random, 95% CI) | 0.66 [0.03, 15.85] |
| --- | --- | --- | --- | --- |
| 3.4 SH (w BA0E) | 11 | 2705 | Risk Difference (M-H, Random, 95% CI) | 0.00 [-0.01, 0.01] |

**4 Monotherapy: SGLT2i vs PBO**

| **Outcome or Subgroup** | **Studies** | **Participants** | **Statistical Method** | **Effect Estimate** |
| --- | --- | --- | --- | --- |

| 4.1 ANY HYPO | 19 | 6647 | Risk Ratio (M-H, Random, 95% CI) | 1.34 [0.83, 2.15] |
| --- | --- | --- | --- | --- |
| 4.2 ANY HYPO (w BA0E) | 19 | 6647 | Risk Difference (M-H, Random, 95% CI) | 0.01 [0.00, 0.01] |

| 4.3 SH | 1 | 461 | Risk Ratio (M-H, Random, 95% CI) | 2.49 [0.12, 51.59] |
| --- | --- | --- | --- | --- |
| 4.4 SH (w BA0E) | 19 | 6647 | Risk Difference (M-H, Random, 95% CI) | 0.00 [-0.00, 0.00] |

**5 Metformin Background: DPP4i vs PBO**

| **Outcome or Subgroup** | **Studies** | **Participants** | **Statistical Method** | **Effect Estimate** |
| --- | --- | --- | --- | --- |

| 5.1 ANY HYPO | 22 | 8368 | Risk Ratio (M-H, Random, 95% CI) | 0.95 [0.67, 1.35] |
| --- | --- | --- | --- | --- |
| 5.2 ANY HYPO (w BA0E) | 29 | 9679 | Risk Difference (M-H, Random, 95% CI) | 0.00 [-0.00, 0.01] |

| 5.3 SH | 8 | 3291 | Risk Ratio (M-H, Random, 95% CI) | 1.10 [0.42, 2.89] |
| --- | --- | --- | --- | --- |
| 5.4 SH (w BA0E) | 29 | 9679 | Risk Difference (M-H, Random, 95% CI) | 0.00 [-0.00, 0.00] |

**6 Metformin Background: GLP1RA vs PBO**

| **Outcome or Subgroup** | **Studies** | **Participants** | **Statistical Method** | **Effect Estimate** |
| --- | --- | --- | --- | --- |

| 6.1 ANY HYPO | 10 | 3925 | Risk Ratio (M-H, Random, 95% CI) | 1.24 [0.80, 1.91] |
| --- | --- | --- | --- | --- |
| 6.2 ANY HYPO (w BA0E) | 11 | 4096 | Risk Difference (M-H, Random, 95% CI) | 0.01 [0.00, 0.02] |

| 6.3 SH | 5 | 1564 | Risk Ratio (M-H, Random, 95% CI) | 1.03 [0.46, 2.31] |
| --- | --- | --- | --- | --- |
| 6.4 SH (w BA0E) | 11 | 4096 | Risk Difference (M-H, Random, 95% CI) | 0.00 [-0.00, 0.01] |

**7 Metformin Background: SGLT2i vs PBO**

| **Outcome or Subgroup** | **Studies** | **Participants** | **Statistical Method** | **Effect Estimate** |
| --- | --- | --- | --- | --- |

| 7.1 ANY HYPO | 15 | 6564 | Risk Ratio (M-H, Random, 95% CI) | 1.29 [0.91, 1.83] |
| --- | --- | --- | --- | --- |
| 7.2 ANY HYPO (w BA0E) | 18 | 7201 | Risk Difference (M-H, Random, 95% CI) | 0.01 [-0.00, 0.01] |

| 7.3 SH | 2 | 1015 | Risk Ratio (M-H, Random, 95% CI) | 0.46 [0.11, 1.92] |
| --- | --- | --- | --- | --- |
| 7.4 SH (w BA0E) | 18 | 7201 | Risk Difference (M-H, Random, 95% CI) | -0.00 [-0.00, 0.00] |

**8 Non-Metformin Background: Dual Therapy vs Monotherapy + PBO**

| **Outcome or Subgroup** | **Studies** | **Participants** | **Statistical Method** | **Effect Estimate** |
| --- | --- | --- | --- | --- |

| 8.1 ANY HYPO | 1 | 275 | Risk Ratio (M-H, Random, 95% CI) | 0.17 [0.01, 4.16] |
| --- | --- | --- | --- | --- |
| 8.2 ANY HYPO (w BA0E) | 5 | 953 | Risk Difference (M-H, Random, 95% CI) | -0.00 [-0.01, 0.01] |

| 8.3 SH | 0 | 0 | Risk Ratio (M-H, Random, 95% CI) | Not estimable |
| --- | --- | --- | --- | --- |
| 8.4 SH (w BA0E) | 5 | 953 | Risk Difference (M-H, Random, 95% CI) | 0.00 [-0.01, 0.01] |

**9 Dual Initiation vs PBO**

| **Outcome or Subgroup** | **Studies** | **Participants** | **Statistical Method** | **Effect Estimate** |
| --- | --- | --- | --- | --- |

| 9.1 ANY HYPO | 6 | 2215 | Risk Ratio (M-H, Random, 95% CI) | 3.56 [1.79, 7.10] |
| --- | --- | --- | --- | --- |
| 9.2 ANY HYPO (w BA0E) | 6 | 2215 | Risk Difference (M-H, Random, 95% CI) | 0.03 [0.01, 0.05] |

| 9.3 SH | 1 | 291 | Risk Ratio (M-H, Random, 95% CI) | 2.51 [0.12, 51.83] |
| --- | --- | --- | --- | --- |
| 9.4 SH (w BA0E) | 6 | 2215 | Risk Difference (M-H, Random, 95% CI) | 0.00 [-0.00, 0.01] |

**10 Triple Therapy vs Dual Therapy + PBO**

| **Outcome or Subgroup** | **Studies** | **Participants** | **Statistical Method** | **Effect Estimate** |
| --- | --- | --- | --- | --- |

| 10.1 ANY HYPO | 8 | 2661 | Risk Ratio (M-H, Random, 95% CI) | 1.13 [0.67, 1.91] |
| --- | --- | --- | --- | --- |
| 10.2 ANY HYPO (w BA0E) | 8 | 2661 | Risk Difference (M-H, Random, 95% CI) | 0.00 [-0.01, 0.02] |

| 10.3 SH | 3 | 1217 | Risk Ratio (M-H, Random, 95% CI) | 0.72 [0.11, 4.53] |
| --- | --- | --- | --- | --- |
| 10.4 SH (w BA0E) | 7 | 2210 | Risk Difference (M-H, Random, 95% CI) | 0.00 [-0.01, 0.01] |

**Supplemental Table 4: Summary of Findings Table (SoF)**

**Question**: Monotherapy: Met compared to PBO in patients with T2D

| **Certainty assessment** | | | | | | | **№ of patients** | | **Effect** | | **Certainty** |
| --- | --- | --- | --- | --- | --- | --- | --- | --- | --- | --- | --- |
| **№ of studies** | **Study design** | **Risk of bias** | **Inconsistency** | **Indirectness** | **Imprecision** | **Other considerations** | **Monotherapy: Met** | **PBO** | **Relative (95% CI)** | **Absolute (95% CI)** |  |
| Any Hypoglycemia | | | | | | | | | | | |
| 8 | randomised trials | very serious ^a,b^ | not serious | not serious | not serious | none | 58/1568 (3.7%) | 20/924 (2.2%) | **RR 1.73** (1.02 to 2.94) | **16 more per 1,000** (from 0 fewer to 42 more) | ⨁⨁◯◯ LOW |
| Any Hypoglycemia (including studies with both arm zero events) | | | | | | | | | | | |
| 9 | randomised trials | very serious ^a,b^ | not serious | not serious | not serious | none | 58/1637 (3.5%) | 20/993 (2.0%) | not estimable | **10 fewer per 1,000** (from 30 fewer to 0 fewer) | ⨁⨁◯◯ LOW |
| Severe Hypoglycemia | | | | | | | | | | | |
| 1 | randomised trials | not serious | not serious | not serious | not serious ^c^ | none | 1/291 (0.3%) | 0/72 (0.0%) | **RR 0.75** (0.03 to 18.22) | **0 fewer per 1,000** (from 0 fewer to 0 fewer) | ⨁⨁⨁⨁ HIGH |
| Severe Hypoglycemia (including studies with both arm zero events) | | | | | | | | | | | |
| 9 | randomised trials | not serious | not serious | not serious | not serious | none | 1/1637 (0.1%) | 0/993 (0.0%) | not estimable | **0 fewer per 1,000** (from 0 fewer to 0 fewer) | ⨁⨁⨁⨁ HIGH |

**CI:** Confidence interval; **RR:** Risk ratio

**Explanations**

a. Harms outcome in a placebo-controlled study

b. Attrition bias

c. Despite single study, 8 others reported zero severe events

**Question**: Monotherapy: DPP4i compared to PBO in patients with T2D

| **Certainty assessment** | | | | | | | **№ of patients** | | **Effect** | | **Certainty** |
| --- | --- | --- | --- | --- | --- | --- | --- | --- | --- | --- | --- |
| **№ of studies** | **Study design** | **Risk of bias** | **Inconsistency** | **Indirectness** | **Imprecision** | **Other considerations** | **Monotherapy: DPP4i** | **PBO** | **Relative (95% CI)** | **Absolute (95% CI)** |  |
| Any Hypoglycemia | | | | | | | | | | | |
| 35 | randomised trials | serious ^a^ | not serious | not serious | not serious | none | 154/8224 (1.9%) | 44/3761 (1.2%) | **RR 1.12** (0.81 to 1.56) | **1 more per 1,000** (from 2 fewer to 7 more) | ⨁⨁⨁◯ MODERATE |
| Any Hypoglycemia (including studies with both arm zero events) | | | | | | | | | | | |
| 45 | randomised trials | serious ^a^ | not serious | not serious | not serious | none | 154/10298 (1.5%) | 44/4628 (1.0%) | not estimable | **0 fewer per 1,000** (from 10 fewer to 0 fewer) | ⨁⨁⨁◯ MODERATE |
| Severe Hypoglycemia | | | | | | | | | | | |
| 0 | randomised trials | not serious | not serious | not serious | not serious ^b^ | none | 0/0 | 0/0 | not pooled | see comment | ⨁⨁⨁⨁ HIGH |
| Severe Hypoglycemia (including studies with both arm zero events) | | | | | | | | | | | |
| 43 | randomised trials | not serious | not serious | not serious | not serious | none | 0/9699 (0.0%) | 0/4439 (0.0%) | not estimable | **0 fewer per 1,000** (from 0 fewer to 0 fewer) | ⨁⨁⨁⨁ HIGH |

**CI:** Confidence interval; **RR:** Risk ratio

**Explanations**

a. Harms outcome in a placebo-controlled study

b. Despite zero studies, 43 studies others reported zero severe events

**Question**: Monotherapy: GLP1RA compared to PBO in patients with T2D

| **Certainty assessment** | | | | | | | **№ of patients** | | **Effect** | | **Certainty** |
| --- | --- | --- | --- | --- | --- | --- | --- | --- | --- | --- | --- |
| **№ of studies** | **Study design** | **Risk of bias** | **Inconsistency** | **Indirectness** | **Imprecision** | **Other considerations** | **Monotherapy: GLP1RA** | **PBO** | **Relative (95% CI)** | **Absolute (95% CI)** |  |
| Any Hypoglycemia | | | | | | | | | | | |
| 8 | randomised trials | very serious ^a,b^ | not serious | not serious | not serious | none | 52/1408 (3.7%) | 9/521 (1.7%) | **RR 1.77** (0.91 to 3.46) | **13 more per 1,000** (from 2 fewer to 42 more) | ⨁⨁◯◯ LOW |
| Any Hypoglycemia (including studies with both arm zero events) | | | | | | | | | | | |
| 11 | randomised trials | very serious ^a,b^ | not serious | not serious | not serious | none | 52/1969 (2.6%) | 9/736 (1.2%) | not estimable | **10 fewer per 1,000** (from 20 fewer to 0 fewer) | ⨁⨁◯◯ LOW |
| Severe Hypoglycemia | | | | | | | | | | | |
| 1 | randomised trials | not serious | not serious | not serious | not serious ^c^ | none | 1/135 (0.7%) | 0/29 (0.0%) | **RR 0.66** (0.03 to 15.85) | **0 fewer per 1,000** (from 0 fewer to 0 fewer) | ⨁⨁⨁⨁ HIGH |
| Severe Hypoglycemia (including studies with both arm zero events) | | | | | | | | | | | |
| 11 | randomised trials | not serious | not serious | not serious | not serious | none | 1/1969 (0.1%) | 0/736 (0.0%) | not estimable | **0 fewer per 1,000** (from 10 fewer to 10 more) | ⨁⨁⨁⨁ HIGH |

**CI:** Confidence interval; **RR:** Risk ratio

**Explanations**

a. Harms outcome in a placebo-controlled study

b. Attrition bias

c. Despite single study with severe hypoglycemia, 10 other studies reported zero severe events

**Question**: Monotherapy: SGLT2i compared to PBO in patients with T2D

| **Certainty assessment** | | | | | | | **№ of patients** | | **Effect** | | **Certainty** |
| --- | --- | --- | --- | --- | --- | --- | --- | --- | --- | --- | --- |
| **№ of studies** | **Study design** | **Risk of bias** | **Inconsistency** | **Indirectness** | **Imprecision** | **Other considerations** | **Monotherapy: SGLT2i** | **PBO** | **Relative (95% CI)** | **Absolute (95% CI)** |  |
| Any Hypoglycemia | | | | | | | | | | | |
| 19 | randomised trials | serious ^a^ | not serious | not serious | not serious | none | 94/4873 (1.9%) | 18/1774 (1.0%) | **RR 1.34** (0.83 to 2.15) | **3 more per 1,000** (from 2 fewer to 12 more) | ⨁⨁⨁◯ MODERATE |
| Any Hypoglycemia (including studies with both arm zero events) | | | | | | | | | | | |
| 19 | randomised trials | serious ^a^ | not serious | not serious | not serious | none | 94/4873 (1.9%) | 18/1774 (1.0%) | not estimable | **10 fewer per 1,000** (from 10 fewer to 0 fewer) | ⨁⨁⨁◯ MODERATE |
| Severe Hypoglycemia | | | | | | | | | | | |
| 1 | randomised trials | not serious | not serious | not serious | not serious ^b^ | none | 2/308 (0.6%) | 0/153 (0.0%) | **RR 2.49** (0.12 to 51.59) | **0 fewer per 1,000** (from 0 fewer to 0 fewer) | ⨁⨁⨁⨁ HIGH |
| Severe Hypoglycemia (including studies with both arm zero events) | | | | | | | | | | | |
| 19 | randomised trials | not serious | not serious | not serious | not serious | none | 2/4873 (0.0%) | 0/1774 (0.0%) | not estimable | **0 fewer per 1,000** (from 0 fewer to 0 fewer) | ⨁⨁⨁⨁ HIGH |

**CI:** Confidence interval; **RR:** Risk ratio

**Explanations**

a. Harms outcome in a placebo-controlled study

b. Despite only 1 study with severe hypoglycemia, 18 others reported zero severe events

**Question**: Metformin Background: DPP4i compared to PBO in patients with T2D

| **Certainty assessment** | | | | | | | **№ of patients** | | **Effect** | | **Certainty** |
| --- | --- | --- | --- | --- | --- | --- | --- | --- | --- | --- | --- |
| **№ of studies** | **Study design** | **Risk of bias** | **Inconsistency** | **Indirectness** | **Imprecision** | **Other considerations** | **Metformin Background: DPP4i** | **PBO** | **Relative (95% CI)** | **Absolute (95% CI)** |  |
| Any Hypoglycemia | | | | | | | | | | | |
| 22 | randomised trials | serious ^a^ | not serious | not serious | not serious | none | 104/5598 (1.9%) | 49/2770 (1.8%) | **RR 0.95** (0.67 to 1.35) | **1 fewer per 1,000** (from 6 fewer to 6 more) | ⨁⨁⨁◯ MODERATE |
| Any Hypoglycemia (including studies with both arm zero events) | | | | | | | | | | | |
| 29 | randomised trials | serious ^a^ | not serious | not serious | not serious | none | 104/6396 (1.6%) | 49/3283 (1.5%) | not estimable | **0 fewer per 1,000** (from 10 fewer to 0 fewer) | ⨁⨁⨁◯ MODERATE |
| Severe Hypoglycemia | | | | | | | | | | | |
| 5 | randomised trials | not serious | not serious | not serious | not serious ^b^ | none | 6/1318 (0.5%) | 4/621 (0.6%) | **RR 0.79** (0.23 to 2.70) | **1 fewer per 1,000** (from 5 fewer to 11 more) | ⨁⨁⨁⨁ HIGH |
| Severe Hypoglycemia (including studies with both arm zero events) | | | | | | | | | | | |
| 29 | randomised trials | not serious | not serious | not serious | not serious | none | 6/6396 (0.1%) | 4/3283 (0.1%) | not estimable | **0 fewer per 1,000** (from 0 fewer to 0 fewer) | ⨁⨁⨁⨁ HIGH |

**CI:** Confidence interval; **RR:** Risk ratio

**Explanations**

a. Harms outcome in a placebo-controlled study

b. Despite only 5 trials with severe events, 24 others reported zero severe events

**Question**: Metformin Background: GLP1RA compared to PBO in patients with T2D

| **Certainty assessment** | | | | | | | **№ of patients** | | **Effect** | | **Certainty** |
| --- | --- | --- | --- | --- | --- | --- | --- | --- | --- | --- | --- |
| **№ of studies** | **Study design** | **Risk of bias** | **Inconsistency** | **Indirectness** | **Imprecision** | **Other considerations** | **Metformin Background: GLP1RA** | **PBO** | **Relative (95% CI)** | **Absolute (95% CI)** |  |
| Any Hypoglycemia | | | | | | | | | | | |
| 10 | randomised trials | very serious ^a,b^ | not serious | not serious | not serious | none | 143/3062 (4.7%) | 23/863 (2.7%) | **RR 1.24** (0.80 to 1.91) | **6 more per 1,000** (from 5 fewer to 24 more) | ⨁⨁◯◯ LOW |
| Any Hypoglycemia (including studies with both arm zero events) | | | | | | | | | | | |
| 11 | randomised trials | very serious ^a,b^ | not serious | not serious | not serious | none | 143/3148 (4.5%) | 23/948 (2.4%) | not estimable | **10 fewer per 1,000** (from 20 fewer to 0 fewer) | ⨁⨁◯◯ LOW |
| Severe Hypoglycemia | | | | | | | | | | | |
| 1 | randomised trials | not serious | not serious | not serious | not serious ^c^ | none | 1/490 (0.2%) | 0/71 (0.0%) | **RR 0.44** (0.02 to 10.70) | **0 fewer per 1,000** (from 0 fewer to 0 fewer) | ⨁⨁⨁⨁ HIGH |
| Severe Hypoglycemia (including studies with both arm zero events) | | | | | | | | | | | |
| 11 | randomised trials | not serious | not serious | not serious | not serious | none | 1/3148 (0.0%) | 0/948 (0.0%) | not estimable | **0 fewer per 1,000** (from 0 fewer to 0 fewer) | ⨁⨁⨁⨁ HIGH |

**CI:** Confidence interval; **RR:** Risk ratio

**Explanations**

a. Harms outcome in a placebo-controlled study

b. Attrition bias

c. Despite only one study, 10 others reported zero severe hypoglycemia

**Question**: Metformin Background: SGLT2i compared to PBO in patients with T2D

| **Certainty assessment** | | | | | | | **№ of patients** | | **Effect** | | **Certainty** |
| --- | --- | --- | --- | --- | --- | --- | --- | --- | --- | --- | --- |
| **№ of studies** | **Study design** | **Risk of bias** | **Inconsistency** | **Indirectness** | **Imprecision** | **Other considerations** | **Metformin Background: SGLT2i** | **PBO** | **Relative (95% CI)** | **Absolute (95% CI)** |  |
| Any Hypoglycemia | | | | | | | | | | | |
| 15 | randomised trials | serious ^a^ | not serious | not serious | not serious | none | 141/4930 (2.9%) | 41/1634 (2.5%) | **RR 1.29** (0.91 to 1.83) | **7 more per 1,000** (from 2 fewer to 21 more) | ⨁⨁⨁◯ MODERATE |
| Any Hypoglycemia (including studies with both arm zero events) | | | | | | | | | | | |
| 18 | randomised trials | serious ^a^ | not serious | not serious | not serious | none | 141/5368 (2.6%) | 41/1833 (2.2%) | not estimable | **10 fewer per 1,000** (from 10 fewer to 0 fewer) | ⨁⨁⨁◯ MODERATE |
| Severe Hypoglycemia | | | | | | | | | | | |
| 2 | randomised trials | not serious | not serious | not serious | not serious ^b^ | none | 5/740 (0.7%) | 3/275 (1.1%) | **RR 0.46** (0.11 to 1.92) | **6 fewer per 1,000** (from 10 fewer to 10 more) | ⨁⨁⨁⨁ HIGH |
| Severe Hypoglycemia (including studies with both arm zero events) | | | | | | | | | | | |
| 18 | randomised trials | not serious | not serious | not serious | not serious | none | 5/5368 (0.1%) | 3/1833 (0.2%) | not estimable | **0 fewer per 1,000** (from 0 fewer to 0 fewer) | ⨁⨁⨁⨁ HIGH |

**CI:** Confidence interval; **RR:** Risk ratio

**Explanations**

a. Harms outcome in placebo-controlled study

b. Despite only 2 studies, 16 others reported zero severe hypoglycemia

**Question**: Non-Metformin Background: Dual Therapy compared to Monotherapy + PBO in patients with T2D

| **Certainty assessment** | | | | | | | **№ of patients** | | **Effect** | | **Certainty** |
| --- | --- | --- | --- | --- | --- | --- | --- | --- | --- | --- | --- |
| **№ of studies** | **Study design** | **Risk of bias** | **Inconsistency** | **Indirectness** | **Imprecision** | **Other considerations** | **Non-Metformin Background: Dual Therapy** | **Monotherapy + PBO** | **Relative (95% CI)** | **Absolute (95% CI)** |  |
| Any Hypoglycemia | | | | | | | | | | | |
| 1 | randomised trials | serious ^a^ | not serious | not serious | not serious | none | 0/182 (0.0%) | 1/93 (1.1%) | **RR 0.17** (0.01 to 4.16) | **9 fewer per 1,000** (from 11 fewer to 34 more) | ⨁⨁⨁◯ MODERATE |
| Any Hypoglycemia (including studies with both arm zero events) | | | | | | | | | | | |
| 5 | randomised trials | serious ^a^ | not serious | not serious | not serious | none | 0/551 (0.0%) | 1/402 (0.2%) | not estimable | **0 fewer per 1,000** (from 10 fewer to 10 more) | ⨁⨁⨁◯ MODERATE |
| Severe Hypoglycemia | | | | | | | | | | | |
| 0 | randomised trials | not serious | not serious | not serious | not serious ^b^ | none | 0/0 | 0/0 | not pooled | see comment | ⨁⨁⨁⨁ HIGH |
| Severe Hypoglycemia (including studies with both arm zero events) | | | | | | | | | | | |
| 5 | randomised trials | not serious | not serious | not serious | not serious | none | 0/551 (0.0%) | 0/402 (0.0%) | not estimable | **0 fewer per 1,000** (from 10 fewer to 10 more) | ⨁⨁⨁⨁ HIGH |

**CI:** Confidence interval; **RR:** Risk ratio

**Explanations**

a. Harms outcome in a placebo-controlled study

b. Despite zero studies included, 5 trials reported zero severe events

**Question**: Dual Initiation compared to PBO in patients with T2D

| **Certainty assessment** | | | | | | | **№ of patients** | | **Effect** | | **Certainty** |
| --- | --- | --- | --- | --- | --- | --- | --- | --- | --- | --- | --- |
| **№ of studies** | **Study design** | **Risk of bias** | **Inconsistency** | **Indirectness** | **Imprecision** | **Other considerations** | **Dual Initiation** | **PBO** | **Relative (95% CI)** | **Absolute (95% CI)** |  |
| Any Hypoglycemia | | | | | | | | | | | |
| 6 | randomised trials | serious ^a^ | not serious | not serious | not serious | none | 65/1477 (4.4%) | 9/738 (1.2%) | **RR 3.56** (1.79 to 7.10) | **31 more per 1,000** (from 10 more to 74 more) | ⨁⨁⨁◯ MODERATE |
| Any Hypoglycemia (including studies with both arm zero events) | | | | | | | | | | | |
| 6 | randomised trials | serious ^a^ | not serious | not serious | not serious | none | 65/1477 (4.4%) | 9/738 (1.2%) | not estimable | **30 fewer per 1,000** (from 50 fewer to 10 fewer) | ⨁⨁⨁◯ MODERATE |
| Severe Hypoglycemia | | | | | | | | | | | |
| 1 | randomised trials | not serious | not serious | not serious | not serious ^b^ | none | 2/194 (1.0%) | 0/97 (0.0%) | **RR 2.51** (0.12 to 51.83) | **0 fewer per 1,000** (from 0 fewer to 0 fewer) | ⨁⨁⨁⨁ HIGH |
| Severe Hypoglycemia (including studies with both arm zero events) | | | | | | | | | | | |
| 6 | randomised trials | not serious | not serious | not serious | not serious | none | 2/1477 (0.1%) | 0/738 (0.0%) | not estimable | **0 fewer per 1,000** (from 10 fewer to 0 fewer) | ⨁⨁⨁⨁ HIGH |

**CI:** Confidence interval; **RR:** Risk ratio

**Explanations**

a. Harms outcome in a placebo-controlled study

b. Despite only single study, 5 others reported zero severe events

**Question**: Triple Therapy compared to Dual Therapy + PBO in patients with T2D

| **Certainty assessment** | | | | | | | **№ of patients** | | **Effect** | | **Certainty** |
| --- | --- | --- | --- | --- | --- | --- | --- | --- | --- | --- | --- |
| **№ of studies** | **Study design** | **Risk of bias** | **Inconsistency** | **Indirectness** | **Imprecision** | **Other considerations** | **Triple Therapy** | **Dual Therapy + PBO** | **Relative (95% CI)** | **Absolute (95% CI)** |  |
| Any Hypoglycemia | | | | | | | | | | | |
| 8 | randomised trials | very serious ^a,b^ | not serious | not serious | not serious | none | 39/1534 (2.5%) | 23/1127 (2.0%) | **RR 1.13** (0.67 to 1.91) | **3 more per 1,000** (from 7 fewer to 19 more) | ⨁⨁◯◯ LOW |
| Any Hypoglycemia (including studies with both arm zero events) | | | | | | | | | | | |
| 8 | randomised trials | very serious ^a,b^ | not serious | not serious | not serious | none | 39/1534 (2.5%) | 23/1127 (2.0%) | not estimable | **0 fewer per 1,000** (from 20 fewer to 10 more) | ⨁⨁◯◯ LOW |
| Severe Hypoglycemia | | | | | | | | | | | |
| 3 | randomised trials | not serious | not serious | not serious | not serious ^c^ | none | 2/814 (0.2%) | 1/403 (0.2%) | **RR 0.72** (0.11 to 4.53) | **1 fewer per 1,000** (from 2 fewer to 9 more) | ⨁⨁⨁⨁ HIGH |
| Severe Hypoglycemia (including studies with both arm zero events) | | | | | | | | | | | |
| 7 | randomised trials | not serious | not serious | not serious | not serious | none | 2/1309 (0.2%) | 1/901 (0.1%) | not estimable | **0 fewer per 1,000** (from 10 fewer to 10 more) | ⨁⨁⨁⨁ HIGH |

**CI:** Confidence interval; **RR:** Risk ratio

**Explanations**

a. Harms outcome in a placebo-controlled study

b. Attrition bias

c. Despite only 3 trials, 4 others reported zero severe events
